# Supplementary material for: Accuracy and clinical effectiveness of risk prediction tools for pressure injury occurrence: An umbrella review
Source: PLoS Med. 2025 Feb 6;22(2):e1004518. doi: 10.1371/journal.pmed.1004518 (PMC11844857; doi:10.1371/journal.pmed.1004518)
Supplement: S1 Supporting Information — (PDF) [file pmed.1004518.s001.pdf]

S1 Supporting Information: Appendices

Appendix A: PRISMA-DTA Checklist 2

Appendix B: Description of search strategies 5

Appendix C: Data extraction form 24

Appendix D: AMSTAR-2 Methodology Quality Appraisal. Adapted for application to reviews of prognostic model and accuracy studies. 26

Appendix E: Detailed results tables 31

Table A. Full-text articles excluded, with reasons 31

Table B. Systematic review characteristics 35

Table C. AMSTAR-2 assessment results per review 42

Table D. Statistical synthesis results of accuracy (from all 16 reviews, that conducted meta-analysis or statistical synthesis), by prediction tool 46

Table E. Individual accuracy results of PI risk prediction tools (for which no meta-analysis was conducted*) 61

Table F. Results from all 11 included systematic reviews evaluating clinical effectiveness 70

References 76

Appendix A: PRISMA-DTA Checklist

Section/topic	#	PRISMA-DTA Checklist Item	Where reported (paragraph #)
**TITLE / ABSTRACT**			
Title	1	Identify the report as a systematic review (+/- meta-analysis) of ~~diagnostic test~~ accuracy ~~(DTA) studies~~ *reviews*	Title page and abstract
Abstract	2	Abstract: See PRISMA-DTA for abstracts.	Abstract
**INTRODUCTION**			
Rationale	3	Describe the rationale for the review in the context of what is already known.	Introduction (1-3)
Clinical role of index test	D1	State the scientific and clinical background, including the intended use and clinical role of the index test, and if applicable, the rationale for minimally acceptable test accuracy (or minimum difference in accuracy for comparative design).	Introduction (1-3)
Objectives	4	Provide an explicit statement of question(s) being addressed in terms of participants, index test(s), and target condition(s).	Introduction (4)
**METHODS**			
Protocol and registration	5	Indicate if a review protocol exists, if and where it can be accessed (e.g., Web address), and, if available, provide registration information including registration number.	Methods: Protocol registration and reporting of findings
Eligibility criteria	6	Specify study characteristics (participants, setting, index test(s), reference standard(s), target condition(s), and study design) and report characteristics (e.g., years considered, language, publication status) used as criteria for eligibility, giving rationale.	Methods: Eligibility criteria for this umbrella review
Information sources	7	Describe all information sources (e.g., databases with dates of coverage, contact with study authors to identify additional studies) in the search and date last searched.	Methods: Literature search
Search	8	Present full search strategies for all electronic databases and other sources searched, including any limits used, such that they could be repeated.	Appendix B
Study selection	9	State the process for selecting studies (i.e., screening, eligibility, included in systematic review, and, if applicable, included in the meta-analysis).	Methods: Literature search, Eligibility criteria
Data collection process	10	Describe method of data extraction from reports (e.g., piloted forms, independently, in duplicate) and any processes for obtaining and confirming data from investigators.	Methods: Data extraction and quality assessment (1)
Definitions for data extraction	11	Provide definitions used in data extraction and classifications of target condition(s), index test(s), reference standard(s) and other characteristics (e.g. study design, clinical setting).	Methods: Data extraction and quality assessment (1);

Section/topic	#	PRISMA-DTA Checklist Item	Where reported (paragraph #)
			Appendix C
Risk of bias and applicability	12	Describe methods used for assessing risk of bias in individual studies and concerns regarding the applicability to the review question.	Methods: Data extraction and quality assessment (2); Appendix D
Diagnostic accuracy measures	13	State the principal diagnostic accuracy measure(s) reported (e.g. sensitivity, specificity) and state the unit of assessment (e.g. per-patient, per-lesion).	Methods: Data extraction and quality assessment (1); Appendix C
Synthesis of results	14	Describe methods of handling data, combining results of studies and describing variability between studies. This could include, but is not limited to: a) handling of multiple definitions of target condition. b) handling of multiple thresholds of test positivity, c) handling multiple index test readers, d) handling of indeterminate test results, e) grouping and comparing tests, f) handling of different reference standards	Methods: Synthesis methods
Meta-analysis	D2	Report the statistical methods used for meta-analyses, if performed.	--
Additional analyses	16	Describe methods of additional analyses (e.g., sensitivity or subgroup analyses, meta-regression), if done, indicating which were pre-specified.	--
**RESULTS**			
Study selection	17	Provide numbers of studies screened, assessed for eligibility, included in the review (and included in meta-analysis, if applicable) with reasons for exclusions at each stage, ideally with a flow diagram.	Figure 1; Appendix E Table A
Study characteristics	18	For each included study provide citations and present key characteristics including: a) participant characteristics (presentation, prior testing), b) clinical setting, c) study design, d) target condition definition, e) index test, f) reference standard, g) sample size, h) funding sources	Table 1; Table 2; Table 4; Appendix E Table B
Risk of bias and applicability	19	Present evaluation of risk of bias and concerns regarding applicability for each study.	Figure 2; Appendix E Table C
Results of individual ~~studies~~ *reviews*	20	For each analysis in each ~~study~~ *review* (e.g. unique combination of index test, reference standard, and positivity threshold) report ~~2x2 data (TP, FP, FN, TN) with~~ estimates of diagnostic accuracy and confidence intervals, ~~ideally with a forest or receiver operator characteristic (ROC) plot.~~	Table 3; Appendix E Tables D-F

Section/topic	#	PRISMA-DTA Checklist Item	Where reported (paragraph #)
Synthesis of results	21	Describe test accuracy, including variability; if meta-analysis was done, include results and confidence intervals.	Results: Results from reviews evaluating the prognostic accuracy of risk prediction tools – Results from reviews evaluating the clinical effectiveness of risk prediction tools
Additional analysis	23	Give results of additional analyses, if done (e.g., sensitivity or subgroup analyses, meta-regression; analysis of index test: failure rates, proportion of inconclusive results, adverse events).	--
**DISCUSSION**			
Summary of evidence	24	Summarize the main findings including the strength of evidence.	Discussion (1);<br>Discussion: Prognostic accuracy of risk prediction tools – Clinical effectiveness of risk prediction scales
Limitations	25	Discuss limitations from included studies (e.g. risk of bias and concerns regarding applicability) and from the review process (e.g. incomplete retrieval of identified research).	Discussion (1);<br>Discussion: Strengths and limitations
Conclusions	26	Provide a general interpretation of the results in the context of other evidence. Discuss implications for future research and clinical practice (e.g. the intended use and clinical role of the index test).	Conclusions
**FUNDING**			
Funding	27	For the systematic review, describe the sources of funding and other support and the role of the funders.	Funding

Adapted From: McInnes MDF, Moher D, Thombs BD, McGrath TA, Bossuyt PM, The PRISMA-DTA Group (2018). Preferred Reporting Items for a Systematic Review and Meta-analysis of Diagnostic Test Accuracy Studies: The PRISMA-DTA Statement. JAMA. 2018 Jan 23;319(4):388-396. doi: 10.1001/jama.2017.19163.

Appendix B: Description of search strategies

ORIGINAL SEARCH: JAN 2023

Summary table of searches

Source	Results before deduplication	Results after deduplication
MEDLINE	1643	574
EMBASE	2060	1920
CINAHL	3720	3007
EPISTEMONIKOS	1194	574
GOOGLE SCHOLAR	357	226
**TOTAL**	**8974**	**6301**

SEARCH UPDATE: JUNE 2024

Searches run from 01/01/23 - 06/24

Summary table of searches

Source	Results before deduplication	Results after deduplication
MEDLINE	229	186
EMBASE	330	175
CINAHL	480	383
EPISTEMONIKOS	232	118
GOOGLE SCHOLAR	80	36
**TOTAL**	**1351**	**898**

Search approach and sources

Search concepts:

- **i.** pressure injury terms
- **ii.** systematic review terms
- **iii.** prediction model terms

Pressure injury (PI) terms were used from previous PI topic reviews and were developed in consultation with the wider review team and customer.

Established systematic review methodological filters were used in OVID Embase and OVID MEDLINE combining the appropriate McMasters best balance reviews filters[i] combining the appropriate McMasters best balance systematic reviews filters[ii] with the appropriate CADTH systematic review filter[iii].

A number of existing methodological filters are available for prediction/prognostic model terms. The effect of using different combinations of these filters has been tested in order to ensure retrieval of relevant literature at a manageable volume. This testing has informed the choice of prognostic search filters used (Geersing)[iv] Haynes Best Balance[v] and Ingui Best Balance[vi].

Searches were run in OVID MEDLINE, OVID Embase and EBSCO CINAHL Plus using the search concepts, systematic review and prediction/prognostic filters listed above or adaptations of these filters. No publication date or language restrictions were applied.

Epistemonikos was also searched using PI terms and key prognostic terms limited by publication type systematic review or broad synthesis. The Epistemonikos interface does not support the same search functionality available in OVID or EBSCO (for example adjacency operators are not supported). The Information Specialist ran several separate shorter searches to accommodate for the limitations of the interface. No publication date or language restrictions were applied.

In addition, Google Scholar was searched to pick up any potentially relevant papers not indexed in the other databases. The Google Scholar interface has limited search functionality. The Information Specialist ran several separate shorter searches to accommodate for the limitations of the interface. Searches were limited to review publication types published in the last eleven years only for pragmatic reasons as Google Scholar has poor export functionality.

"Connected papers" was also considered for inclusion, however it is a one 'seed tool', i.e., searching for one paper generates one map of connected papers. The platform is also only freely accessible for

---

[i]Search strategies for EMBASE in Ovid Syntax, In Health Information Research Unit Hedges project.Ontario:HIRU;2022: Health Information Research Unit - HIRU ~ Search Strategies for EMBASE in Ovid Syntax (mcmaster.ca) Accessed 2022-09-29
Search strategies for MEDLINE in Ovid Syntax, In Health Information Research Unit Hedges project.Ontario:HIRU;2022: Health Information Research Unit - HIRU ~ Search Strategies for MEDLINE in Ovid Syntax and the PubMed translation (mcmaster.ca) Accessed 2022-09-29.

[ii]Search strategies for EMBASE in Ovid Syntax, In Health Information Research Unit Hedges project.Ontario:HIRU;2022: Health Information Research Unit - HIRU ~ Search Strategies for EMBASE in Ovid Syntax (mcmaster.ca) Accessed 2022-09-29 and
Search strategies for MEDLINE in Ovid Syntax, In Health Information Research Unit Hedges project.Ontario:HIRU;2022: Health Information Research Unit - HIRU ~ Search Strategies for MEDLINE in Ovid Syntax and the PubMed translation (mcmaster.ca) Accessed 2022-09-29.

[iii] SR / MA / HTA / ITC - MEDLINE, Embase, PsycInfo. In: CADTH Search Filters Database. Ottawa: CADTH; 2022: https://searchfilters.cadth.ca/link/33. Accessed 2022-09-29.

[iv] Geersing GJ, Bouwmeester W, Zuithoff P, et al. Search filters for finding prognostic and diagnostic prediction studies in Medline to enhance systematic reviews. *PLoS One* 2012;7(2):e32844. doi:10.1371/journal.pone.0032844 [published Online First: 2012/03/07]

[v] Wilczynski NL, Haynes RB. Optimal search strategies for detecting clinically sound prognostic studies EMBASE: and analytic survey. *J Am Med Inform Assoc* 2005;12(4):481-5. doi: 10.1197/jamia.M1752 [published Online First: 2005/04/02]

[vi] Ingui BJ, Rogers MA. Searching for clinical prediction rules in MEDLINE. *J Am Med Inform Assoc* 2001;8(4):391-7. doi: 10.1136/jamia.2001.0080391 [published Online First: 2001/06/22]

searching five ‘seed’ papers a month, which appears to be more of a limitation than would be beneficial for this set of reviews.

MEDLINE ALL (OVID)

Date run: 31/01/23

Database: Ovid MEDLINE(R) ALL <1946 to January 30, 2023>

Search Strategy:

----

1 (decubit* or bedsore* or bed-sore* or pressure-ulcer* or pressure-wound*).tw. (17989)

2 ((pressure* or bed or bedbound or bed-bound or bedridden or bed-ridden or deep tissue* or deep-tissue) adj3 (wound* or ulcer* or sore* or injur* or lesion*)).tw. (22136)

3 exp pressure ulcer/ or pressure/ae (15198)

4 1 or 2 or 3 (33198)

5 ((supine or immobil*) adj3 (heal or healing or heals or healed or dress*)).tw. (220)

6 ((supine or immobil*) adj3 (wound* or ulcer* or sore* or injur* or lesion*)).tw. (876)

7 ((pressure or bedbound or bedridden or bed-bound or bed-ridden or deep tissue or deep-tissue) adj3 (heal or healing or heals or healed or dress*)).tw. (1863)

8 5 or 6 or 7 (2923)

9 4 or 8 (34931)

10 (systematic review or meta-analysis).pt. (299072)

11 review.pt. (3115171)

12 search:.tw. (617577)

13 meta-analys:.mp. (291117)

14 meta-analysis/ or systematic review/ or systematic reviews as topic/ or meta-analysis as topic/ or "meta analysis (topic)"/ or "systematic review (topic)"/ or exp technology assessment, biomedical/ or network meta-analysis/ (336517)

15 ((systematic* adj3 (review* or overview*)) or (methodologic* adj3 (review* or overview*))).ti,ab,kf. (303236)

16 ((quantitative adj3 (review* or overview* or synthes*)) or (research adj3 (integrati* or overview*))).ti,ab,kf. (15021)

17 ((integrative adj3 (review* or overview*)) or (collaborative adj3 (review* or overview*)) or (pool* adj3 analy*)).ti,ab,kf. (37395)

18 (data synthes* or data extraction* or data abstraction*).ti,ab,kf. (38583)

19 (handsearch* or hand search*).ti,ab,kf. (10921)

20 (mantel haenszel or peto or der simonian or dersimonian or fixed effect* or latin square*).ti,ab,kf. (34465)

21 (met analy* or metanaly* or technology assessment* or HTA or HTAs or technology overview* or technology appraisal*).ti,ab,kf. (11813)

22 (meta regression* or metaregression*).ti,ab,kf. (13870)

23 (meta-analy* or metaanaly* or systematic review* or biomedical technology assessment* or bio-medical technology assessment*).mp,hw. (446528)

24 (medline or cochrane or pubmed or medlars or embase or cinahl).ti,ab,hw. (325867)

25 (cochrane or (health adj2 technology assessment) or evidence report).jw. (21207)

26 (comparative adj3 (efficacy or effectiveness)).ti,ab,kf. (17070)

27 (outcomes research or relative effectiveness).ti,ab,kf. (11017)

28 ((indirect or indirect treatment or mixed-treatment or bayesian) adj3 comparison*).ti,ab,kf. (4214)

29 (multi* adj3 treatment adj3 comparison*).ti,ab,kf. (287)

30 (mixed adj3 treatment adj3 (meta-analy* or metaanaly*)).ti,ab,kf. (177)

31 umbrella review*.ti,ab,kf. (1305)

32 (multi* adj2 paramet* adj2 evidence adj2 synthesis).ti,ab,kf. (13)

33 (multiparamet* adj2 evidence adj2 synthesis).ti,ab,kf. (18)

34 (multi-paramet* adj2 evidence adj2 synthesis).ti,ab,kf. (11)

35 or/10-34 (3717455)

36 predict:.tw. or validat:.mp. or develop.tw. (3128744)

37 (stratification or ROC curve).ti,ab. or exp ROC curve/ or discriminat$.ti,ab. or c-statistic.ti,ab. or "Area under the curve".ti,ab. or AUC.ti,ab. or Calibration.ti,ab. or indices.ti,ab. or algorithm.ti,ab. or multivaria$.mp. (1567005)

38 Validat*.mp. or Predict$.ti. or Rule*.mp. or (Predict* and (Outcome* or Risk* or Model$)).mp. or ((History or Variable$ or Criteria or Scor$ or Characteristic$ or Finding$ or Factor$) and (Predict$ or Model$ or Decision$ or Identif$ or Prognos$)).mp. or (Decision$.mp. and ((Model$ or Clinical$).mp. or Logistic Models/)) or (Prognostic and (History or Variable$ or Criteria or Scor$ or Characteristic$ or Finding$ or Factor$ or Model$)).mp. [mp=title, book title, abstract, original title, name of substance word, subject heading word, floating sub-heading word, keyword heading word, organism supplementary concept word, protocol supplementary concept word, rare disease supplementary concept word, unique identifier, synonyms] (5961362)

39 36 or 37 (6673746)

40 39 or 38 (7368991)

41 9 and 35 and 40 (1643)

MEDLINE ALL (OVID)

Date run: 20/06/24

Database: Ovid MEDLINE(R) ALL <1946 to June 20, 2024>

Search Strategy:

----

1 (decubit* or bedsore* or bed-sore* or pressure-ulcer* or pressure-wound*).tw.

2 ((pressure* or bed or bedbound or bed-bound or bedridden or bed-ridden or deep tissue* or deep-tissue) adj3 (wound* or ulcer* or sore* or injur* or lesion*)).tw.

3 exp pressure ulcer/ or pressure/ae

4 1 or 2 or 3

5 ((supine or immobil*) adj3 (heal or healing or heals or healed or dress*)).tw.

6 ((supine or immobil*) adj3 (wound* or ulcer* or sore* or injur* or lesion*)).tw.

7 ((pressure or bedbound or bedridden or bed-bound or bed-ridden or deep tissue or deep-tissue) adj3 (heal or healing or heals or healed or dress*)).tw.

8 5 or 6 or 7

9 4 or 8

10 (systematic review or meta-analysis).pt.

11 review.pt.

12 search:.tw.

13 meta-analys:.mp.

14 meta-analysis/ or systematic review/ or systematic reviews as topic/ or meta-analysis as topic/ or "meta analysis (topic)"/ or "systematic review (topic)"/ or exp technology assessment, biomedical/ or network meta-analysis/

15 ((systematic* adj3 (review* or overview*)) or (methodologic* adj3 (review* or overview*))).ti,ab,kf.

16 ((quantitative adj3 (review* or overview* or synthes*)) or (research adj3 (integrati* or overview*))).ti,ab,kf.

17 ((integrative adj3 (review* or overview*)) or (collaborative adj3 (review* or overview*)) or (pool* adj3 analy*)).ti,ab,kf.

18 (data synthes* or data extraction* or data abstraction*).ti,ab,kf.

19 (handsearch* or hand search*).ti,ab,kf.

20 (mantel haenszel or peto or der simonian or dersimonian or fixed effect* or latin square*).ti,ab,kf.

21 (met analy* or metanaly* or technology assessment* or HTA or HTAs or technology overview* or technology appraisal*).ti,ab,kf.

22 (meta regression* or metaregression*).ti,ab,kf.

23 (meta-analy* or metaanaly* or systematic review* or biomedical technology assessment* or bio-medical technology assessment*).mp,hw.

24 (medline or cochrane or pubmed or medlars or embase or cinahl).ti,ab,hw.

25 (cochrane or (health adj2 technology assessment) or evidence report).jw.

26 (comparative adj3 (efficacy or effectiveness)).ti,ab,kf.

27 (outcomes research or relative effectiveness).ti,ab,kf.

28 ((indirect or indirect treatment or mixed-treatment or bayesian) adj3 comparison*).ti,ab,kf.

29 (multi* adj3 treatment adj3 comparison*).ti,ab,kf.

30 (mixed adj3 treatment adj3 (meta-analy* or metaanaly*)).ti,ab,kf.

31 umbrella review*.ti,ab,kf.

32 (multi* adj2 paramet* adj2 evidence adj2 synthesis).ti,ab,kf.

33 (multiparamet* adj2 evidence adj2 synthesis).ti,ab,kf.

34 (multi-paramet* adj2 evidence adj2 synthesis).ti,ab,kf.

35 or/10-34

36 predict:.tw. or validat:.mp. or develop.tw.

37 (stratification or ROC curve).ti,ab. or exp ROC curve/ or discriminat$.ti,ab. or c-statistic.ti,ab. or "Area under the curve".ti,ab. or AUC.ti,ab. or Calibration.ti,ab. or indices.ti,ab. or algorithm.ti,ab. or multivaria$.mp.

38 Validat*.mp. or Predict$.ti. or Rule*.mp. or (Predict* and (Outcome* or Risk* or Model$)).mp. or ((History or Variable$ or Criteria or Scor$ or Characteristic$ or Finding$ or Factor$) and (Predict$ or Model$ or Decision$ or Identif$ or Prognos$)).mp. or (Decision$.mp. and ((Model$ or Clinical$).mp. or Logistic Models/)) or (Prognostic and (History or Variable$ or Criteria or Scor$ or Characteristic$ or Finding$ or Factor$ or Model$)).mp. [mp=title, book title, abstract, original title, name of substance word, subject heading word, floating sub-heading word, keyword heading word, organism supplementary concept word, protocol supplementary concept word, rare disease supplementary concept word, unique identifier, synonyms]

39 36 or 37

40 39 or 38

41 9 and 35 and 40

42 limit 41 to dt=20230101-20241231

43 limit 41 to ez=20230101-20241231

44 limit 42 to da=20230101-20241231

45 42 or 43 or 44

Embase (OVID)

Date run: 31/01/23

Database: Embase <1974 to 2023 January 30>

Search Strategy:

----

1 (decubit* or bedsore* or bed-sore* or pressure-ulcer* or pressure-wound*).tw. (24186)

2 ((pressure* or bed or bedbound or bed-bound or bedridden or bed-ridden or deep tissue* or deep-tissue) adj3 (wound* or ulcer* or sore* or injur* or lesion*)).tw. (28692)

3 exp decubitus/ (24330)

4 1 or 2 or 3 (45109)

5 ((supine or immobil*) adj3 (heal or healing or heals or healed or dress*)).tw. (279)

6 ((supine or immobil*) adj3 (wound* or ulcer* or sore* or injur* or lesion*)).tw. (1181)

7 ((pressure or bedbound or bedridden or bed-bound or bed-ridden or deep tissue or deep-tissue) adj3 (heal or healing or heals or healed or dress*)).tw. (2463)

8 5 or 6 or 7 (3873)

9 4 or 8 (47397)

10 (systematic review or meta-analysis).pt. (0)

11 review.pt. (3006963)

12 search:.tw. (777617)

13 meta-analys:.mp. (420287)

14 meta-analysis/ or systematic review/ or systematic reviews as topic/ or meta-analysis as topic/ or "meta analysis (topic)"/ or "systematic review (topic)"/ or exp technology assessment, biomedical/ or network meta-analysis/ (600348)

15 ((systematic* adj3 (review* or overview*)) or (methodologic* adj3 (review* or overview*))).ti,ab,kf. (374423)

16 ((quantitative adj3 (review* or overview* or synthes*)) or (research adj3 (integrati* or overview*))).ti,ab,kf. (17492)

17 ((integrative adj3 (review* or overview*)) or (collaborative adj3 (review* or overview*)) or (pool* adj3 analy*)).ti,ab,kf. (53239)

18 (data synthes* or data extraction* or data abstraction*).ti,ab,kf. (47755)

19 (handsearch* or hand search*).ti,ab,kf. (13352)

20 (mantel haenszel or peto or der simonian or dersimonian or fixed effect* or latin square*).ti,ab,kf. (45672)

21 (met analy* or metanaly* or technology assessment* or HTA or HTAs or technology overview* or technology appraisal*).ti,ab,kf. (19758)

22 (meta regression* or metaregression*).ti,ab,kf. (17239)

23 (meta-analy* or metaanaly* or systematic review* or biomedical technology assessment* or bio-medical technology assessment*).mp,hw. (709507)

24 (medline or cochrane or pubmed or medlars or embase or cinahl).ti,ab,hw. (427516)

25 (cochrane or (health adj2 technology assessment) or evidence report).jw. (30592)

26 (comparative adj3 (efficacy or effectiveness)).ti,ab,kf. (25197)

27 (outcomes research or relative effectiveness).ti,ab,kf. (15991)

28 ((indirect or indirect treatment or mixed-treatment or bayesian) adj3 comparison*).ti,ab,kf. (7335)

29 (multi* adj3 treatment adj3 comparison*).ti,ab,kf. (423)

30 (mixed adj3 treatment adj3 (meta-analy* or metaanaly*)).ti,ab,kf. (256)

31 umbrella review*.ti,ab,kf. (1367)

32 (multi* adj2 paramet* adj2 evidence adj2 synthesis).ti,ab,kf. (28)

33 (multiparamet* adj2 evidence adj2 synthesis).ti,ab,kf. (21)

34 (multi-paramet* adj2 evidence adj2 synthesis).ti,ab,kf. (23)

35 or/10-34 (3937855)

36 validat:.mp. or index.tw. or model.tw. (5261274)

37 (stratification or ROC curve).ti,ab. or exp receiver operating characteristic/ or discriminat$.ti,ab. or c-statistic.ti,ab. or "Area under the curve".ti,ab. or AUC.ti,ab. or Calibration.ti,ab. or indices.ti,ab. or algorithm.ti,ab. or multivaria$.mp. (2151295)

38 Validat*.mp. or Predict$.ti. or Rule*.mp. or (Predict* and (Outcome* or Risk* or Model$)).mp. or ((History or Variable$ or Criteria or Scor$ or Characteristic$ or Finding$ or Factor$) and (Predict$ or Model$ or Decision$ or Identif$ or Prognos$)).mp. or (Decision$.mp. and ((Model$ or Clinical$).mp. or Statistical model/)) or (Prognostic and (History or Variable$ or Criteria or Scor$ or Characteristic$ or Finding$ or Factor$ or Model$)).mp. [mp=title, abstract, heading word, drug trade name, original title, device manufacturer, drug manufacturer, device trade name, keyword heading word, floating subheading word, candidate term word] (8141367)

39 36 or 37 (9046587)

40 39 or 38 (10998837)

47 9 and 35 and 40 (2060)

Embase (OVID)

Date run: 20/06/24

Database: Embase <1974 to 2024 June 20>

Search Strategy:

----

1 (decubit* or bedsore* or bed-sore* or pressure-ulcer* or pressure-wound*).tw.

2 ((pressure* or bed or bedbound or bed-bound or bedridden or bed-ridden or deep tissue* or deep-tissue) adj3 (wound* or ulcer* or sore* or injur* or lesion*)).tw.

3 exp decubitus/

4 1 or 2 or 3

5 ((supine or immobil*) adj3 (heal or healing or heals or healed or dress*)).tw.

6 ((supine or immobil*) adj3 (wound* or ulcer* or sore* or injur* or lesion*)).tw.

7 ((pressure or bedbound or bedridden or bed-bound or bed-ridden or deep tissue or deep-tissue) adj3 (heal or healing or heals or healed or dress*)).tw.

8 5 or 6 or 7

9 4 or 8

10 (systematic review or meta-analysis).pt.

11 review.pt.

12 search:.tw.

13 meta-analys:.mp.

14 meta-analysis/ or systematic review/ or systematic reviews as topic/ or meta-analysis as topic/ or "meta analysis (topic)"/ or "systematic review (topic)"/ or exp technology assessment, biomedical/ or network meta-analysis/

15 ((systematic* adj3 (review* or overview*)) or (methodologic* adj3 (review* or overview*))).ti,ab,kf.

16 ((quantitative adj3 (review* or overview* or synthes*)) or (research adj3 (integrati* or overview*))).ti,ab,kf.

17 ((integrative adj3 (review* or overview*)) or (collaborative adj3 (review* or overview*)) or (pool* adj3 analy*)).ti,ab,kf.

18 (data synthes* or data extraction* or data abstraction*).ti,ab,kf.

19 (handsearch* or hand search*).ti,ab,kf.

20 (mantel haenszel or peto or der simonian or dersimonian or fixed effect* or latin square*).ti,ab,kf.

21 (met analy* or metanaly* or technology assessment* or HTA or HTAs or technology overview* or technology appraisal*).ti,ab,kf.

22 (meta regression* or metaregression*).ti,ab,kf.

23 (meta-analy* or metaanaly* or systematic review* or biomedical technology assessment* or bio-medical technology assessment*).mp,hw.

24 (medline or cochrane or pubmed or medlars or embase or cinahl).ti,ab,hw.

25 (cochrane or (health adj2 technology assessment) or evidence report).jw.

26 (comparative adj3 (efficacy or effectiveness)).ti,ab,kf.

27 (outcomes research or relative effectiveness).ti,ab,kf.

28 ((indirect or indirect treatment or mixed-treatment or bayesian) adj3 comparison*).ti,ab,kf.

29 (multi* adj3 treatment adj3 comparison*).ti,ab,kf.

30 (mixed adj3 treatment adj3 (meta-analy* or metaanaly*)).ti,ab,kf.

31 umbrella review*.ti,ab,kf.

32 (multi* adj2 paramet* adj2 evidence adj2 synthesis).ti,ab,kf.

33 (multiparamet* adj2 evidence adj2 synthesis).ti,ab,kf.

34 (multi-paramet* adj2 evidence adj2 synthesis).ti,ab,kf.

35 or/10-34

36 validat:.mp. or index.tw. or model.tw.

37 (stratification or ROC curve).ti,ab. or exp receiver operating characteristic/ or discriminat$.ti,ab. or c-statistic.ti,ab. or "Area under the curve".ti,ab. or AUC.ti,ab. or Calibration.ti,ab. or indices.ti,ab. or algorithm.ti,ab. or multivaria$.mp.

38 Validat*.mp. or Predict$.ti. or Rule*.mp. or (Predict* and (Outcome* or Risk* or Model$)).mp. or ((History or Variable$ or Criteria or Scor$ or Characteristic$ or Finding$ or Factor$) and (Predict$ or Model$ or Decision$ or Identif$ or Prognos$)).mp. or (Decision$.mp. and ((Model$ or Clinical$).mp. or Statistical model/)) or (Prognostic and (History or Variable$ or Criteria or Scor$ or Characteristic$ or Finding$ or Factor$ or Model$)).mp. [mp=title, abstract, heading word, drug trade name, original title, device manufacturer, drug manufacturer, device trade name, keyword heading word, floating subheading word, candidate term word] (8141367)

39 36 or 37

40 39 or 38

47 9 and 35 and 40

48 limit 47 to dc=20230101-20243112

CINAHL PLUS (EBSCOhost)
SEARCH DATE 02/02/23

S1 (((TI decubit* OR AB decubit*) OR (TI bedsore* OR AB bedsore*) OR (TI bed-sore* OR AB bed-sore*) OR (TI pressure-ulcer* OR AB pressure-ulcer*) OR (TI pressure-wound* OR AB pressure-wound*))) OR ((((TI pressure* OR AB pressure*) OR (TI bed OR AB bed) OR (TI bedbound OR AB bedbound) OR (TI bed-bound OR AB bed-bound) OR (TI bedridden OR AB bedridden) OR (TI bed-ridden OR AB bed-ridden) OR (TI "deep tissue*" OR AB "deep tissue*") OR (TI deep-tissue OR AB deep-tissue)) N3 ((TI wound* OR AB wound*) OR (TI ulcer* OR AB ulcer*) OR (TI sore* OR AB sore*) OR (TI injur* OR AB injur*) OR (TI lesion* OR AB lesion*)))) OR ((MH "pressure ulcer"+) OR (MH pressure)) Expanders - Apply equivalent subjects (28 401)

S2 (((TI heal OR AB heal) OR (TI healing OR AB healing) OR (TI heals OR AB heals) OR (TI healed OR AB healed) OR (TI dress* OR AB dress*)))) OR ((((TI supine OR AB supine) OR (TI immobil* OR AB immobil*)) N3 ((TI wound* OR AB wound*) OR (TI ulcer* OR AB ulcer*) OR (TI sore* OR AB sore*) OR (TI injur* OR AB injur*) OR (TI lesion* OR AB lesion*)))) OR ((((TI pressure OR AB pressure) OR (TI bedbound OR AB bedbound) OR (TI bedridden OR AB bedridden) OR (TI bed-bound OR AB bed-bound) OR (TI bed-ridden OR AB bed-ridden) OR (TI "deep tissue" OR AB "deep tissue") OR (TI deep-tissue OR AB deep-tissue)) N3 ((TI heal OR AB heal) OR (TI healing OR AB healing) OR (TI heals OR AB heals) OR (TI healed OR AB healed) OR (TI dress* OR AB dress*)))) Expanders – Apply equivalent subjects (72 286)

S3 S1 OR S2 Expanders - Apply equivalent subjects (96245)

S4 ((PT "systematic review" OR PT meta-analysis)) OR ((MH meta-analysis) OR (MH "systematic review") OR (MH "systematic reviews as topic") OR (MH "meta-analysis as topic") OR (MH "meta analysis (topic)") OR (MH "systematic review (topic)") OR (MH "technology assessment, biomedical"+) OR (MH "network meta-analysis")) OR ((((TI systematic* OR AB systematic* OR SU systematic*) N3 ((TI review* OR AB review* OR SU review*) OR (TI overview* OR AB overview* OR SU overview*))) OR ((TI methodologic* OR AB methodologic* OR SU methodologic*) N3 ((TI review* OR AB review* OR SU review*) OR (TI overview* OR AB overview* OR SU overview*))))) OR ((((TI quantitative OR AB quantitative OR SU quantitative) N3 ((TI review* OR AB review* OR SU review*) OR (TI overview* OR AB overview* OR SU overview*) OR (TI synthes* OR AB synthes* OR SU synthes*))) OR ((TI research OR AB research OR SU research) N3 ((TI integrati* OR AB integrati* OR SU integrati*) OR (TI overview* OR AB overview* OR SU overview*))))) OR ((((TI integrative OR AB integrative OR SU integrative) N3 ((TI review* OR AB review* OR SU review*) OR (TI overview* OR AB overview* OR SU overview*))) OR ((TI collaborative OR AB collaborative OR SU collaborative) N3 ((TI review* OR AB review* OR SU review*) OR (TI overview* OR AB overview* OR SU overview*))) OR ((TI pool* OR AB pool* OR SU pool*) N3 (TI analy* OR AB analy* OR SU analy*)))) OR (((TI "data synthes*" OR AB "data synthes*" OR SU "data synthes*") OR (TI "data extraction*" OR AB "data extraction*" OR SU "data extraction*") OR (TI "data abstraction*" OR AB "data abstraction*" OR SU "data abstraction*"))) OR (((TI handsearch* OR AB handsearch* OR SU handsearch*) OR (TI "hand search*" OR AB "hand search*" OR SU "hand search*"))) OR (((TI "mantel haenszel" OR AB "mantel haenszel" OR SU "mantel haenszel") OR (TI peto OR AB peto OR SU peto) OR (TI "der simonian" OR AB "der simonian" OR SU "der simonian") OR (TI dersimonian OR AB dersimonian OR SU dersimonian) OR (TI "fixed effect*" OR AB "fixed effect*" OR SU "fixed effect*") OR (TI "latin square*" OR AB "latin square*" OR SU "latin square*"))) OR (((TI "met analy*" OR AB "met analy*" OR SU "met analy*") OR (TI metanaly* OR AB metanaly* OR SU metanaly*) OR (TI "technology assessment*" OR AB "technology assessment*" OR SU "technology assessment*") OR (TI HTA OR AB HTA OR SU HTA) OR (TI HTAs OR AB HTAs OR SU HTAs) OR (TI "technology overview*" OR AB

"technology overview*" OR SU "technology overview*") OR (TI "technology appraisal*" OR AB "technology appraisal*" OR SU "technology appraisal*"))) OR (((TI "meta regression*" OR AB "meta regression*" OR SU "meta regression*") OR (TI metaregression* OR AB metaregression* OR SU metaregression*))) OR ((meta-analy* OR metaanaly* OR "systematic review*" OR "biomedical technology assessment*" OR "bio-medical technology assessment*") ,hw.) OR (((TI medline OR AB medline) OR (TI cochrane OR AB cochrane) OR (TI pubmed OR AB pubmed) OR (TI medlars OR AB medlars) OR (TI embase OR AB embase) OR (TI cinahl OR AB cinahl)) ,hw.) Expanders - Apply equivalent subjects (237782)

S5 ((cochrane OR (health N2 "technology assessment") OR "evidence report") .jw.) OR (((TI comparative OR AB comparative OR SU comparative) N3 ((TI efficacy OR AB efficacy OR SU efficacy) OR (TI effectiveness OR AB effectiveness OR SU effectiveness)))) OR (((TI "outcomes research" OR AB "outcomes research" OR SU "outcomes research") OR (TI "relative effectiveness" OR AB "relative effectiveness" OR SU "relative effectiveness"))) OR ((((TI indirect OR AB indirect OR SU indirect) OR (TI "indirect treatment" OR AB "indirect treatment" OR SU "indirect treatment") OR (TI mixed-treatment OR AB mixed-treatment OR SU mixed-treatment) OR (TI bayesian OR AB bayesian OR SU bayesian)) N3 (TI comparison* OR AB comparison* OR SU comparison*))) OR (((TI multi* OR AB multi* OR SU multi*) N3 (TI treatment OR AB treatment OR SU treatment) N3 (TI comparison* OR AB comparison* OR SU comparison*))) OR ((TI "umbrella review*" OR AB "umbrella review*" OR SU "umbrella review*")) OR (((TI multi* OR AB multi* OR SU multi*) N2 (TI paramet* OR AB paramet* OR SU paramet*) N2 (TI evidence OR AB evidence OR SU evidence) N2 (TI synthesis OR AB synthesis OR SU synthesis))) OR (((TI multiparamet* OR AB multiparamet* OR SU multiparamet*) N2 (TI evidence OR AB evidence OR SU evidence) N2 (TI synthesis OR AB synthesis OR SU synthesis))) OR (((TI multi-paramet* OR AB multi-paramet* OR SU multi-paramet*) N2 (TI evidence OR AB evidence OR SU evidence) N2 (TI synthesis OR AB synthesis OR SU synthesis)) Expanders - Apply equivalent subjects (19108)

S6 ((TI search: OR AB search:)) Expanders - Apply equivalent subjects (123419)

S7 PT review Expanders - Apply equivalent subjects (356003)

S8 S4 OR S5 OR S6 OR S7 Expanders - Apply equivalent subjects (645360)

S9 S3 AND S8 Expanders - Apply equivalent subjects (10379)

S10 (TX validat*) or (TI index or model) or (AB index or model) Expanders - Apply equivalent subjects (1286240)

S11 TI (stratification or "ROC curve" or discriminat" or c-statistic" or "Area under the curve" or AUC or Calibration* or indices* or algorithm* or multivaria*) OR AB (stratification or "ROC curve" or discriminat" or c-statistic" or "Area under the curve" or AUC or Calibration* or indices* or algorithm* or multivaria*) Expanders - Apply equivalent subjects (294871)

S12 (MH "ROC Curve") Expanders - Apply equivalent subjects (33393)

S13 TI Validat* or Predict* or Rule* or (Predict* and (Outcome* or Risk* or Model*)) or ((History or Variable* or Criteria or Scor* or Characteristic* or Finding* or Factor*) and (Predict* or Model* or Decision* or Identif* or Prognos*)) or (Decision* and ((Model* or Clinical*) or (Prognostic and (History or Variable* or Criteria or Scor* or Characteristic* or Finding* or Factor* or Model*)) Expanders - Apply equivalent subjects (144228)

S14 AB Validat* or Predict* or Rule* or (Predict* and (Outcome* or Risk* or Model*)) or ((History or Variable* or Criteria or Scor* or Characteristic* or Finding* or Factor*) and (Predict* or Model* or Decision* or Identif* or Prognos*)) or (Decision* and ((Model* or Clinical*) or (Prognostic and (History or Variable* or Criteria or Scor* or Characteristic* or Finding* or Factor* or Model*)) Expanders - Apply equivalent subjects (1482692)

S15 (MH "Models, Statistical+") Expanders - Apply equivalent subjects (40243)

S16 S10 OR S11 OR S12 OR S13 OR S14 OR S15 Expanders - Apply equivalent subjects (2119713)

S17 S9 AND S16 (3720)

CINAHL PLUS (EBSCOhost)

SEARCH DATE 21/06/24

S1 (((TI decubit* OR AB decubit*) OR (TI bedsore* OR AB bedsore*) OR (TI bed-sore* OR AB bed-sore*) OR (TI pressure-ulcer* OR AB pressure-ulcer*) OR (TI pressure-wound* OR AB pressure-wound*))) OR ((((TI pressure* OR AB pressure*) OR (TI bed OR AB bed) OR (TI bedbound OR AB bedbound) OR (TI bed-bound OR AB bed-bound) OR (TI bedridden OR AB bedridden) OR (TI bed-ridden OR AB bed-ridden) OR (TI "deep tissue*" OR AB "deep tissue*") OR (TI deep-tissue OR AB deep-tissue)) N3 ((TI wound* OR AB wound*) OR (TI ulcer* OR AB ulcer*) OR (TI sore* OR AB sore*) OR (TI injur* OR AB injur*) OR (TI lesion* OR AB lesion*)))) OR ((MH "pressure ulcer"+) OR (MH pressure)) Expanders - Apply equivalent subjects

S2 (((TI heal OR AB heal) OR (TI healing OR AB healing) OR (TI heals OR AB heals) OR (TI healed OR AB healed) OR (TI dress* OR AB dress*)))) OR ((((TI supine OR AB supine) OR (TI immobil* OR AB immobil*)) N3 ((TI wound* OR AB wound*) OR (TI ulcer* OR AB ulcer*) OR (TI sore* OR AB sore*) OR (TI injur* OR AB injur*) OR (TI lesion* OR AB lesion*)))) OR ((((TI pressure OR AB pressure) OR (TI bedbound OR AB bedbound) OR (TI bedridden OR AB bedridden) OR (TI bed-bound OR AB bed-bound) OR (TI bed-ridden OR AB bed-ridden) OR (TI "deep tissue" OR AB "deep tissue") OR (TI deep-tissue OR AB deep-tissue)) N3 ((TI heal OR AB heal) OR (TI healing OR AB healing) OR (TI heals OR AB heals) OR (TI healed OR AB healed) OR (TI dress* OR AB dress*)))) Expanders – Apply equivalent subjects

S3 S1 OR S2 Expanders - Apply equivalent subjects

S4 ((PT "systematic review" OR PT meta-analysis)) OR ((MH meta-analysis) OR (MH "systematic review") OR (MH "systematic reviews as topic") OR (MH "meta-analysis as topic") OR (MH "meta analysis (topic)") OR (MH "systematic review (topic)") OR (MH "technology assessment, biomedical"+) OR (MH "network meta-analysis")) OR ((((TI systematic* OR AB systematic* OR SU systematic*) N3 ((TI review* OR AB review* OR SU review*) OR (TI overview* OR AB overview* OR SU overview*))) OR ((TI methodologic* OR AB methodologic* OR SU methodologic*) N3 ((TI review* OR AB review* OR SU review*) OR (TI overview* OR AB overview* OR SU overview*))))) OR ((((TI quantitative OR AB quantitative OR SU quantitative) N3 ((TI review* OR AB review* OR SU review*) OR (TI overview* OR AB overview* OR SU overview*) OR (TI synthes* OR AB synthes* OR SU synthes*))) OR ((TI research OR AB research OR SU research) N3 ((TI integrati* OR AB integrati* OR SU integrati*) OR (TI overview* OR AB overview* OR SU overview*))))) OR ((((TI integrative OR AB integrative OR SU integrative) N3 ((TI review* OR AB review* OR SU review*) OR (TI overview* OR AB overview* OR SU overview*))) OR ((TI collaborative OR AB collaborative OR SU collaborative) N3

((TI review* OR AB review* OR SU review*) OR (TI overview* OR AB overview* OR SU overview*))) OR ((TI pool* OR AB pool* OR SU pool*) N3 (TI analy* OR AB analy* OR SU analy*)))) OR (((TI "data synthes*" OR AB "data synthes*" OR SU "data synthes*") OR (TI "data extraction*" OR AB "data extraction*" OR SU "data extraction*") OR (TI "data abstraction*" OR AB "data abstraction*" OR SU "data abstraction*"))) OR (((TI handsearch* OR AB handsearch* OR SU handsearch*) OR (TI "hand search*" OR AB "hand search*" OR SU "hand search*"))) OR (((TI "mantel haenszel" OR AB "mantel haenszel" OR SU "mantel haenszel") OR (TI peto OR AB peto OR SU peto) OR (TI "der simonian" OR AB "der simonian" OR SU "der simonian") OR (TI dersimonian OR AB dersimonian OR SU dersimonian) OR (TI "fixed effect*" OR AB "fixed effect*" OR SU "fixed effect*") OR (TI "latin square*" OR AB "latin square*" OR SU "latin square*"))) OR (((TI "met analy*" OR AB "met analy*" OR SU "met analy*") OR (TI metanaly* OR AB metanaly* OR SU metanaly*) OR (TI "technology assessment*" OR AB "technology assessment*" OR SU "technology assessment*") OR (TI HTA OR AB HTA OR SU HTA) OR (TI HTAs OR AB HTAs OR SU HTAs) OR (TI "technology overview*" OR AB "technology overview*" OR SU "technology overview*") OR (TI "technology appraisal*" OR AB "technology appraisal*" OR SU "technology appraisal*"))) OR (((TI "meta regression*" OR AB "meta regression*" OR SU "meta regression*") OR (TI metaregression* OR AB metaregression* OR SU metaregression*))) OR ((meta-analy* OR metaanaly* OR "systematic review*" OR "biomedical technology assessment*" OR "bio-medical technology assessment*") ,hw.) OR (((TI medline OR AB medline) OR (TI cochrane OR AB cochrane) OR (TI pubmed OR AB pubmed) OR (TI medlars OR AB medlars) OR (TI embase OR AB embase) OR (TI cinahl OR AB cinahl)) ,hw.)

S5 ((cochrane OR (health N2 "technology assessment") OR "evidence report") .jw.) OR (((TI comparative OR AB comparative OR SU comparative) N3 ((TI efficacy OR AB efficacy OR SU efficacy) OR (TI effectiveness OR AB effectiveness OR SU effectiveness)))) OR (((TI "outcomes research" OR AB "outcomes research" OR SU "outcomes research") OR (TI "relative effectiveness" OR AB "relative effectiveness" OR SU "relative effectiveness"))) OR ((((TI indirect OR AB indirect OR SU indirect) OR (TI "indirect treatment" OR AB "indirect treatment" OR SU "indirect treatment") OR (TI mixed-treatment OR AB mixed-treatment OR SU mixed-treatment) OR (TI bayesian OR AB bayesian OR SU bayesian)) N3 (TI comparison* OR AB comparison* OR SU comparison*))) OR (((TI multi* OR AB multi* OR SU multi*) N3 (TI treatment OR AB treatment OR SU treatment) N3 (TI comparison* OR AB comparison* OR SU comparison*))) OR ((TI "umbrella review*" OR AB "umbrella review*" OR SU "umbrella review*")) OR (((TI multi* OR AB multi* OR SU multi*) N2 (TI paramet* OR AB paramet* OR SU paramet*) N2 (TI evidence OR AB evidence OR SU evidence) N2 (TI synthesis OR AB synthesis OR SU synthesis))) OR (((TI multiparamet* OR AB multiparamet* OR SU multiparamet*) N2 (TI evidence OR AB evidence OR SU evidence) N2 (TI synthesis OR AB synthesis OR SU synthesis))) OR (((TI multi-paramet* OR AB multi-paramet* OR SU multi-paramet*) N2 (TI evidence OR AB evidence OR SU evidence) N2 (TI synthesis OR AB synthesis OR SU synthesis))

S6 ((TI search: OR AB search:))

S7 PT review

S8 S4 OR S5 OR S6 OR S7

S9 S3 AND S8

S10 (TX validat*) or (TI index or model) or (AB index or model)

S11 TI (stratification or "ROC curve" or discriminat" or c-statistic" or "Area under the curve" or AUC or Calibration* or indices* or algorithm* or multivaria*) OR AB (stratification or "ROC curve" or discriminat" or c-statistic" or "Area under the curve" or AUC or Calibration* or indices* or algorithm* or multivaria*)

S12 (MH "ROC Curve")

S13 TI Validat* or Predict* or Rule* or (Predict* and (Outcome* or Risk* or Model*)) or ((History or Variable* or Criteria or Scor* or Characteristic* or Finding* or Factor*) and (Predict* or Model* or Decision* or Identif* or Prognos*)) or (Decision* and ((Model* or Clinical*) or (Prognostic and (History or Variable* or Criteria or Scor* or Characteristic* or Finding* or Factor* or Model*))

S14 AB Validat* or Predict* or Rule* or (Predict* and (Outcome* or Risk* or Model*)) or ((History or Variable* or Criteria or Scor* or Characteristic* or Finding* or Factor*) and (Predict* or Model* or Decision* or Identif* or Prognos*)) or (Decision* and ((Model* or Clinical*) or (Prognostic and (History or Variable* or Criteria or Scor* or Characteristic* or Finding* or Factor* or Model*))

S15 (MH "Models, Statistical+")

S16 S10 OR S11 OR S12 OR S13 OR S14 OR S15

S17 S9 AND S16

S18 (EM 20230101-20241212) OR (ZD "in process" AND RD 20230101-20241212)

S19 S17 AND S18

EPISTEMONIKOS

Date run: 31/01/23
Date update run: 21/06/24
For update, all searches limited by date added to database: From: 01/01/2023 To: 21/06/2024

Search 1

(title:(decubit* OR bedsore* OR bed-sore* OR pressure-ulcer* OR pressure-wound*) OR abstract:(decubit* OR bedsore* OR bed-sore* OR pressure-ulcer* OR pressure-wound*)) AND (title:(stratification OR "ROC curve" OR "ROC curves" OR "receiver operating characteristic" OR discriminat* OR c-statistic OR "Area under the curve" OR AUC OR Calibration OR indices OR algorithm OR multivaria* OR Validat* OR Predict* OR Rule* OR Risk* OR Model* OR Criteria OR Scor* OR Characteristic* OR Finding* OR Factor* OR Decision* OR Prognos* OR Index OR model OR prevent*) OR abstract:(stratification OR "ROC curve" OR "ROC curves" OR "receiver operating characteristic" OR discriminat* OR c-statistic OR "Area under the curve" OR AUC OR Calibration OR indices OR algorithm OR multivaria* OR Validat* OR Predict* OR Rule* OR Risk* OR Model* OR Criteria OR Scor* OR Characteristic* OR Finding* OR Factor* OR Decision* OR Prognos* OR Index OR model OR prevent*))
Limit by publication type: systematic review (106) (Update: 21) or broad synthesis (3) (Update: 0)

Search 2

(title:("pressure ulcer" OR "pressure ulcers" OR "pressure sore" OR "pressure sores" OR "pressure lesion" OR "pressure lesions" OR "pressure injury" OR "pressure injuries") OR abstract:("pressure

ulcer" OR "pressure ulcers" OR "pressure sore" OR "pressure sores" OR "pressure lesion" OR "pressure lesions" OR "pressure injury" OR "pressure injuries")) AND (title:(stratification OR "ROC curve" OR "ROC curves" OR "receiver operating characteristic" OR discriminat* OR c-statistic OR "Area under the curve" OR AUC OR Calibration OR indices OR algorithm OR multivaria* OR Validat* OR Predict* OR Rule* OR Risk* OR Model* OR Criteria OR Scor* OR Characteristic* OR Finding* OR Factor* OR Decision* OR Prognos* OR Index OR model OR prevent*) OR abstract:(stratification OR "ROC curve" OR "ROC curves" OR "receiver operating characteristic" OR discriminat* OR c-statistic OR "Area under the curve" OR AUC OR Calibration OR indices OR algorithm OR multivaria* OR Validat* OR Predict* OR Rule* OR Risk* OR Model* OR Criteria OR Scor* OR Characteristic* OR Finding* OR Factor* OR Decision* OR Prognos* OR Index OR model OR prevent*))
Limit by publication type: systematic review (709) (Update: 129) or broad synthesis (35) (Update: 25)

Search 3

(title:("deep-tissue wound" OR "deep-tissue wounds" OR "deep-tissue ulcer" OR "deep-tissue ulcers" OR "deep-tissue sore" OR "deep-tissue sores" OR "deep-tissue lesion" OR "deep-tissue lesions" OR "deep-tissue injury" OR "deep-tissue injuries") OR abstract:("deep-tissue wound" OR "deep-tissue wounds" OR "deep-tissue ulcer" OR "deep-tissue ulcers" OR "deep-tissue sore" OR "deep-tissue sores" OR "deep-tissue lesion" OR "deep-tissue lesions" OR "deep-tissue injury" OR "deep-tissue injuries")) AND (title:(stratification OR "ROC curve" OR "ROC curves" OR "receiver operating characteristic" OR discriminat* OR c-statistic OR "Area under the curve" OR AUC OR Calibration OR indices OR algorithm OR multivaria* OR Validat* OR Predict* OR Rule* OR Risk* OR Model* OR Criteria OR Scor* OR Characteristic* OR Finding* OR Factor* OR Decision* OR Prognos* OR Index OR model OR prevent*) OR abstract:(stratification OR "ROC curve" OR "ROC curves" OR "receiver operating characteristic" OR discriminat* OR c-statistic OR "Area under the curve" OR AUC OR Calibration OR indices OR algorithm OR multivaria* OR Validat* OR Predict* OR Rule* OR Risk* OR Model* OR Criteria OR Scor* OR Characteristic* OR Finding* OR Factor* OR Decision* OR Prognos* OR Index OR model OR prevent*))
Limit by publication type: systematic review (0) (Update: 0) or broad synthesis (0) (Update: 0)

Search 4

(title:("deep tissue wound" OR "deep tissue wounds" OR "deep tissue ulcer" OR "deep tissue ulcers" OR "deep tissue sore" OR "deep tissue sores" OR "deep tissue lesion" OR "deep tissue lesions" OR "deep tissue injury" OR "deep tissue injuries") OR abstract:("deep tissue wound" OR "deep tissue wounds" OR "deep tissue ulcer" OR "deep tissue ulcers" OR "deep tissue sore" OR "deep tissue sores" OR "deep tissue lesion" OR "deep tissue lesions" OR "deep tissue injury" OR "deep tissue injuries")) AND (title:(stratification OR "ROC curve" OR "ROC curves" OR "receiver operating characteristic" OR discriminat* OR c-statistic OR "Area under the curve" OR AUC OR Calibration OR indices OR algorithm OR multivaria* OR Validat* OR Predict* OR Rule* OR Risk* OR Model* OR Criteria OR Scor* OR Characteristic* OR Finding* OR Factor* OR Decision* OR Prognos* OR Index OR model OR prevent*) OR abstract:(stratification OR "ROC curve" OR "ROC curves" OR "receiver operating characteristic" OR discriminat* OR c-statistic OR "Area under the curve" OR AUC OR Calibration OR indices OR algorithm OR multivaria* OR Validat* OR Predict* OR Rule* OR Risk* OR Model* OR Criteria OR Scor* OR Characteristic* OR Finding* OR Factor* OR Decision* OR Prognos* OR Index OR model OR prevent*))
Limit by publication type: systematic review (5) (Update: 1) or broad synthesis (2) (Update: 0)

Search 5

(title:("bed wound" OR "bed wounds" OR "bed ulcer" OR "bed ulcers" OR "bed sore" OR "bed sores" OR "bed lesion" OR "bed lesions" OR "bed injury" OR "bed injuries") OR abstract:("bed wound" OR "bed wounds" OR "bed ulcer" OR "bed ulcers" OR "bed sore" OR "bed sores" OR "bed lesion" OR "bed lesions" OR "bed injury" OR "bed injuries")) AND (title:(stratification OR "ROC curve" OR "ROC curves" OR "receiver operating characteristic" OR discriminat* OR c-statistic OR "Area under the curve" OR AUC OR Calibration OR indices OR algorithm OR multivaria* OR Validat* OR Predict* OR Rule* OR Risk* OR Model* OR Criteria OR Scor* OR Characteristic* OR Finding* OR Factor* OR Decision* OR Prognos* OR Index OR model OR prevent*) OR abstract:(stratification OR "ROC curve" OR "ROC curves" OR "receiver operating characteristic" OR discriminat* OR c-statistic OR "Area under the curve" OR AUC OR Calibration OR indices OR algorithm OR multivaria* OR Validat* OR Predict* OR Rule* OR Risk* OR Model* OR Criteria OR Scor* OR Characteristic* OR Finding* OR Factor* OR Decision* OR Prognos* OR Index OR model OR prevent*))
Limit by publication type: systematic review (14) (Update: 0) or broad synthesis (0) (Update: 1)

Search 6

(title:("bed bound" OR bed-bound OR bedridden OR bed-ridden OR "bed ridden") OR abstract:("bed bound" OR bed-bound OR bedridden OR bed-ridden OR "bed ridden")) AND (title:(stratification OR "ROC curve" OR "ROC curves" OR "receiver operating characteristic" OR discriminat* OR c-statistic OR "Area under the curve" OR AUC OR Calibration OR indices OR algorithm OR multivaria* OR Validat* OR Predict* OR Rule* OR Risk* OR Model* OR Criteria OR Scor* OR Characteristic* OR Finding* OR Factor* OR Decision* OR Prognos* OR Index OR model OR prevent*) OR abstract:(stratification OR "ROC curve" OR "ROC curves" OR "receiver operating characteristic" OR discriminat* OR c-statistic OR "Area under the curve" OR AUC OR Calibration OR indices OR algorithm OR multivaria* OR Validat* OR Predict* OR Rule* OR Risk* OR Model* OR Criteria OR Scor* OR Characteristic* OR Finding* OR Factor* OR Decision* OR Prognos* OR Index OR model OR prevent*))
Limit by publication type: systematic review (30) (Update: 3) or broad synthesis (1) (Update: 1)

Search 7

(title:(stratification OR "ROC curve" OR "ROC curves" OR "receiver operating characteristic" OR discriminat* OR c-statistic OR "Area under the curve" OR AUC OR Calibration OR indices OR algorithm OR multivaria* OR Validat* OR Predict* OR Rule* OR Risk* OR Model* OR Criteria OR Scor* OR Characteristic* OR Finding* OR Factor* OR Decision* OR Prognos* OR Index OR model OR prevent*) OR abstract:(stratification OR "ROC curve" OR "ROC curves" OR "receiver operating characteristic" OR discriminat* OR c-statistic OR "Area under the curve" OR AUC OR Calibration OR indices OR algorithm OR multivaria* OR Validat* OR Predict* OR Rule* OR Risk* OR Model* OR Criteria OR Scor* OR Characteristic* OR Finding* OR Factor* OR Decision* OR Prognos* OR Index OR model OR prevent*)) AND (title:(wound* OR ulcer* OR sore* OR injur* OR lesion*) OR abstract:(wound* OR ulcer* OR sore* OR injur* OR lesion*)) AND (title:(supine OR immobil*) OR abstract:(supine OR immobil*))
Limit by publication type: systematic review (234) (Update: 39) or broad synthesis (13) (Update: 3)

Search 8

(title:(stratification OR "ROC curve" OR "ROC curves" OR "receiver operating characteristic" OR discriminat* OR c-statistic OR "Area under the curve" OR AUC OR Calibration OR indices OR algorithm OR multivaria* OR Validat* OR Predict* OR Rule* OR Risk* OR Model* OR Criteria OR

Scor* OR Characteristic* OR Finding* OR Factor* OR Decision* OR Prognos* OR Index OR model OR prevent*) OR abstract:(stratification OR "ROC curve" OR "ROC curves" OR "receiver operating characteristic" OR discriminat* OR c-statistic OR "Area under the curve" OR AUC OR Calibration OR indices OR algorithm OR multivaria* OR Validat* OR Predict* OR Rule* OR Risk* OR Model* OR Criteria OR Scor* OR Characteristic* OR Finding* OR Factor* OR Decision* OR Prognos* OR Index OR model OR prevent*)) AND (title:(heal OR healing OR heals OR healed OR dress*) OR abstract:(heal OR healing OR heals OR healed OR dress*)) AND (title:(supine OR immobil*) OR abstract:(supine OR immobil*))
Limit by publication type: systematic review (39) (Update: 9) or broad synthesis (3) (Update: 0)

GOOGLE SCHOLAR 1/02/23
Update run: 24/06/24, limit to years 2023-2024

allintitle: prevent OR prevention OR risk OR predict OR prevents OR risks OR prediction OR predicts OR prognosis OR prognostic "pressure injury" -ulcer -ulcers Limit to review and years 2013-2023 (66) (Update: 31)

allintitle: prevent OR prevention OR risk OR predict OR prevents OR risks OR prediction OR predicts OR prognosis OR prognostic "pressure injuries" -ulcer -ulcers Limit to review and years 2013-2023 (33)
(Update: 14)

allintitle: prevent OR prevention OR risk OR predict OR prevents OR risks OR prediction OR predicts OR prognosis OR prognostic "pressure ulcer" Limit to review and years 2013-2023 (120)
(Update: 14)

allintitle: prevent OR prevention OR risk OR predict OR prevents OR risks OR prediction OR predicts OR prognosis OR prognostic "pressure ulcers" Limit to review and years 2013-2023 (102)
(Update: 14)

allintitle: prevent OR prevention OR risk OR predict OR prevents OR risks OR prediction OR predicts OR prognosis OR prognostic "pressure sore" Limit to review and years 2013-2023 (3)
(Update: 0)

allintitle: prevent OR prevention OR risk OR predict OR prevents OR risks OR prediction OR predicts OR prognosis OR prognostic "pressure sores" Limit to review and years 2013-2023 (2)
(Update: 0)

allintitle: prevent OR prevention OR risk OR predict OR prevents OR risks OR prediction OR predicts OR prognosis OR prognostic "pressure wound" Limit to review and years 2013-2023 (25)
(Update: 4)

allintitle: prevent OR prevention OR risk OR predict OR prevents OR risks OR prediction OR predicts OR prognosis OR prognostic "pressure wounds" Limit to review and years 2013-2023 (0)
(Update: 0)

allintitle: prevent OR prevention OR risk OR predict OR prevents OR risks OR prediction OR predicts OR prognosis OR prognostic "bedsore" Limit to review and years 2013-2023 (1)
(Update: 0)

allintitle: prevent OR prevention OR risk OR predict OR prevents OR risks OR prediction OR predicts OR prognosis OR prognostic "bedsores” Limit to review and years 2013-2023 (1)
(Update: 1)

allintitle: prevent OR prevention OR risk OR predict OR prevents OR risks OR prediction OR predicts OR prognosis OR prognostic "bed sore” Limit to review and years 2013-2023 (0)
(Update: 0)

allintitle: prevent OR prevention OR risk OR predict OR prevents OR risks OR prediction OR predicts OR prognosis OR prognostic "bed sores” Limit to review and years 2013-2023 (0)
(Update: 0)

allintitle: prevent OR prevention OR risk OR predict OR prevents OR risks OR prediction OR predicts OR prognosis OR prognostic "decubitus” Limit to review and years 2013-2023 (4)
(Update: 2)

Appendix C: Data extraction form

Data Extraction Items	
	Extractor
**Publication information:**	Review Title;<br>First Author;<br>Publication Year;<br>Umbrella review eligibility (DTA, CE);<br>Comments;<br>Primary studies fundings reported?[A];<br>Conflicts of Interest reported?[A]
**Eligibility Criteria:**	Population;<br>Setting;<br>Prediction models/tools;<br>Model outcome (and classification if specified);<br>Interventions[B];<br>Comparators[B];<br>Outcomes of interest[B];<br>Inclusion criteria incorporated PICO, PIRT or POII?[A];<br>Source of data (prospective/retrospective);<br>Phase of development of models;<br>Study design;<br>Did they explain reasons for study design inclusions?[A];<br>Exclusion criteria
**Review methods:**	Review protocol;<br>Protocol and justifications for deviations from?[A];<br>Databases searched;<br>Adequate search strategy?[A];<br>Search cut-off date;<br>Publication restrictions;<br>Quality assessment tool;<br>Suitable quality assessment tool?;<br>Study selection method;<br>Study selection in duplicate?[A];<br>Quality assessment method;<br>Data extraction method;<br>Data extraction in duplicate?[A];<br>Synthesis method;<br>Appropriate method of statistical synthesis, if applicable?[A]
**Review results:**	PRISMA diagram provided?;<br>Excluded studies list (with justifications)?[A];<br>N models per review;<br>N studies per review;<br>N participants in review;<br>How were the results presented? (e.g. outcomes reported);<br>Description of included studies provided? (summary table, tabulated per study, narrative only);<br>Description of included studies adequate?[A];<br>Study quality described? (summary table, tabulated per study, narrative only);<br>Assessment of RoB satisfactory?[A];<br>Assessment of impact of RoB on synthesised results?[A];<br>Assessment of impact of RoB on review results?[A];<br>Discussion/investigation of heterogeneity?[A];<br>Models included;<br>Brief description of included studies;<br>Brief description of study quality

Reviews on model accuracy	Reviews on clinical effectiveness
2x2 tables presented for each study?	
Cut-off points specified for each study?	
List Author, year of primary studies included in review	
Summary estimates:<br>Sensitivity (incl. n), specificity (incl. N), likelihood ratios, DOR, AUROC, predictive values<br>Summary Sensitivity (incl. n)<br>(results from statistical synthesis)	Summary of statistical synthesis of results<br>(e.g. effect on incidence of PI, treatment outcome, or other patient-relevant outcomes)
Summary of narrative synthesis of results	Summary of narrative synthesis of results

DTA – diagnostic test accuracy; CE – clinical effectiveness; PICO – population, intervention, comparator, outcome; PIRT – population, index test, reference standard, target condition; POII – population, outcome, intended use, intended timing; PRISMA – Preferred Reporting Items for Systematic Reviews and Meta-Analyses; RoB – risk of bias; O/E – observed/expected; AUC – area under the curve; DOR – diagnostic odds ratio; AUROC – area under the receiver operating characteristic curve; PI – pressure injury; AMSTAR – A MeaSurement Tool to Assess systematic Reviews

[A] AMSTAR-2 Items.

[B] applicable to clinical effectiveness reviews only.

Appendix D: AMSTAR-2 Methodology Quality Appraisal. Adapted for application to reviews of prognostic model and accuracy studies.

	AMSTAR-2 Adapted	
	Questions	Guidance
Item 1.	1. Did the research questions and inclusion criteria for the review include the components one of the following: PICO, or PIRT and POII?<br>**Y/N**	For intervention reviews:<br>Population, Intervention, Comparator, Outcome (PICO)<br><br>For prognostic accuracy reviews:<br>Population, Index test, Reference standard, Target condition (PIRT)<br>Population, Outcome to be predicted, Intended use of model, Intended moment in time (POII)
**Item 2***	2. Did the report of the review contain an explicit statement that the review methods were established prior to the conduct of the review and did the report justify any significant deviations from the protocol?<br>**Y/PY/N**	For Partial Yes (PY):<br>The authors state that they had a written protocol or guide that included ALL the following:<br>• review question(s),<br>• a search strategy,<br>• inclusion/exclusion criteria,<br>• a risk of bias assessment.<br>For Yes:<br>As for partial yes, plus the protocol should be registered and should also have specified:<br>• a meta-analysis/synthesis plan, if appropriate,<br>• and a plan for investigating causes of heterogeneity,<br>• justification for any deviations from the protocol.

Item 3.	3. Did the review authors explain their selection of the study designs for inclusion in the review?<br>**Y/N**	For Yes, the review should give an explanation for including types of studies included in the review, for example:<br><br>For the DEV/VAL review: development studies, validation studies or both.<br>For the accuracy/effectiveness review: single group (prospective/retrospective), two/multi group (i.e diagnostic case-control), RCTs, NSRs
**Item 4***	4. Did the review authors use a comprehensive literature search strategy?<br>**Y/PY/N**	For Partial Yes (all the following):<br>• searched at least 2 databases (relevant to research question),<br>• provided key word and/or search strategy,<br>• justified publication restrictions (e.g. language).<br>For Yes, should also have (all the following):<br>• searched the reference lists / bibliographies of included studies,<br>• searched trial/study registries,<br>• included/consulted content experts in the field where relevant,<br>• searched for grey literature,<br>• conducted search within 24 months of completion of the review.
Item 5.	5. Did the review authors perform study selection in duplicate?<br>**Y/N**	For Yes, either ONE of the following:<br>at least two reviewers independently agreed on selection of eligible studies and achieved consensus on which studies to include,<br>OR two reviewers selected a sample of eligible studies and achieved good agreement (at least 80 percent), with the remainder selected by one reviewer.
Item 6.	6. Did the review authors perform data extraction in duplicate?<br>**Y/N**	For Yes, either ONE of the following:<br>at least two reviewers achieved consensus on which data to extract from included studies,<br>OR two reviewers extracted data from a sample of eligible studies and achieved good agreement (at least 80 percent), with the remainder extracted by one reviewer.

**Item 7***	7. Did the review authors provide a list of excluded studies and justify the exclusions?<br>**Y/PY/N**	For Partial Yes:<br>provided a list of all potentially relevant studies that were read in full-text form but excluded from the review<br><br>For Yes, must also have:<br>Justified the exclusion from the review of each potentially relevant study
Item 8.	8. Did the review authors describe the included studies in adequate detail?<br>**Y/PY/N**	For Partial Yes (ALL the following per included study):<br>• described PICO/PIRT/POII (whichever applicable),<br>• and described research designs<br>For Yes, should also have ALL the following per included study:<br>• described PICO/PIRT/POII (whichever applicable) in detail,<br>• described study's setting<br>• and timeframe for follow-up
**Item 9***	9. Did the review authors use a satisfactory technique for assessing the risk of bias (RoB) in individual studies that were included in the review?<br>**Y/PY/N**	**RCTs**<br>For Partial Yes, must have reported summary findings and assessed RoB from:<br>• unconcealed allocation,<br>• and lack of blinding of patients and assessors when assessing outcomes (unnecessary for objective outcomes such as all-cause mortality)<br>For Yes, must also have given itemisation of quality judgements per study, and assessed RoB from:<br>• allocation sequence that was not truly random,<br>• and selection of the reported result from among multiple measurements or analyses of a specified outcome
		**NRS**<br>For Partial Yes, must have reported summary findings and assessed RoB from:<br>• confounding,<br>• and from selection bias<br>For Yes, must also have given itemisation of quality judgements per study, and assessed RoB from:<br>• methods used to ascertain exposures and outcomes,<br>• and selection of the reported result from among multiple measurements or analyses of a specified outcome

		**DTA studies**<br>For Partial Yes, must have assessed RoB with a recognised tool (e.g. QUADAS-2) and given summary of result across domains<br><br>For Yes, must also have also given itemisation of quality judgements per study.
		**Prognostic studies**<br>For Partial Yes, must have assessed RoB with a recognised tool (e.g. PROBAST, QUIPS) and given summary of result across domains<br><br>For Yes, must also have also given itemisation of quality judgements per study.
Item 10.	10. Did the review authors report on the sources of funding for the studies included in the review?<br>**Y/N**	For Yes:<br>Must have reported on the sources of funding for individual studies included in the review. Note: Reporting that the reviewers looked for this information, but it was not reported by study authors also qualifies
**Item 11***	11. If meta-analysis was performed did the review authors use appropriate methods for statistical combination of results?<br>**Y/N/ 'No MA conducted'**	For Yes:<br>The authors justified combining the data in a meta-analysis<br>AND they used an appropriate weighted technique to combine study results and adjusted for heterogeneity if present.<br>AND investigated the causes of any heterogeneity
Item 12.	12. If meta-analysis was performed, did the review authors assess the potential impact of RoB in individual studies on the results of the meta-analysis or other evidence synthesis?<br>**Y/N/ 'No MA conducted'**	For Yes:<br>included only low risk of bias studies<br>OR, if the pooled estimate was based on studies at variable RoB, the authors performed sensitivity analyses to investigate possible impact of RoB on summary estimates
**Item 13***	13. Did the review authors account for RoB in individual studies when interpreting/ discussing the results of the review?<br>**Y/N**	For Yes:<br>included only low risk of bias RCTs<br>OR, if RCTs with moderate or high RoB, or NRSs were included the review provided a discussion of the likely impact of RoB on the results

Item 14.	14. Did the review authors provide a satisfactory explanation for, and discussion of, any heterogeneity observed in the results of the review?<br>**Y/N**	For Yes:<br>There was no significant heterogeneity,<br>OR if heterogeneity was present, the authors performed an investigation of main sources of heterogeneity in the results, if applicable, and particularly any between-study heterogeneity and discussed the impact of this
Item 15.	15. Did the review authors report any potential sources of conflict of interest, including any funding they received for conducting the review?<br>**Y/N**	For Yes:<br>The authors reported no competing interests,<br>OR the authors described their funding sources and how they managed potential conflicts of interest

* Critical domains identified by AMSTAR-2 developers.[1]

PICO – population, intervention, comparator, outcome; PIRT – population, index test, reference standard, target condition; POII – population, outcome, intended use, intended time; DEV/VAL – development/validation; RCT – randomised controlled trial; NRS – non-randomised study; QUADAS – Quality Assessment of Diagnostic Accuracy Studies; PROBAST – Prediction model Risk Of Bias ASsessment Tool; QUIPS – Quality In Prognosis Studies; MA – Meta-Analysis

Appendix E: Detailed results tables

Table A. Full-text articles excluded, with reasons

	Author, year	Title	Major reason for exclusion
1	Alves, 2014 [2]	*Assessment of risk for pressure ulcers in intensive care units: an integrative review*	Not a systematic review
2	Anthony, 2008 [3]	*Norton, Waterlow and Braden scores: a review of the literature and a comparison between the scores and clinical judgement*	Not a systematic review
3	Barradas Cavalcante, 2016 [4]	*Updating pf the assistance protocol for pressure prevention: evidence based practice*	Not a systematic review
4	Charalambous, 2018 [5]	*Evaluation of the Validity and Reliability of the Waterlow Pressure Ulcer Risk Assessment Scale*	Not a systematic review
5	de Laat, 2006 [6]	*Epidemiology, risk and prevention of pressure ulcers in critically ill patients: a literature review*	Not a systematic review
6	do Egito Cavalcanti de Farias, 2022 [7]	*Risk factors for the development of pressure injury in the elderly: integrative review*	Not a systematic review
7	Feuchtinger, 2005 [8]	*Pressure ulcer risk factors in cardiac surgery: A review of the research literature*	Not a systematic review
8	Garcia-Fernandez, 2014 [9]	*A new theoretical model for the development of pressure ulcers and other dependence-related lesions*	Not a systematic review
9	Garrubba, 2017 [10]	*Effectiveness of the Braden risk screening tool for pressure injuries: systematic review*	Not a systematic review
10	Kelechi, 2013 [11]	*Review of pressure ulcer risk assessment scales*	Not a systematic review
11	Keller, 2002 [12]	*Pressure ulcers in intensive care patients: A review of risks and prevention*	Not a systematic review
12	Ladd, 2018 [13]	*A systematic review of pressure ulcers in burn patients: Risk factors, demographics, and treatment modalities*	Not a systematic review
13	Lepisto, 2006 [14]	*Developing a Pressure Ulcer Risk Assessment Scale for Patients in Long-Term Care*	Not a systematic review
14	Mendes Coqueiro, 2013 [15]	*Multiple risk factors and preventive strategies of pressure ulcers: systematic review*	Not a systematic review
15	Michel, 2012 [16]	*As of 2012, what are the key predictive risk factors for pressure ulcers? Developing French guidelines for clinical practice*	Not a systematic review
16	Ming, 2012 [17]	*Systematic review of pressure ulcer risk assessment scales for using in ICU patients*	Not a systematic review
17	Mordiffi, 2010 [18]	*Evaluating the effects of using the mobility assessment sub-scale within the Braden Scale on pressure ulcer incidence and preventive interventions in adult acute care settings: A systematic review*	Not a systematic review
18	Mortenson, 2008 [19]	*A review of scales for assessing the risk of developing a pressure ulcer in individuals with SCI*	Not a systematic review
19	Nadeem, 2021 [20]	*Utility of the Waterlow scale in acute care settings: A literature review*	Not a systematic review
20	O'Tuathail, 2011 [21]	*Evaluation of three commonly used pressure ulcer risk assessment scales*	Not a systematic review
21	Rodriguez Tores, 2007 [22]	*Clinical judgement or assessment scales to identify patients at risk of developing pressure ulcers?*	Not a systematic review
22	Sales de Almeida, 2020 [23]	*Pressure injury prevention scales in intensive care units: an integrative review*	Not a systematic review
23	Santos, 2015 [24]	*Development of the nursing diagnosis risk for pressure ulcer*	Not a systematic review
24	Satekova, 2014 [25]	*Validity of pressure ulcer risk assesment scales: Review*	Not a systematic review
25	Shahin, 2007 [26]	*Predictive validity of pressure ulcer risk assessment tools in intensive care patients*	Not a systematic review
26	Smet, 2019 [27]	*The Belgian pressure ulcer risk assessment project: Is assessing mobility and skin status a more accurate, reliable, and feasible approach to assess pressure ulcer risk in hospitalised patients?*	Not a systematic review

27	Solati, 2016 [28]	*Predictive values of Braden and Waterlow scales to assess the risk of pressure ulcer*	Not a systematic review
28	Taylor, 1988 [29]	*Assessment tools for the identification of patients at risk for the development of pressure sores: a review*	Not a systematic review
29	Tran, 2016 [30]	*Prevention of Pressure Ulcers in the Acute Care Setting: New Innovations and Technologies*	Not a systematic review
30	Tschannen, 2020 [31]	*The pressure injury predictive model: A framework for hospital-acquired pressure injuries*	Not a systematic review
31	Walsh, 2011 [32]	*Investigating the reliability and validity of the Waterlow risk assessment scale: A literature review*	Not a systematic review
32	Xu, 2018 [33]	*Risk assessment tools for pressure injury in intensive care patients: a review*	Not a systematic review
33	Alderden, 2017 [34]	*Risk factors for pressure injuries among critical care patients: A systematic review*	No risk prediction models
34	Barbosa da Silva, 2020 [35]	*Pressure ulcers in individuals with spinal cord injury: risk factors in neurological rehabilitation*	No risk prediction models
35	Di Prinzio, 2019 [36]	*Risk factors for the development and recurrence of pressure ulcers in patients with spinal cord injury: A systematic review*	No risk prediction models
36	Haisley, 2020 [37]	*Postoperative pressure injuries in adults having surgery under general anaesthesia: systematic review of perioperative risk factors*	No risk prediction models
37	Ham, 2014 [38]	*Pressure ulcers from spinal immobilization in trauma patients: A systematic review*	No risk prediction models
38	Lima, 2021 [39]	*Risk factors and preventive interventions for pressure injuries in cancer patients*	No risk prediction models
39	Lima Serrano, 2017 [40]	*Risk factors for pressure ulcer development in Intensive Care Units: Systematic review*	No risk prediction models
40	Marin, 2013 [41]	*A systematic review of risk factors for the development and recurrence of pressure ulcers in people with spinal cord injuries*	No risk prediction models
41	Rao, 2016 [42]	*Risk Factors Associated With Pressure Ulcer Formation in Critically Ill Cardiac Surgery Patients: A Systematic Review*	No risk prediction models
42	Reenalda, 2009 [43]	*Clinical use of interface pressure to predict pressure ulcer development: a systematic review*	No risk prediction models
43	Shi, 2018 [44]	*Skin status for predicting pressure ulcer development: A systematic review and meta-analyses*	No risk prediction models
44	Siping, 2022 [45]	*Risk factors of intraoperative acquired pressure injury: A systematic review and meta-analysis*	No risk prediction models
45	Wynn, 2022 [46]	*Risk factors for the development and evolution of deep tissue injuries: A systematic review*	No risk prediction models
46	Zhang, 2022 [47]	*Prevalence and Risk Factors of Postoperative Pressure Ulcers: A Systematic Review and Meta-analysis of Diagnostic Test*	No risk prediction models
47	Bulfone, 2018 [48]	*Perioperative Pressure Injuries: A Systematic Literature Review*	Wrong research question
48	Chung, 2022 [49]	*Risk Factors for Pressure Injuries in Adult Patients: A Narrative Synthesis*	Wrong research question
49	Chung, 2022 [50]	*Risk factors for pressure ulcers in adult patients: A meta-analysis on sociodemographic factors and the Braden scale*	Wrong research question
50	Coleman, 2013 [51]	*Patient risk factors for pressure ulcer development: Systematic review*	Wrong research question
51	Dube, 2022 [52]	*Risk factors associated with heel pressure ulcer development in adult population: A systematic literature review*	Wrong research question
52	Ferris, 2019 [53]	*Pressure ulcers in patients receiving palliative care: A systematic review*	Wrong research question
53	Floyd, 2018 [54]	*Effectiveness of pressure ulcer protocols with the Braden Scale for elderly patients in the intensive care unit: A Systematic Review*	Wrong research question
54	Gelis, 2009 [55]	*Pressure ulcer risk factors in persons with SCI: Part I: Acute and rehabilitation stages*	Wrong research question
55	Gelis, 2009 [56]	*Pressure ulcer risk factors in persons with spinal cord injury part 2: the chronic stage*	Wrong research question

56	Liu, 2024 [57]	*Effects of predictive nursing interventions on pressure ulcer in older bedridden patients: A meta-analysis*	Wrong research question
57	Moore, 2023 [58]	*A systematic review of movement monitoring devices to aid the prediction of pressure ulcers in at-risk adults*	Wrong research question
58	Mordiffi, 2011 [59]	*Use of mobility subscale for risk assessment of pressure ulcer incidence and preventive interventions: A systematic review*	Wrong research question
59	Nixon, 2015 [60]	*Pressure UlceR Programme Of reSEarch (PURPOSE): using mixed methods (systematic reviews, prospective cohort, case study, consensus and psychometrics) to identify patient and organisational risk, develop a risk assessment tool and patient-reported outcome Quality of Life and Health Utility measures*	Wrong research question
60	Richardson, 2015 [61]	*Part 1: Pressure ulcer assessment - the development of Critical Care Pressure Ulcer Assessment Tool made Easy (CALCULATE)*	Wrong research question
61	Teixeira, 2022 [62]	*Risk factors for pressure injury in critically ill polytraumatized patients: A systematic review*	Wrong research question
62	Ting, 2021 [63]	*E-Health Decision Support Technologies in the Prevention and Management of Pressure Ulcers: A Systematic Review*	Wrong research question
63	Toffaha, 2023 [64]	*Leveraging artificial intelligence and decision support systems in hospital-acquired pressure injuries prediction: A comprehensive review*	Wrong research question
64	Fuentelsaz Gallego, 2005 [65]	*Review of literature on pressure ulcers in people aged 65 or over*	No English language translation
65	Garcia-Fernandez, 2013 [66]	*Risk assessment scales for pressure ulcer in intensive care units: A systematic review with metaanalysis*	No English language translation
66	Kottner, 2008 [67]	*Interrater reliability of the Braden scale*	No English language translation
67	Nunes de Sousa, 2023 [68]	*SCALES USED TO MEASURE PRESSURE INJURY RISK IN HOSPITALIZED PATIENTS: A REVIEW*	No English language translation
68	Pancorbo-Hidalgo, 2008 [69]	*Pressure ulcers risk assessment: clinical practice in Spain and a meta-analysis of scales effectiveness*	No English language translation
69	Park, 2014 [70]	*Predictive validity of the Braden Scale for pressure ulcer risk: a meta-analysis*	No English language translation
70	Yang, 2019 [71]	*Predictive validity of the Munro Scale for pressure injuries in surgical patients: A meta-analysis*	No English language translation
71	Barghouthi, 2023 [72]	*Systematic Review for Risks of Pressure Injury and Prediction Models Using Machine Learning Algorithms*	Reports model development or validation only
72	Dweekat, 2023 [73]	*Machine Learning Techniques, Applications, and Potential Future Opportunities in Pressure Injuries (Bedsores) Management: A Systematic Review*	Reports model development or validation only
73	Jiang, 2021 [74]	*Using Machine Learning Technologies in Pressure Injury Management: Systematic Review*	Reports model development or validation only
74	Ribeiro, 2021 [75]	*Literature review of machine-learning algorithms for pressure ulcer prevention: Challenges and opportunities*	Reports model development or validation only
75	Shi, 2019 [76]	*Evaluating the development and validation of empirically-derived prognostic models for pressure ulcer risk assessment: A systematic review*	Reports model development or validation only
76	Zhou, 2022 [77]	*A systematic review of predictive models for hospital-acquired pressure injury using machine learning*	Reports model development or validation only
77	De Queiroz, 2022 [7]	*Risk factors for the development of pressure injury in the elderly: integrative review/Fatores de risco o para desenvolvimento de lesão por pressão em idosos: revisão integrativa*	Duplicate
78	Garcia-Fernandez, 2013 [66]	*Risk assessment scales for pressure ulcers in intensive care units: A systematic review with meta-analysis*	Duplicate
79	Nixon, 2015 [60]	*Pressure UlceR Programme Of reSEarch (PURPOSE): using mixed methods (systematic reviews, prospective cohort, case study, consensus and psychometrics) to identify patient and*	Duplicate

		organisational risk, develop a risk assessment tool and patient-reported outcome Quality of Life and Health Utility measures	
80	Nayar, 2021 [78]	*Waterlow score for risk assessment in surgical patients: a systematic review*	Wrong outcome
81	Zahia, 2020 [79]	*Pressure injury image analysis with machine learning techniques: A systematic review on previous and possible future methods*	Wrong outcome
82	Moore, 2008 [80]	*Risk assessment tools for the prevention of pressure ulcers*	Updated version included
83	Moore, 2014 [81]	*Risk assessment tools for the prevention of pressure ulcers*	Updated version included
84	Liao, 2018 [82]	*Predictive accuracy of the Braden Q Scale in risk assessment for paediatric pressure ulcer: A meta-analysis*	Wrong population
85	Ribeiro, 2013 [83]	*How effective is the development of skin care in critically ill patients using the Braden Scale scores aiming to prevent the incidence of pressure ulcers? Sistematic Literature Review*	No results

Table B. Systematic review characteristics

Review author (publication year)<br><br>Review question	Eligibility criteria			Review methods				Volume of evidence	
	**Population; setting**	**Prediction tools; PI classification system**	**Study design**	**Databases searched**	**Publication restrictions** (year; language; publication type)	**Quality assessment tool**	**Meta-analysis included; method of meta-analysis**	**N relevant studies in review** (n participants)	**N tools included**
Baris[84] (2015)<br><br>Effectiveness	Turkish populations only; NS	Braden; NS	NS	Turkish MEDLINE; PubMed; ScienceDirect; Google Scholar; YOK Thesis Search; Reference Directory of Turkey; Medicine Directory of Turkish Clinics; ULAKBIM National Database; National Library Bibliography of Turkish Articles	1998-2012; English, Turkish; NS	None	No	16 (2273[A])	2
Chen[85] (2023)<br><br>Accuracy	Patients with a critical illness; ICU	Cubbin & Jackson; NS	Diagnostic studies (presenting TP, FP, TN and FN results) with any research design	EBSCO, PubMed, Ovid, Web of Science and Cochrane databases, Wangfang Data and China National Knowledge Infrastructure	Database inception - 2021; NS; reviews and expert opinions excluded	QUADAS-II	Yes; meta-analysis method unclear, SROC analysis	9 (7684)	1
Chen[86] (2016)<br><br>Accuracy	NS; long-term care	Braden; NS	NS	PubMed; Web of Science	Inception-2015; English; NS	QUADAS	Yes; DerSimonian and Laird random-effects model, SROC analysis	8 (41489)	1

Review author (publication year)<br><br>Review question	Eligibility criteria			Review methods				Volume of evidence	
	**Population; setting**	**Prediction tools; PI classification system**	**Study design**	**Databases searched**	**Publication restrictions** (year; language; publication type)	**Quality assessment tool**	**Meta-analysis included; method of meta-analysis**	**N relevant studies in review** (n participants)	**N tools included**
Chou[87] (2013)<br><br>Accuracy<br>Effectiveness	Adults (age ≥18y); acute care hospital, long-term and rehabilitation facilities, operative and postoperative, community (home care and wheelchair users)	PI risk assessment tools; NS	KQ1[B]: controlled or comparative randomised and nonrandomised trials, controlled or comparative observational studies<br>KQ2[B]: prospective studies of predictive validity (case-control excluded)	MEDLINE; CINAHL; Cochrane Library; grant databases; clinical trial registries	1946-2021 (MEDLINE), 1988-2012 (CINAHL), inception- 2012 (Cochrane library); English; conference abstracts excluded	Criteria consistent with AHRQ Methods Guide for Effectiveness and Comparative Effectiveness Reviews	No; presented median accuracy results	KQ1[B]: 3<br>KQ2[B]: 47	KQ1[B]: 4<br>KQ2[B]: 20
Garcia-Fernandez[88] (2014)<br><br>Accuracy	No PIs at baseline; NS	PI risk assessment tools; NS	Controlled clinical trials, prospective cohort	Cochrane Library; Center for Reviews and Dissemination University of York; LILACS; CUIDEN Plus; Spanish Medical Index	1962-2010; no restriction; peer-reviewed journal article	CASP for RCT/cohort studies	Yes; random-effects model	70 (30327)	28
Gaspar[89] (2019)<br><br>Effectiveness	Adult inpatients; hospital wards or any acute unit	PI prevention strategies; NS	Prospective or retrospective; cross-sectional, comparative, pre-test and post-test, quasi-experimental, experimental, RCT, mixed-method	MEDLINE; CINAHL; PubMed; Web of Science; EBSCO Nursing & Allied Health; Cochrane Central Register of Controlled Trials; Library, Information Science & Technology Abstracts; MedicLatina	2009-2018; English, French, Portuguese, Spanish; peer-reviewed	Evidence-Based Librarianship Critical Appraisal checklist	No	1 (1231)	2

Review author (publication year)<br><br>Review question	Eligibility criteria			Review methods				Volume of evidence	
	**Population; setting**	**Prediction tools; PI classification system**	**Study design**	**Databases searched**	**Publication restrictions** (year; language; publication type)	**Quality assessment tool**	**Meta-analysis included; method of meta-analysis**	**N relevant studies in review** (n participants)	**N tools included**
He[90] (2012)<br><br>Accuracy	NS; surgical	Braden; NS	Studies assessing predictive validity	PubMed; Web of Science	Not stated-2011; NS; NS	QUADAS	Yes; DerSimonian and Laird random-effects model, SROC analysis	3 (609)	1
Health Quality Ontario[91] (2009)<br><br>Effectiveness	Any population at risk of developing PIs; NS	PI risk assessment tools; NS	Systematic reviews, RCTs, non-randomised controlled clinical trials	MEDLINE; MEDLINE In-Process; CINAHL; EMBASE; Cochrane Library; other non-indexed citations;	1997-2008; English; NS	Criteria name not given	No	3 (528)	3
Huang[92] (2021)<br><br>Accuracy	Inpatients aged ≥18y, no PIs at admission; NS	Braden; accepted standards (NPUAP, EPUAP, AHCPR, ICD-9, Bergstrom, others)	Cross-sectional, cohort	PubMed; CINAHL; EMBASE; Web of Science; Cochrane Library; bibliographies	Inception-2020; NS; NS	QUADAS-II	Yes; bivariate model, SROC analysis	60 (49326)	1
Kottner[93] (2009)<br><br>Effectiveness (reliability)	NS; NS	Waterlow; NS	Inter- and intra-rater reliability and agreement	MEDLINE; EMBASE; CINAHL	1985-2008; English, German; original research	Own criteria	No	8	2
Lovegrove[94] (2021)<br><br>Effectiveness	Adults (age ≥18y); acute hospital care	PI risk assessment tools; NS	Primary research	MEDLINE; EMBASE; EBSCO CINAHL; EBSCO; Scopus; Web of Science	2010-2020; English; conference abstracts, posters excluded	JBI tools or analytical cross-sectional study appraisal checklist	No	5 (1910)	5
Lovegrove[95] (2018)<br><br>Effectiveness	Adults; hospital or acute care	PI risk assessment; NS	Primary research	MEDLINE; CINAHL; Scopus; Web of Science	2007-2017; English; non-research publications excluded	JBI tools	No	20	5[b]

Review author (publication year)<br><br>Review question	Eligibility criteria			Review methods				Volume of evidence	
	**Population; setting**	**Prediction tools; PI classification system**	**Study design**	**Databases searched**	**Publication restrictions** (year; language; publication type)	**Quality assessment tool**	**Meta-analysis included; method of meta-analysis**	**N relevant studies in review** (n participants)	**N tools included**
Mehicic[96] (2024)<br><br>Accuracy Effectiveness (reliability, measurement error and convergent validity)	Adults (age ≥18y); ICU<br><br>For reliability assessment: sample of nurse-raters required	Braden; NS	Primary quantitative or mixed-methods research studies	CINAHL, EMBASE, MEDLINE, Scopus and Web of Science	Database inception - 2023; English language; Peer-reviewed	COSMIN RoB checklist	No	34 (59325)	1
Moore[97] (2019)<br><br>Effectiveness	People without PIs, any age; any healthcare setting	PI risk assessment tools; validated PI staging system	RCTs or cluster-RCTs	MEDLINE; EMBASE; CINAHL; Cochrane Wounds Specialised Register; Cochrane Central Register of Controlled Trials	Start date between 1937-1974, until 2018; no restrictions; no restrictions	Cochrane RoB tool	No	2 (1487)	3
Pancorbo-Hidalgo[98] (2006)<br><br>Accuracy Effectiveness	No PIs at baseline; NS	PI risk assessment tools; NS	Controlled clinical trials, prospective cohort	MEDLINE; CINAHL; EBSCO; ScienceDirect; Current contents; DARE; Indice medico espanol; LILACS; CUIDEN; Cochrane Library; Springer; InterSciencia; ProQuest; Pascal	1966-2003; Spanish, English, French, Portuguese; no restrictions	CASP Guide for clincial trials; critical assessment guide for PI assessment and prevention for cohort studies	Yes; weighted average values using inverse of variance for weights (for accuracy measures), DerSimonian and Laird random-effects model (for OR)	33	13
Park[99] (2016a)<br><br>Accuracy	NS; NS	Modified Braden, Waterlow, Norton, Cubbin & Jackson; NPUAP, EPUAP, AHCPR, Torrence Developmental	NS	MEDLINE; EMBASE; CINAHL; Cochrane Library; KoreaMed; NDSL; KERIS	NS-2013; NS; NS	QUADAS-II	Yes; random-effects model, SROC analysis	17 (6143)	5

Review author (publication year)<br><br>Review question	Eligibility criteria			Review methods				Volume of evidence	
	**Population; setting**	**Prediction tools; PI classification system**	**Study design**	**Databases searched**	**Publication restrictions** (year; language; publication type)	**Quality assessment tool**	**Meta-analysis included; method of meta-analysis**	**N relevant studies in review** (n participants)	**N tools included**
		Classification of Pressure Sore							
Park[100] (2016b)<br><br>Accuracy	Elderly (age ≥60y); NS	Braden, Waterlow, Norton; NS	NS	MEDLINE; EMBASE; CINAHL; Cochrane database; KoreaMed	1966-2013; NS; NS	QUADAS-II	Yes; random-effects model, SROC analysis	29 (11729)	3
Park[101] (2015)<br><br>Accuracy	Adults (age ≥18y) with no PIs at baseline; hospitalised	Braden; NPUAP, AHCPR, others	Prospective	MEDLINE; EMBASE; CINAHL; KoreaMed; Cochrane Library; National Digital Science Library; Korea Education and Research Information Service	NS-2013; NS; NS	QUADAS-II	Yes; random-effects model, SROC analysis	21 (6070)	1
Pei[102] (2023)<br><br>Accuracy	Adult; hospital	ML (if ≥1 model per study, only the *'best'* was included); NS	NS	PubMed, Embase, Cochrane Library, Web of Science, CINAHL, Grey literature and "other databases"	Database inception - 2022; English and Chinese; peer-reviewed articles or full-length conference proceedings	PROBAST	Yes; random-effects model, SROC analysis	18 (408504)	18
Qu[103] (2022)<br><br>Accuracy	Adults with no PIs at baseline; hospital inpatients	ML; Munoz and Posthauer (2021) PI stage or as defined by the study authors	Diagnostic trials, crossover trials, cluster-controlled trials	MEDLINE; EMBASE; EBSCO; Web of Science	Start date between 1985-2010, until 2021; English; NS	QUADAS-II; PROBAST (but did not present PROBAST results)	Yes; fixed-effects or random-effects model dependent on heterogeneity assessment, ANOVA model for Bayesian network meta-	24 (221541)	24

Review author (publication year)<br><br>Review question	Eligibility criteria			Review methods				Volume of evidence	
	**Population; setting**	**Prediction tools; PI classification system**	**Study design**	**Databases searched**	**Publication restrictions** (year; language; publication type)	**Quality assessment tool**	**Meta-analysis included; method of meta-analysis**	**N relevant studies in review** (n participants)	**N tools included**
							analysis for diagnostic test accuracy		
Tayyib[104] (2013)<br><br>Accuracy<br>Effectiveness	Adults; ICU	NS; NPUAP/EPUAP	Quantitative	MEDLINE; PubMed; CINHAL; EBSCOHost; Cochrane Library; ProQuest; Google Scholar	2000-2012; English; journals, books, handbooks, abstracts	None	No	11 (2119)	9
Wang[105] (2022)<br><br>Accuracy	Any age; any healthcare setting	NS; NS	Primary research and sample size, except case reports or case series	PubMed; EMBASE; CINAHL; Cochrane Library	Inception-2021; English; NS	JBI tools; NOS	Yes; fixed-effects or random-effects model dependent on heterogeneity assessment	2 (992)	2
Wei[106] (2020)<br><br>Accuracy	Adults (age >18y); ICU	Braden; NS	NS	PubMed; Web of Science; Cochrane Library; SinoMed; CNKI; Wanfang	NS-2019; no restrictions; NS	QUADAS-II	Yes; DerSimonian and Laird random-effects model	11 (10044)	1
Wilchesky[107] (2015)<br><br>Accuracy	NS; long-term care	Braden; NS	NS	MEDLINE; PubMed; EMBASE; PsychINFO	1985-2013; English; journal articles (reviews and opinion papers excluded)	None	Yes; DerSimonian and Laird random-effects model	9 (40361)	1
Zhang[108] (2021)<br><br>Accuracy	Inpatient aged >18y; ICU (stay >24h)	PI risk assessment tools; standard for judging the occurrence of PI had to be described	Cohort, case-control	PubMed/MEDLINE; EMBASE; CINAHL; Web of Science; Cochrane Library; China Biomedical Literature Service System; VIP Database; CNKI	Inception-2019; no restrictions; NS	QUADAS-II	Yes; hierarchal SROC model	23 (15199)	15

Review author (publication year)<br><br>Review question	Eligibility criteria			Review methods				Volume of evidence	
	**Population; setting**	**Prediction tools; PI classification system**	**Study design**	**Databases searched**	**Publication restrictions** (year; language; publication type)	**Quality assessment tool**	**Meta-analysis included; method of meta-analysis**	**N relevant studies in review** (n participants)	**N tools included**
Zimmermann[109] (2018)<br><br>Accuracy	Adult inpatients; ICU	Any scale or index; NS	NS	MEDLINE; CINAHL; COCHRANE; El Banco de Datos de Enfermería; nursing database; LILACS	1962-2016; English, Portuguese, Spanish; NS	None	No	13	11

AHCPR – Agency for Health Care Policy and Research; CASP – Critical Appraisal Skills Programme; CNKI – China National Knowledge Infrastructure; COSMIN - COnsensus-based Standards for the selection of health Measurement INstruments; CUIDEN - Bibliographic Database Index Foundation including scientific production on Health Care in Latin American; DARE – Database of Abstracts of Reviews of Effects; DEV – model development study; EPUAP – European Pressure Ulcer Advisory Panel; FN – false negative; FP – false positive; ICU – intensive care unit; ICD-9 – International Classification of Diseases Ninth Edition; JBI – Joanna Briggs Institute; LILACS – Latin America and Caribbean Health Sciences Literature; ML – machine learning; NOS – Newcastle Ottawa Scale; NS – not stated; NPUAP – National Pressure Ulcer Advisory Panel; OR – odds ratio; PI – pressure injury; PROBAST – Prediction model Risk of Bias Assessment; QUADAS – Quality Assessment of Diagnostic Accuracy Studies; RoB – risk of bias; (S)ROC – (summary) receiver operating curve; TN - true negative; TP – true positive; ULAKBIM – Turkish Academic Network and Information Center; VAL – model validation study

[A]Patients and HCWs

[B]KQ1 – key question 1 looks at effectiveness of risk assessment tools; KQ2 – key question 2 looks at diagnostic accuracy/validity of risk assessment tools

Table C. AMSTAR-2 assessment results per review

Review author (pub. year)	ITEM 1	ITEM 2	ITEM 3	ITEM 4	ITEM 5	ITEM 6	ITEM 7	ITEM 8	ITEM 9	ITEM 10	ITEM 11	ITEM 12	ITEM 13	ITEM 14	ITEM 15	Overall confidence
**Prognostic accuracy reviews**																
Chen[85] (2023)	N	N	N	PY	Y	Y	N	PY	Y	N	N	N	N	N	Y	**Critically Low** Y=4/15 PY=2/15 N=9/15
Chen[86] (2016)	N	N	N	PY	Y	N	N	PY	PY	N	N	N	N	Y	Y	**Critically Low** Y=3/15 PY=3/15 N=9/15
Chou[87] (2013)	Y	Y	N	Y	Y	N	Y	Y	PY	Y	N	Y	Y	N	Y	**Low** Y=10/15 PY=1/15 N=4/15
Garcia-Fernandez[88] (2014)	N	N	Y	PY	N	N	N	N	N	N	Y	Y	Y	N	N	**Critically Low** Y=4/15 PY=1/15 N=10/15
He[90] (2012)	N	N	N	N	Y	N	N	N	Y	N	N	N	N	Y	Y	**Critically Low** Y=4/15 PY=0/15 N=11/15
Huang[92] (2021)	N	Y	N	PY	Y	Y	N	N	Y	N	Y	N	N	Y	Y	**Critically Low** Y=7/15 PY=1/15 N=7/15
Mehicic[96] (2024)	N	Y	N	N	Y	Y	N	N	N	N	NA	NA	N	Y	Y	**Critically Low** Y=5/13 PY=0/13 N=8/13
Pancorbo-Hidalgo[110] (2006)	N	N	Y	PY	N	Y	N	PY	N	N	N	Y	Y	N	N	**Critically Low** Y=4/15 PY=2/15 N=9/15
Park[99] (2016a)	N	N	N	PY	N	N	N	PY	N	N	N	N	N	Y	Y	**Critically Low** Y=2/15

Review author (pub. year)	ITEM 1	ITEM 2	ITEM 3	ITEM 4	ITEM 5	ITEM 6	ITEM 7	ITEM 8	ITEM 9	ITEM 10	ITEM 11	ITEM 12	ITEM 13	ITEM 14	ITEM 15	Overall confidence
																PY=2/15 N=11/15
Park[100] (2016b)	N	N	N	PY	N	Y	N	PY	N	N	N	N	N	Y	Y	**Critically Low** Y=3/15 PY=2/15 N=10/15
Park[101] (2015)	N	N	N	PY	Y	Y	N	PY	N	N	N	Y	Y	Y	Y	**Critically Low** Y=6/15 PY=2/15 N=7/15
Pei[102] (2023)	N	Y	N	Y	N	Y	N	N	Y	N	N	N	Y	N	Y	**Critically Low** Y=6/15 PY=0/15 N=9/15
Qu[103] (2022)	N	Y	N	N	Y	Y	N	N	Y	N	N	N	Y	N	Y	**Critically Low** Y=6/15 PY=0/15 N=9/15
Tayyib[104] (2013)	N	N	N	PY	N	N	N	N	N	N	NA	NA	N	N	N	**Critically Low** Y=0/13 PY=1/13 N=12/13
Wang[105] (2022)	N	N	N	PY	Y	Y	N	N	N	N	Y	N	Y	N	Y	**Critically Low** Y=5/15 PY=1/15 N=9/15
Wei[106] (2020)	N	N	N	PY	Y	Y	N	N	PY	N	N	N	Y	Y	N	**Critically Low** Y=4/15 PY=2/15 N=9/15
Wilchesky[107] (2015)	N	N	N	PY	N	N	N	N	N	N	N	N	N	Y	Y	**Critically Low** Y=2/15 PY=1/15 N=12/15
Zhang[108] (2021)	N	Y	N	PY	Y	Y	N	PY	Y	N	Y	N	Y	Y	Y	**Low** Y=8/15

Review author (pub. year)	ITEM 1	ITEM 2	ITEM 3	ITEM 4	ITEM 5	ITEM 6	ITEM 7	ITEM 8	ITEM 9	ITEM 10	ITEM 11	ITEM 12	ITEM 13	ITEM 14	ITEM 15	Overall confidence
																PY=2/15<br>N=5/15
Zimmermann [109] (2018)	N	N	N	N	Y	N	N	N	N	N	NA	NA	N	N	N	**Critically Low**<br>Y=1/13<br>PY=0/13<br>N=12/13
**Summary**	**1/19 Yes**	**6/19 Yes**	**2/19 Yes**	**2/19 Yes 13/19 PY**	**12/19 Yes**	**11/19 Yes**	**1/19 Yes**	**1/19 Yes 7/19 PY**	**6/19 Yes 3/19 PY**	**1/19 Yes**	**4/16 Yes**	**4/16 Yes**	**9/19 Yes**	**10/19 Yes**	**14/19 Yes**	
**Clinical effectiveness reviews**																
Baris[84] (2015)	N	N	N	PY	N	Y	N	N	N	N	NA	NA	N	N	N	**Critically Low**<br>Y=1/13<br>PY=1/13<br>N=11/13
Chou[87] (2013)	Y	Y	N	Y	Y	N	Y	Y	Y	Y	NA	NA	Y	N	Y	**Moderate**<br>Y=10/13<br>PY=0/13<br>N=3/13
Gaspar[89] (2019)	Y	N	N	PY	Y	N	N	Y	N	N	NA	NA	N	Y	Y	**Critically Low**<br>Y=5/13<br>PY=1/13<br>N=7/13
Health Quality Ontario[91] (2009)	N	N	N	N	N	N	N	Y	N	N	NA	NA	Y	N	Y	**Critically Low**<br>Y=3/13<br>PY=0/13<br>N=10/13
Kottner[93] (2009)	N	N	N	PY	Y	Y	N	Y	PY	N	NA	NA	Y	Y	Y	**Critically Low**<br>Y=6/13<br>PY=2/13<br>N=5/13
Lovegrove[94] (2021)	Y	PY	N	PY	Y	Y	N	Y	PY	N	NA	NA	Y	Y	Y	**Low**<br>Y=7/13<br>PY=3/13<br>N=2/13

Review author (pub. year)	ITEM 1	ITEM 2	ITEM 3	ITEM 4	ITEM 5	ITEM 6	ITEM 7	ITEM 8	ITEM 9	ITEM 10	ITEM 11	ITEM 12	ITEM 13	ITEM 14	ITEM 15	Overall confidence
Lovegrove[95] (2018)	N	Y	N	PY	Y	Y	N	Y	PY	N	NA	NA	Y	Y	Y	**Low** Y=7/13 PY=2/13 N=4/13
Mehicic[96] (2024)	Y	Y	N	N	Y	Y	N	N	PY	N	NA	NA	Y	N	Y	**Critically Low** Y=6/13 PY=1/13 N=6/13
Moore[97] (2019)	Y	Y	N	Y	Y	N	Y	Y	Y	Y	NA	NA	Y	Y	Y	**High** Y=11/13 PY=0/13 N=2/13
Pancorbo-Hidalgo[110] (2006)	N	N	Y	PY	N	Y	N	Y	PY	N	NA	NA	Y	N	N	**Critically Low** Y=4/13 PY=2/13 N=7/13
Tayyib[104] (2013)	N	N	N	PY	N	N	N	PY	N	N	NA	NA	N	N	N	**Critically Low** Y=0/13 PY=2/13 N=11/13
**Summary**	**5/11 Yes**	**4/11 Yes 1/11 PY**	**1/11 Yes**	**2/11 Yes 7/11 PY**	**7/11 Yes**	**6/11 Yes**	**2/11 Yes**	**8/11 Yes 1/11 PY**	**2/11 Yes 5/11 PY**	**2/11 Yes**	**11/11 NA**	**11/11 NA**	**8/11 Yes**	**5/11 Yes**	**8/11 Yes**	

Item 1 – Adequate research question/ inclusion criteria?; Item 2 – Protocol and justifications for deviations?; Item 3 – Reasons for study design inclusions?; Item 4 – Comprehensive search strategy?; Item 5 – Study selection in duplicate?; Item 6 – Data extraction in duplicate?; Item 7 – Excluded studies list (with justifications)?; Item 8 – Included studies description adequate?; Item 9 – Assessment of RoB/quality satisfactory?; Item 10 – Studies' sources of funding reported?; Item 11 – Appropriate statistical synthesis method?; Item 12 – Assessment of impact of RoB on synthesised results?; Item 13 – Assessment of impact of RoB on review results?; Item 14 – Discussion/investigation of heterogeneity?; Item 15 – Conflicts of interest reported?
RoB – Risk of Bias; Y – Yes; PY – Partial Yes; N – No. Further details on AMSTAR items are given in Appendix D.

Table D. Statistical synthesis results of accuracy (from all 16 reviews, that conducted meta-analysis or statistical synthesis), by prediction tool

**Review author** (publication year)	**n studies; N partic-ipants**	**Brief description of included studies**	**Brief description of included study quality**	**Summary estimates of accuracy parameters (main results from statistical syntheses)**					
				**Sensitivity (95% CI)**	**Specificity (95% CI)**	**Likelihood ratios (95% CI)**	**DOR (95% CI)**	**AUROC (95% CI)**	**Predictive values (95% CI)**
**TOOL: Braden (1987)** [111, 112]									
Huang[92] (2021)	n = 60;<br>N = 49,326	Setting: hospital (n=45; includes 22 in ICU or other acute units), LTCF (n=15)<br>Sample size: 25 to 10,098<br>Mean age: range 31.7±10.9 to 84.6±7.9<br>Design: 47 prospective, 13 retrospective<br>Braden cut-off (out of 23): range ≤10 to ≤20	QUADAS-II:<br>Patient selection: low RoB in 11 (18%) studies, unclear RoB (>50%); low concern about applicability in 44 (73%) studies, high concern for applicability in remaining 16;<br><br>Index test: low RoB in 39 (65%) studies, high RoB (approx. 33%); low concern about applicability in 51 (85%) studies;<br><br>Reference standard: low RoB in 58 (97%) studies, unclear RoB in 2 (3%); low concern about applicability in 51 (85%) studies, unclear concern in 9 (15%);<br><br>Flow and timing: low RoB in 50 (83%) studies; unclear RoB in remaining 10 (17%).	**0.78 (0.74, 0.82)**<br>N=15,241<br><br>By cut-off:<br><br>*≤15* (n=15): 0.79 (0.76, 0.82)<br><br>*16* (n=19): 0.75 (0.67, 0.82)<br><br>*17* (n=4): 0.69 (0.61, 0.76)<br><br>*18* (n=15): 0.82 (0.73, 0.89)<br><br>*≥19* (n=7): 0.78 (0.65, 0.87)	**0.72 (0.66, 0.78)**<br>N=34,085<br><br>By cut-off:<br><br>*≤15*:<br>0.66 (0.55, 0.75)<br><br>*16*:<br>0.85 (0.70, 0.93)<br><br>*17*:<br>0.86 (0.50, 0.97)<br><br>*18*:<br>0.70 (0.62, 0.77)<br><br>*≥19*:<br>0.54 (0.44, 0.63)	**PLR 2.80 (2.30, 3.50)**<br><br>**NLR 0.30 (0.26, 0.35)**	**9.00 (7.00, 13.00)**<br><br>By cut-off:<br><br>*≤15*:<br>7.00 (4.00, 12.00)<br><br>*16*:<br>17.00 (8.00, 36.00)<br><br>*17*:<br>14.00 (2.00, 103.00)<br><br>*18*:<br>11.00 (6.00, 20.00)<br><br>*≥19*:<br>4.00 (2.00, 7.00)	**0.82 (0.79, 0.85)**<br><br>By cut-off:<br><br>*≤15*:<br>0.80 (0.76, 0.83)<br><br>*16*:<br>0.84 (0.80, 0.87)<br><br>*17*:<br>0.73 (0.69, 0.77)<br><br>*18*:<br>0.83 (0.79, 0.86)<br><br>*≥19*:<br>0.67 (0.63, 0.71)	
Zhang[108] (2021)	n = 18;<br>N = 11,167	Overview of studies on Braden not reported.<br><br>Braden cut-off points used range from 10.5 to 20.	Overview of studies on Braden not reported.<br><br>QUADAS-II:<br>Overall judgement was "not so satisfactory".	**0.78 (0.68, 0.85)**	**0.61 (0.40, 0.79)**	**PLR 2.00 (1.24, 3.24)**<br><br>**NLR 0.36 (0.25, 0.52)**	**5.52 (2.61, 11.67)**	**0.78**	

Review author (publication year)	n studies; N partic-ipants	Brief description of included studies	Brief description of included study quality	Summary estimates of accuracy parameters (main results from statistical syntheses)					
				**Sensitivity (95% CI)**	**Specificity (95% CI)**	**Likelihood ratios (95% CI)**	**DOR (95% CI)**	**AUROC (95% CI)**	**Predictive values (95% CI)**
Wei[106] (2020)	n = 11; N = 10,044	Prospective (7/11); retrospective (4/11). ICU settings. Mean age from 49.2±17.3 to 62.5±16.3. Classification systems: PPPU (n=1), NPUAP(n=5), EPUAP (n=2), ICD-9 (n=1), NS (n=2). Braden scale cut-off: 11 (n=2), 12 (n=1), 13 (n=3), 14 (n=2), 16 (n=2), 18 (n=1)	QUADAS-II: Limited detail. No study was considered low risk and low concern for applicability in all domains.<br><br>All 11 studies met 80% "low risk" or "yes" in 13 questions.	**0.89 (0.87, 0.91)**<br><br>n=1123 I^2 = 94.9%, p<0.001<br><br>Q*=0.720 (0.66, 0.78) (SE 0.03)	**0.28 (0.27, 0.29)**<br><br>n=8921 I^2 = 99.2%, p<0.001		**6.29 (4.09, 9.68)**	**0.78 (0.72, 0.85)**	
Chen[86] (2016)	n = 8; N = 41,489	Settings: nursing homes, LTCF, tertiary care hospitals, veterans' medical centres, and skilled nursing facilities. NPUAP used as reference standard, Stage I-IV (prevalence 6.4% to 30.1%). Braden scale cut-off ranged from 17 to 20.	QUADAS (original): All studies scored 'No' for two items (reporting of uninterpretable/intermediate results and explanation of any withdrawals). Five studies judged 'Yes' on remaining 9 QUADAS items; 3 studies judged 'Unclear' for reporting of selection criteria.	**0.80 (0.79, 0.81)**<br><br>I^2 = 97.4%; Q* 0.709 (0.63, 0.79) (SE 0.04)	**0.42 (0. 42, 0.43)**<br><br>I^2 = 98.7%		**5.66 (3.77, 8.48)**<br><br>I^2 = 96.4%; τ^2 = 0.4173	**0.769 (0.675, 0.862)**<br><br>(SE 0.048)	
Park[100] (2016b)	n = 25; N = 10,547	Overview of studies on Braden not reported. Cut-offs selected "by following the one which the study researcher(s) indicated to be the most effective". Braden cut-off: 13 (n=2); 16 (n=8); 17 (n=2); 18 (n=9); 19 (n=3); 20 (n=1)	Overview of studies on Braden not reported.	**0.72 (0.69, 0.74)**<br><br>I^2 = 79.9% (χ^2=119.57, p<0.001)<br><br>Q* = 0.72 (SE = 0.02)	**0.63 (0.62, 0.64)**<br><br>I^2 = 96.4% (χ^2=673.34, p<0.001)	**PLR 2.31 (1.98, 2.69)**<br><br>**NLR 0.43 (0.36, 0.51)**	**6.50 (4.64, 9.11)**	**0.79** (SE = 0.02)	
Park[101] (2015)	n = 21; N = 6,070	Settings: ICU, hospital, nursing units, trauma centre Average age: >50% of studies in 50s or 60s Reference standard: NPUAP	QUADAS-II: "None of the studies were evaluated to have a high risk of bias in each area. ... 1) they only included prospective studies, 2)	**0.72 (0.68, 0.75)**<br><br>I^2 = 64.0% (χ^2= 55.48, p<0.001)	**0.81 (0.80, 0.82)**<br><br>I^2 = 96.2% (χ^2= 25.38, p<0.001)	**PLR 3.43 (2.66, 4.44)**<br><br>**NLR 0.38 (0.30, 0.48)**	**10.30 (6.65, 15.96)**	**0.84** (SE 0.02)	

Review author (publication year)	n studies; N partic-ipants	Brief description of included studies	Brief description of included study quality	Summary estimates of accuracy parameters (main results from statistical syntheses)					
				Sensitivity (95% CI)	Specificity (95% CI)	Likelihood ratios (95% CI)	DOR (95% CI)	AUROC (95% CI)	Predictive values (95% CI)
		(n=10), AHCPR (n=2), TDCPS (n=1), tools developed by individual researchers (n=6); NS (n=2).<br>Evaluation conducted at time of or within 24-72hr of hospitalization. Cut-off points ranged from <13 to <20.	test diagnosis measure and reference standards test were non-invasive and evaluated by regular observation by nurses, which means that almost none of the patients were excluded." "All selected studies were confirmed to be of high quality and meet all areas of the quality evaluation"						
Wilchesky [107] (2015)	n = 11;<br>N = 40,361	Assessed predictive validity (n=4; 1,145 patients); mean PI prevalence 20.71%; Braden cut-off 18<br><br>Assessed concurrent/ screening validity (n=7; 39,216 patients); mean PI prevalence 15.85%; Braden cut-off 20 in all except one study using 18	Not done	**0.86 (0.85, 0.87)**<br><br>Overall: mean 0.84 (SD = 0.083, range 0.70–0.95)<br><br>Predictive: mean 0.74<br>Concurrent: mean 0.89	**0.39 (0.38, 0.39)**<br><br>Overall: mean 0.51 (SD = 0.168, range 0.34–0.74)<br><br>Predictive: mean 0.72<br>Concurrent: mean 0.39		RR reported.<br>**RR = 4.33 (3.28, 5.72)**<br>χ^2=107.51, p<0.001<br><br>Predictive: RR = 4.53 (3.42, 6.00)<br>χ^2=111.65, p<0.001<br>Concurrent: RR = 4.22 (3.02, 5.89)<br>χ^2 = 71.2, p<0.001		Overall:<br>**mean PPV 0.28** (SD=0.151, range 0.09–0.54);<br>**mean NPV 0.93** (SD=0.053, range 0.82–0.98)<br><br>Predictive validity: mean PPV 0.42, NPV 0.91<br><br>Concurrent validity: mean PPV 0.21, NPV 0.94

Review author (publication year)	n studies; N partic-ipants	Brief description of included studies	Brief description of included study quality	Summary estimates of accuracy parameters (main results from statistical syntheses)					
				Sensitivity (95% CI)	Specificity (95% CI)	Likelihood ratios (95% CI)	DOR (95% CI)	AUROC (95% CI)	Predictive values (95% CI)
Garcia-Fernandez [88] (2014)	n = 33; N = 8,615	Not reported	Not reported.<br>Included studies passed CASP quality assessment.<br><br>(13/86 studies excluded across whole review)				RR reported.<br>**RR = 4.26 (3.27, 5.55)**<br><br>I^2 = 68%;<br>τ^2 = 0.37		
Chou[87] (2013)	n = 32 (33 publica-tions);<br>N = 11,596	Overview of studies on Braden not reported.<br><br>Braden cut-offs ranged between ≤10 and ≤20. The majority of studies used cut-offs of ≤15 (n=12), ≤16/<17 (n=11) or ≤18 (n=16).	Overview of studies on Braden not reported.<br><br>Criteria based on QUADAS-II: Studies evaluating Braden: Rated good quality (n=13), fair quality (n=18) and poor quality (n=2).	Median[A] by cut-off:<br><br>*≤10* (n=1): 0.91<br><br>*≤15* (n=12): 0.33 (range 0.09 - 0.82)<br><br>*≤16* (n=8): 0.77 (range 0.35 - 1.0)<br><br>*≤18* (n=16): 0.74 (range 0.33 - 1.0)<br><br>*≤20* (n=1): 0.97	Median[A] by cut-off:<br><br>*≤10* (n=1): 0.96<br><br>*≤15* (n=12): 0.91 (range 0.67 - 0.95)<br><br>*≤16* (n=8): 0.64 (range 0.14 - 1.0)<br><br>*≤18* (n=16): 0.68 (range 0.34 - 0.86)<br><br>*≤20* (n=1): 0.05	PLR ranged from 22.75 (cut-off ≤10) and 1.00 (cut-off <17).<br><br>NLR ranged from 0.09 (cut-off ≤10) and 1.00 (cut-off <17).		Median in n=7 studies that reported AUROC.<br><br>**0.77 (range 0.55 - 0.88)**	
He[90] (2012)	n = 3;<br>N = 609	Study countries: South Korea, Germany and USA; Study design: two prospective, one NS; all surgical ICU populations; mean age ranges from 58.1-62.0 years; Assessment time: two pre-operative, one NS;	QUADAS (original):<br>Two studies at high risk of bias due to the spectrum of patients not being representative of those who will receive the test in practice. One study at unclear risk of bias. It was unclear whether the whole sample or a random sample	**0.42 (0.38, 0.47)**	**0.84 (0.83, 0.85)**		**4.40 (2.98, 6.50)**	**0.6921** (SE 0.0346)	

Review author (publication year)	n studies; N partic-ipants	Brief description of included studies	Brief description of included study quality	Summary estimates of accuracy parameters (main results from statistical syntheses)					
				**Sensitivity (95% CI)**	**Specificity (95% CI)**	**Likelihood ratios (95% CI)**	**DOR (95% CI)**	**AUROC (95% CI)**	**Predictive values (95% CI)**
		prevalence: 18.3%, 49.0%, 4.7%; Cut off points: one study used 14, two studies used various ranging from 9-20, and 5-23.	received a diagnosis with the reference standard and withdrawals from the study were unclear.						
Pancorbo-Hildago[110] (2006)	n = 20; N = 6,443 (included in aggregated analysis)<br><br>n = 16; N = 5,847 (included in MA)	Hospital medical, cardiovascular, orthopaedic and surgical units, hospital acute care, hospital extended care, home care, hospice, long-term care facilities, veterans administration medical centres, skilled nursing facilities, hospital internal medicine, hospital cardiac surgery; classification system not reported but minimum PI stage I or II; Braden cut-offs ≤14, 16, 17, 18, 19, 20; follow-up period ranges from 5 days to 3 months/whole stay in unit/discharge/ death	Studies excluded if considered not to be 'valid'. This was determined by assessment of methodological quality; CASP guide for RCTs and a clinical assessment guide developed for the clinical practice guide for PI assessment and prevention for prospective cohort studies.	**0.57**	**0.68**		**OR = 4.08 (2.56, 6.48)**		**PPV 0.23**<br>**NPV 0.91**
**TOOL: Modified Braden scales: Braden – modified by Song & Choi[113] (1991)**									
Park[99] (2016a)	n = 4; N = 688	Prospective (4/4), recruiting patients with no PI at baseline (hospital ward (n=2) or ICU (n=3); mean age in the 50s (n=2), 60s (n=2). Classification used: AHCPR (n=3), Bergstrom (n=1). Braden scale cut-off used: <21 (n=1), <23 (n=1), <24 (n=2)	QUADAS-II:<br>"None had 'high risk'"	**0.97 (0.92, 0.99)**<br><br>n=125<br>Q* 0.90 (0.03)	**0.70 (0.66, 0.73)**<br><br>n=563<br>Q* 0.90 (0.03)	**PLR 3.47 (1.33, 9.06)**<br><br>**NLR 0.08 (0.04, 0.19)**	**56.56 (21.88, 146.21)**	**0.95** (SE 0.02)	

Review author (publication year)	n studies; N partic-ipants	Brief description of included studies	Brief description of included study quality	Summary estimates of accuracy parameters (main results from statistical syntheses)					
				**Sensitivity (95% CI)**	**Specificity (95% CI)**	**Likelihood ratios (95% CI)**	**DOR (95% CI)**	**AUROC (95% CI)**	**Predictive values (95% CI)**
Garcia-Fernandez [88] (2014)	n = 3; N = 476	Not reported	Not reported. Included studies passed CASP quality assessment.				RR reported. **RR = 26.06 (9.01, 75.39)** I^2 = 0%; τ^2 = 0		
**TOOL: Braden – modified by Pang & Wong[114] (1998)**									
Park[99] (2016a)	n = 2; N = 626	Prospective (2/2), recruiting patients with no PI at baseline (OS ward (n=1) or NS (n=1); mean age 79.4 and 54.1. Classification used: NPUAP (n=2) Braden scale cut-off used: <19 (n=1), <14 (n=1)	QUADAS-II: "None had 'high risk'"	**0.89 (0.71, 0.98)** n=27 Q* not calculated	**0.71 (0.67, 0.75)** n=599 Q* not calculated	**PLR 2.87 (1.88, 4.38)** **NLR 0.17 (0.06, 0.49)**	**16.06 (4.75, 54.35)**	Not calculated	
**TOOL: Braden – modified by Kwong[115] (2005)**									
Garcia-Fernandez [88] (2014)	n = 2; N = 626	Not reported	Not reported. Included studies passed CASP quality assessment.				RR reported. **RR = 13.68 (4.19, 44.64)** I^2 = 0%; τ^2 = 0		
**TOOL: Cubbin & Jackson[116] (1991)**									
Chen[85] (2023)	n = 9; N = 7,684	Study designs included prospective studies (n=2), a prospective and cross-sectional study (n=1), retrospective studies (n=3), an observational study (n=1), a predictive correlational study (n=1) and a longitudinal study (n=1). Studies were conducted in	Authors state that *"none of [the 9 included studies] has a high risk of bias in any field"*, despite indicating some as 'high risk' in the table of QUADAS-2 results. QUADAS-2: Patient selection: low RoB in 5 (56%) studies, unclear RoB in 1 (11%) study, high RoB in 3 (33%),	**0.81 (0.51, 0.95)** n=1,558	**0.76 (0.58, 0.88)** n=6,126	**PLR 3.34 (2.14, 5.21)** **NLR 0.25 (0.09, 0.68)**	**13.24 (5.41, 32.40)**	**0.84 (0.81, 0.87)**	

Review author (publication year)	n studies; N partic-ipants	Brief description of included studies	Brief description of included study quality	Summary estimates of accuracy parameters (main results from statistical syntheses)					
				**Sensitivity (95% CI)**	**Specificity (95% CI)**	**Likelihood ratios (95% CI)**	**DOR (95% CI)**	**AUROC (95% CI)**	**Predictive values (95% CI)**
		South Korea (n=3), the US (n=2), Turkey (n=2), China (n=1) and Portugal (n=1). Studies targeted research participants >18 years old (n=6), >16 years old (n=1), ≥21 years old (n=1) or did not set an age limit (n=1). All studies restricted to patients without PIs on ICU admission.	low applicability concerns in all studies;<br>Index test: low RoB in 4 (44%) studies, unclear RoB in 3 (33%) studies, high RoB in 2 (22%) studies, low applicability concerns in 7 (78%) studies, high applicability concerns in 2 (22%) studies;<br>Reference standard: low RoB in 1 (11%) study, unclear RoB in 6 (67%) studies), high RoB in 2 (22%) studies, low applicability concerns in 4 (44%) studies, unclear applicability concerns in 2 (22%) studies, high applicability concerns in 3 (33%) studies;<br>Flow and timing: low RoB in 7 (78%) studies, unclear RoB in 2 (22%) studies						
Zhang[108] (2021)	n = 6;<br>N = 800	Overview of studies on C&J not reported.<br><br>All 6 prospective studies.	Overview of studies on C&J not reported.	**0.84 (0.59, 0.95)**	**0.84 (0.66, 0.93)**	**PLR 5.12 (2.70, 9.70)**<br><br>**NLR 0.19 (0.08, 0.49)**	**26.45 (13.51, 51.78)**	**0.9**	
Park[99] (2016a)	n = 4;<br>N = 662	Prospective (4/4); ICU patients for all studies (1 in surgical ICU), with no PI at baseline (n=3); mean age in the 50s (n=2), 60s (n=2). Classification used: AHCPR (n=2), NPUAP (n=1), Lowthian (n=1).	QUADAS-II:<br>"None had 'high risk'"	**0.67 (0.60, 0.74)**<br><br>n=194<br>Q* = 0.75 (0.06)	**0.75 (0.71, 0.79)**<br><br>n=468. Q* 0.75 (0.06)	**PLR 2.80 (1.66, 4.72)**<br><br>**NLR 0.34 (0.15, 0.76)**	**9.46 (2.41, 37.22)**	**0.82**<br>(SE 0.06)	

Review author (publication year)	n studies; N partic-ipants	Brief description of included studies	Brief description of included study quality	Summary estimates of accuracy parameters (main results from statistical syntheses)					
				Sensitivity (95% CI)	Specificity (95% CI)	Likelihood ratios (95% CI)	DOR (95% CI)	AUROC (95% CI)	Predictive values (95% CI)
		C&J scale cut-off used: <24 (n=2), <26 (n=1), <28 (n=1)							
Garcia-Fernandez [88] (2014)	n = 3; N = 370	Not reported	Overview of studies on C&J not reported.				RR reported. **RR = 8.63 (3.02, 24.66)**<br><br>I^2 = 65%; τ^2 = 0.55		
**TOOL: Cubbin & Jackson – Revised: "Jackson & Cubbin"[117] (1999)**									
Garcia-Fernandez [88] (2014)	n = 2; N = 259	Not reported	Overview of studies on J&C not reported.				RR reported. **RR = 3.16 (1.49, 6.71)**<br><br>I^2 = 0%; τ^2 = 0		
**TOOL: EMINA[118] (2001)**									
Garcia-Fernandez [88] (2014)	n = 2; N = 861	Not reported	Overview of studies on EMINA not reported.				RR reported. **RR=6.17 (3.46, 11.01)**<br><br>I^2 = 0%; τ^2 = 0		
**TOOL: EVARUCI[119] (2001)**									
Zhang[108] (2021)	n = 3; N = 3,063	Overview of studies on EVARUCI not reported.<br><br>All 3 prospective studies.	Overview of studies on EVARUCI not reported.	**0.84 (0.79, 0.89)**	**0.68 (0.66, 0.70)**	**PLR 2.32 (2.14, 2.51)**<br><br>**NLR 0.25 (0.19, 0.35)**	**9.79 (6.81, 14.07)**	**0.82**	
**TOOL: Norton[120] (1962)**									
Park[99] (2016a)	n = 7; N = 2,899	Prospective (6/7); inpatients with no PI at baseline (1 LTC, 2 'hospital', 1 ICU, 1 ICU & wards); mean age in the 50s (n=1), 60s (n=3), or 80s (n=1),	QUADAS-II: "None had 'high risk'"	**0.75 (0.70, 0.79)**<br><br>n=383	**0.57 (0.55, 0.59)**<br><br>n=2516	**PLR 1.77 (1.26, 2.50)**<br><br>**NLR 0.49 (0.32-0.76)**	**7.57 (2.53, 22.64)**	**0.82** (SE 0.05)	

Review author (publication year)	n studies; N partic-ipants	Brief description of included studies	Brief description of included study quality	Summary estimates of accuracy parameters (main results from statistical syntheses)					
				**Sensitivity (95% CI)**	**Specificity (95% CI)**	**Likelihood ratios (95% CI)**	**DOR (95% CI)**	**AUROC (95% CI)**	**Predictive values (95% CI)**
		or NS (n=2).<br>Classification used: AHCPR (n=3), NPUAP (n=2), EPUAP (n=1), TDCPS (n=1).<br>Norton scale cut-off used: <14 (n=2, but reported as 3 in paper), <15 (n=2), <16 (n=3)		Q* 0.75 (SE=0.04)	Q* 0.75 (SE=0.04)				
Park[100] (2016b)	n = 5; N = 2,408	Only reported overall, not by scale.<br>Norton cut-offs: 14 (n=2); 16 (n=3)	Only reported overall, not by scale.	**0.76 (0.71, 0.80)**<br><br>I^2 = 90.5% (χ^2 = 41.97, p<0.001)	**0.55 (0.53, 0.57)**<br><br>I^2 98.7% (χ^2 =308.41, p<0.001)	**PLR 1.58 (1.07, 2.34)**<br><br>**NLR 0.47 (0.29, 0.76)**	**6.41 (1.72, 23.88)**	**0.84**<br>(SE 0.07)	
Garcia-Fernandez [88] (2014)	n = 16; N = 5,032	Not reported	Not reported.<br>Included studies passed CASP quality assessment.				RR reported.<br>**RR = 3.69 (2.64, 5.16)**<br><br>I^2 = 66%; τ^2 = 0.23		
Chou[87] (2013)	n = 9; N = 5,444	Overview of studies on Norton not reported.<br><br>Norton cut-offs ranged between <12 and ≤16. The majority of studies used cut-offs of ≤14 (n=5), ≤16 (n=3).	Overview of studies on Norton not reported.<br><br>Criteria based on QUADAS-II: Rated as fair quality (n=6) and good quality (n=3).	Median[A] by cut-off:<br><br>*≤12* (n=1): 0.62<br><br>*≤14* (n=5): 0.75 (range 0.0 - 0.89)<br><br>*≤16* (n=3): 0, 0.75, 0.89	Median[A] by cut-off:<br><br>*≤12* (n=1): 0.72<br><br>*≤14* (n=5): 0.68 (range 0.59 - 0.95)<br><br>*≤16* (n=3): 0.55, 0.59, 0.6.	PLR ranged from 1.83 (cut-off ≤16) to 2.34 (cut-off ≤14).<br><br>NLR ranged from 0.37 (cut-off ≤14) to 0.53 (cut-off <12).		Median in n=3 studies that reported AUROC.<br><br>**0.74 (range 0.56 - 0.75)**	

Review author (publication year)	n studies; N participants	Brief description of included studies	Brief description of included study quality	Summary estimates of accuracy parameters (main results from statistical syntheses)					
				Sensitivity (95% CI)	Specificity (95% CI)	Likelihood ratios (95% CI)	DOR (95% CI)	AUROC (95% CI)	Predictive values (95% CI)
Pancorbo-Hidalgo[110] (2006)	n = 5; N = 2,008 (included in aggregated analysis & MA)	Hospital cardiovascular surgery and neurosurgery, hospital orthopaedic surgery, geriatric centre, rehabilitation hospital medical and orthopaedic units, hospital medical units, surgical units and ICU; classification system not reported but minimum PI stage I (n=5) or II (n=1); Norton cut-offs ≤14 (n=2), ≤16 (n=3); follow-up period from 2 weeks-12 weeks/to discharge/death; average age, years = 53.1, 60.1, 80.4, NS (n=2).	All studies considered to be 'valid' through use of CASP.	**0.47**	**0.62**		**OR = 2.16 (1.03, 4.54)**		**PPV 0.18** **NPV 0.87**
**TOOL: Norton – modified by Ek[121] (1987)**									
Garcia-Fernandez [88] (2014)	n = 3; N = 502	Not reported	Not reported. Included studies passed CASP quality assessment.				RR reported. **RR = 2.38 (0.92, 6.12)** I^2 = 81%; τ^2 = 0.56		
**TOOL: Norton – modified by Bienstein[122] (1991)**									
Garcia-Fernandez [88] (2014)	n = 2; N = 164	Not reported	Not reported. Included studies passed CASP quality assessment.				RR reported. **RR = 1.53 (1.11, 2.12)** I^2 =0%; τ^2 =0		

Review author (publication year)	n studies; N partic-ipants	Brief description of included studies	Brief description of included study quality	Summary estimates of accuracy parameters (main results from statistical syntheses)					
				Sensitivity (95% CI)	Specificity (95% CI)	Likelihood ratios (95% CI)	DOR (95% CI)	AUROC (95% CI)	Predictive values (95% CI)
**TOOL: PSPS[123] (1987)**									
Garcia-Fernandez [88] (2014)	n = 2; N = 1,956	Not reported	Not reported.<br>Included studies passed CASP quality assessment.				RR reported.<br>**RR = 21.40 (10.74, 42.63)**<br><br>I^2 =0%;<br>τ^2 = 0		
**TOOL: Waterlow[124] (1985)**									
Zhang[108] (2021)	n = 4; N = 1,000	Reported overall, not by scale.<br>All 4 prospective studies.	Reported overall, not by scale.	**0.63 (0.48, 0.76)**	**0.46 (0.22, 0.71)**	**PLR 1.16 (0.66, 2.01)**<br><br>**NLR 0.82 (0.40, 1.67)**	**1.42 (0.40, 5.07)**	**0.56**	
Park[99] (2016a)	n = 6; N = 1,268	Prospective (6/6); all male* inpatients aged over 60 on average with no PI at baseline (3 included ICU patients).<br>Classification used: AHCPR (n=2), NPUAP (n=2), EPUAP (n=1), TDCPS (n=1).<br>Waterlow scale cut-off used: <9 (n=1), <15 (n=1), <16 (n=2), <17 (n=1), NS (n=1)<br>* as reported in review's text. However, the table reports a mixture of female and male participants for all studies, with a mean female proportion of 50.73%.	QUADAS-II:<br>"None had 'high risk'"	**0.55 (0.49, 0.62)**<br><br>n=246<br>Q* 0.75 (SE=0.03)	**0.82 (0.80, 0.85)**<br><br>n=1222<br>Q* 0.75 (SE=0.03)	**PLR 2.89 (1.74, 4.79)**<br><br>**NLR 0.46 (0.31, 0.70)**	**9.22 (6.43, 13.23)**	**0.82** (SE 0.03)	
Park[100] (2016b)	n = 5; N = 1,406	Only reported overall, not by scale.	Only reported overall, not by scale.	**0.53 (0.47, 0.60)**	**0.84 (0.81, 0.86)**	**PLR 3.09 (1.63, 5.83)**	**9.06 (6.30, 13.04)**	**0.81** (SE 0.03)	

Review author (publication year)	n studies; N partic-ipants	Brief description of included studies	Brief description of included study quality	Summary estimates of accuracy parameters (main results from statistical syntheses)					
				Sensitivity (95% CI)	Specificity (95% CI)	Likelihood ratios (95% CI)	DOR (95% CI)	AUROC (95% CI)	Predictive values (95% CI)
		Waterlow cut-offs: 15 (n=1); 16 (n=2); 17 (n=1); NS (n=1)		I^2 = 89.0% (χ^2 = 36.31, p<0.001)	I^2 = 98.7% (χ^2 =155.55, p<0.001)	**NLR 0.49 (0.34, 0.72)**			
Garcia-Fernandez [88] (2014)	n = 14; N = 3,969	Not reported	Not reported. Included studies passed CASP quality assessment.				RR reported. **RR = 2.66 (1.76, 4.01)** <br>I^2 = 47%; τ^2 = 0.19		
Chou[87] (2013)	n = 10; N = 3,905	Overview of studies on Waterlow not reported. <br>Waterlow cut-offs ranged from >9 to ≥20.	Overview of studies on Waterlow not reported. <br>Criteria based on QUADAS-II: Studies evaluating Waterlow: Rated as good quality (n=2) and fair quality (n=8).					Median in n=4 studies that reported AUROC. <br>**0.61 (range 0.54 - 0.66)**	
Pancorbo-Hidalgo[110] (2006)	n = 6; N = 2,246 (included in aggregated weighted mean analysis) <br>n = 5; N = 2,215 (included in MA)	Hospital orthopaedic surgery, community, geriatric centre, rehabilitation hospital medical and orthopaedic units, hospital ICU, hospital medical, surgical and geriatric units; classification system not reported but minimum PI stage I (n=6) or II (n=1); Waterlow cut-offs ≥10 (n=4), NS (n=1), ≥16 (n=1), ≥15 (n=1); follow-up period from 2 weeks - 12 weeks / to discharge/death; average age, years in 50s (n=2), 60s (n=1), 80s (n=2), NS (n=2)	All studies considered to be 'valid' through use of CASP.	**0.82**	**0.27**		**OR = 2.05 (1.11, 3.76)**		**PPV 0.16** **NPV 0.89**

Review author (publication year)	n studies; N partic-ipants	Brief description of included studies	Brief description of included study quality	Summary estimates of accuracy parameters (main results from statistical syntheses)					
				**Sensitivity (95% CI)**	**Specificity (95% CI)**	**Likelihood ratios (95% CI)**	**DOR (95% CI)**	**AUROC (95% CI)**	**Predictive values (95% CI)**
**ML models:**									
Pei[102] (2023)	n = 14; N = 328,789 (included in MA)	Studies published from 2012-2022; studies conducted in China (n=4), Taiwan (n=3), USA (n=6), Germany (n=1), Japan (n=1), Australia (n=1), Spain (n=1) and Czech Republic (n=1) ;15 studies utilised retrospective data, while 3 used prospective data; studies focused on adult ICU inpatients (n=5), hospitalised patients (n=8), adult hospitalised patients awaiting surgery (n=3), cancer patients (n=1) and end-of-life adult inpatients (n=1); sample size range 168-149,006; number of patients with PI range 8-4663<br><br>Validation methods: both sample splitting and k-fold cross-validation (n= 10), sample splitting only (n=4), k-fold cross-validation only (n=1). External verification conducted (n=1). Validation methods not reported (n=2). Handling of missing data not disclosed (n=7).	PROBAST results:<br><br>Overall, 16/18 (88.9%) papers were at high ROB, 1 (5.6%) was at unclear ROB and only 1 (5.6%) was at low ROB.<br><br>14 (77.8%) studies were at high ROB in the analysis domain. The most common factors contributing to the high risk of bias in the analysis domain included an inadequate number of events per candidate predictor, poor handling of missing data and failure to deal with overfitting.	**0.79 (0.78, 0.80)**<br><br>n=18,807	**0.87 (0.88, 0.87)**<br><br>n=309,982	**PLR 10.71 (5.98, 19.19)**<br><br>**NLR 0.21 (0.08, 0.50)**	**52.39 (24.83, 110.55)**	**0.94**	
Qu[103] (2022)	n = 14; N = 118,292	Only reported overall, not by algorithm type.<br>Conducted in: hospital	Only reported overall, not by algorithm type.	**0.66 (0.42, 0.84)**	**0.90 (0.78, 0.96)**	**PLR 6.9 (3.2, 14.7)**	**18 (7, 49)**	**0.88 (0.85, 0.91)**	

Review author (publication year)	n studies; N participants	Brief description of included studies	Brief description of included study quality	Summary estimates of accuracy parameters (main results from statistical syntheses)					
				Sensitivity (95% CI)	Specificity (95% CI)	Likelihood ratios (95% CI)	DOR (95% CI)	AUROC (95% CI)	Predictive values (95% CI)
		patients (n= 13); surgical patients (n=3), ICU (n=5), CVD patients (n=2), cancer patients (n=1), LTC (n=1)<br><br>**Decision Tree** models	QUADAS-II:<br>Five studies were judged high risk of bias for reference standard, 4 of which also had high concern for applicability in the same domain Five additional studies were judged unclear risk of bias in at least one domain.<br><br>Of those studies which included the DT algorithm, two studies had an unclear risk of bias and two studies were at high risk of bias. The rest were rated as low risk of bias.	n=7557	n=110,735	**NLR 0.37 (0.20, 0.69)**			
Qu[103] (2022)	n = 14;<br>N = 195,927	**Logistic Regression** models	QUADAS-II:<br>Of those studies which included logistic regression, one study had an unclear risk of bias and four studies were at high risk of bias. The rest were rated as low risk of bias.	**0.71 (0.60, 0.80)**<br><br>n=9046	**0.83 (0.75, 0.89)**<br><br>n=186,881	**PLR 4.3 (3.1, 5.9)**<br><br>**NLR 0.35 (0.26, 0.46)**	**12 (9, 17)**	**0.84 (0.81, 0.87)**	
Qu[103] (2022)	n = 9;<br>N = 97,815	**Neural Network** models	QUADAS-II:<br>Of those studies which included neural networks, one study had an unclear risk of bias and one study was at high risk of bias. The rest were rated as low risk of bias.	**0.73 (0.55, 0.86)**<br><br>n=9488	**0.78 (0.65, 0.87)**<br><br>n=88,327	**PLR 3.3 (2.1, 5.0)**<br><br>**NLR 0.35 (0.21, 0.59)**	**9 (5, 19)**	**0.82 (0.79, 0.85)**	
Qu[103] (2022)	n = 7;<br>N = 161,334	**Random Forest** models	QUADAS-II:<br>Of those studies which included random forests, one study was at high risk of bias. The rest were rated as low risk of bias.	**0.72 (0.26, 0.95)**<br><br>n=5486	**0.96 (0.80, 0.99)**<br><br>n=155,848	**PLR 16.3 (2.4, 108.9)**<br><br>**NLR 0.29 (0.07, 1.29)**	**56 (3, 1258)**	**0.95 (0.93, 0.97)**	

Review author (publication year)	n studies; N partic-ipants	Brief description of included studies	Brief description of included study quality	Summary estimates of accuracy parameters (main results from statistical syntheses)					
				**Sensitivity (95% CI)**	**Specificity (95% CI)**	**Likelihood ratios (95% CI)**	**DOR (95% CI)**	**AUROC (95% CI)**	**Predictive values (95% CI)**
Qu[103] (2022)	n = 9; N = 152,068	**Support Vector Machine** models	QUADAS-II: Of those studies which included support vector machines, one study was at high risk of bias. The rest were rated as low risk of bias.	**0.81 (0.69, 0.90)** n=6562	**0.81 (0.59, 0.93)** n=145,506	**PLR 4.3 (1.8, 9.9)** **NLR 0.23 (0.13, 0.39)**	**19 (6, 54)**	**0.88 (0.85, 0.90)**	

AHCPR – Agency for Health Care Policy and Research; AUC/AUROC – area under the receiver operating characteristic curve; CI – confidence interval; CVD – cardiovascular disease; DOR – diagnostic odds ratio; efficacy – percentage of correctly classified patients; EPUAP – European Pressure Ulcer Advisory Panel; HSROC – hierarchical summary receiver operating characteristic curve; ICU – intensive care unit; LTC(F) – long-term care (facility); N – number of participants; n – number of studies; NLR/PLR – negative/positive likelihood ratio; NPUAP – National Pressure Ulcer Advisory Panel; NS – not stated; OR – odds ratio; PI – pressure injury; PPPU – Panel for the Prediction and Prevention of Pressure Ulcers; PPV/NPV – positive/negative predictive value; QUADAS – Quality Assessment of Diagnostic Accuracy Studies; RCT – randomised controlled trial; RoB – Risk of bias; RR – risk ratio; SE – standard error; SENS – sensitivity; SPEC – specificity; TDCPS – Torrance Developmental Classification of Pressure Sore.

[A] or individual study results, where no median was calculated.

Table E. Individual accuracy results of PI risk prediction tools (for which no meta-analysis was conducted*)

Tool (develop-ment year)	Reviews' authors (publication year)	n = no. of studies; N = no. of part-icipants	Brief description of included studies	Brief description of included study quality	Sensitivity (95% CI)	Specificity (95% CI)	Likelihood ratios (95% CI)	AUROC (95% CI)	Predictive values (95% CI)	Other outcomes
**Andersen**[125] (1982)	Garcia-Fernandez [88] (2014); Pancorbo-Hidalgo[110] (2006)	n = 1; N = 3,398	Hospital acute ward; classification system not reported but minimum PI stage I; cut-off ≥2; follow-up period 10 days; mean age NS. [110]	Passed CASP quality assessment in both reviews. No further details.	**0.88** [110]	**0.87** [110]			**PPV 0.07** **NPV 1.00** [110]	RR = 42.35 (95% CI: 16.67, 107.56) [88]<br><br>'Efficacy' = 83.8% OR = 36.07 (95% CI: 14.07, 92.45) [110]
**Arnell**[126] (1983)	Garcia Fernandez[88] (2014)	n = 1; N = 187	NS	Passed CASP quality assessment. No further details.						RR = 4.34 (95% CI: 2.18, 8.63)
Braden – modified by Halfens, "**4-factor model**"[127] (2000)	Zhang[108] (2021); Zimmermann [109] (2018); Garcia-Fernandez[88] (2014); Tayyib[104] (2013)	n = 1; N = 53	Germany; prospective; ICU setting (cardiac surgery); 41.5% female; average age 62 +/- 12.1y; 26 events; EPUAP PI classification used; cut-off ≥2 used.	Passed CASP quality assessment. No further details. [88]	**0.82** [108] **0.85** [104, 109]	**0.31**	**PLR 1.19** **NLR 0.58** [108]		**PPV 0.7** **NPV 0.38** [109]	RR = 1.44 (95% CI: 0.75, 2.75) [88]<br><br>Lower validity for cardiac SICU than Braden [104].
Braden – modified by Halfens, "**extended Braden**"[127] (2000)	Chou[87] (2013)	n = 1; N = 320	Prospective cohort study; hospital in-patients; no PI on admission; mean age 61y; cut-offs ≤15, 18 used; 'Pressure sore incidence' used as reference standard.	Criteria based on QUADAS-II: Rated as fair quality. The study did not report that the groups received comparable interventions and cut-offs were not pre-defined.	*Cut-off ≤15:* **0.07**<br><br>*Cut-off ≤18:* **0.24**	*Cut-off ≤15:* **0.99**<br><br>*Cut-off ≤18:* **0.95**	*Cut-off ≤15:* **PLR 1.21** **NLR 0.16**<br><br>*Cut-off ≤18:* **PLR 0.83** **NLR 0.14**		*Cut-off ≤15:* **PPV 0.55** **NPV 0.86**<br><br>*Cut-off ≤18:* **PPV 0.45** **NPV 0.88**	
Braden – modified by **Kwong**[115] (2005)	Chou[87] (2013)	n = 2; N = 626	Two prospective cohort studies; hospital in-patients; no PI on admission; mean ages 58, 79; cut-off points ≤16, ≤19 used; reference standard NPUAP used.	Criteria based on QUADAS-II: Rated good (n=1) and fair quality (n=1; did not report that groups received comparable interventions, blinding of reference standard unclear). Cut-offs	Cut-off ≤16 (n=1): **0.89**<br><br>Cut-off ≤19 (n=1): **0.89**	Cut-off ≤16: **0.75**<br><br>Cut-off ≤19: **0.62**	Cut-off ≤16: **PLR 0.07** **NLR 0.001**<br><br>Cut-off ≤19: **PLR 0.23** **NLR 0.02**	Cut-off ≤19: **0.74 (0.63, 0.84)**	Cut-off ≤16: **PPV 0.07** **NPV 1.0**<br><br>Cut-off ≤19: **PPV 0.19** **NPV 0.98**	

Tool (develop-ment year)	Reviews' authors (publication year)	n = no. of studies; N = no. of part-icipants	Brief description of included studies	Brief description of included study quality	Sensitivity (95% CI)	Specificity (95% CI)	Likelihood ratios (95% CI)	AUROC (95% CI)	Predictive values (95% CI)	Other outcomes
				were not predefined in either study.						
Cubbin & Jackson – revised, "**Jackson & Cubbin**"[117] (1999)	Chou[87] (2013)	n = 3;<br>N = 865	Three prospective cohort studies; hospital in-patients; mean age ranged between 58 and 62; cut-offs ≤29, 28, 24 used; Sample sizes range between 112 and 534. Reference standards included NPUAP staging system, AHRQ 4-stage criteria and the Stirling Pressure Sore Severity Scale.	Criteria based on QUADAS-II: Rated fair (n=2; sampling method unclear) and good (n=1) quality.<br><br>Blinding of reference standard unclear in all three studies.	*Cut-off ≤29:* **0.83**<br><br>*Cut-off ≤28:* **0.95**<br><br>*Cut-off ≤24:* **0.89**	*Cut-off ≤29:* **0.42**<br><br>*Cut-off ≤28:* **0.82**<br><br>*Cut-off ≤24:* **0.61**	*Cut-off ≤29:* **PLR 0.08** **NLR 0.02**<br><br>*Cut-off ≤28:* **PLR 1.15** **NLR 0.01**<br><br>*Cut-off ≤24:* **PLR 1.03** **NLR 0.08**	*Cut-off ≤29:* **0.72**<br><br>*Cut-off ≤28:* **0.90**<br><br>*Cut-off ≤24:* **0.83**	*Cut-off ≤29:* **PPV 0.07** **NPV 0.98**<br><br>*Cut-off ≤28:* **PPV 0.56** **NPV 0.99**<br><br>*Cut-off ≤24:* **PPV 0.51** **NPV 0.92**	
	Tayyib[104] (2013)	n = 3;<br>N = 519	ICU settings; two prospective observational studies, one longitudinal study; cut-off points ≤29, 28, 24 used.	Not done	*Cut-off ≤29:* NS<br><br>*Cut-off ≤28:* **0.95**<br><br>*Cut-off ≤24:* **0.89**	*Cut-off ≤29:* NS<br><br>*Cut-off ≤28:* **0.82**<br><br>*Cut-off ≤24:* **0.61**				Jackson/Cubbin scale is found to have the highest predictive ability/validity than comparators, in all 3 studies. Comparators: Waterlow, Braden, Douglas, Song and Choi scales.
**COMHON**[128] (2011)	Zhang[108] (2021)	n = 1;<br>N = 2,777	Spain; retrospective; ICU setting; 38.1% female; average age 63 +/- 16y; 154 events; NPUAP PI classification used; cut-off >12 used.	Reported overall, not by scale.	**0.83**	**0.52**	**PLR 1.72** **NLR 0.33**	**0.70**		
**Compton**[129] (2008)	Garcia-Fernandez[88] (2014)	n = 1;<br>N = 698	NS	Passed CASP quality assessment. No further details.						RR = 4.85 (95% CI: 3.66, 6.42)

Tool (develop-ment year)	Reviews' authors (publication year)	n = no. of studies; N = no. of part-icipants	Brief description of included studies	Brief description of included study quality	Sensitivity (95% CI)	Specificity (95% CI)	Likelihood ratios (95% CI)	AUROC (95% CI)	Predictive values (95% CI)	Other outcomes
**Douglas**[130] (1986)	Zhang[108] (2021); Zimmermann [109] (2018); Garcia-Fernandez[88] (2014); Tayyib[104] (2013); Chou[87] (2013); Pancorbo-Hidalgo[110] (2006)	n = 1; N = 112	Korea; prospective, longitudinal; ICU (surgical, internal or neurological) setting; history of PIs unclear; 32.8% female; mean age 62y; 35 events, with follow-up to discharge/moved to other ward/death[110]; 'Panel for the Prediction and Prevention of Pressure Ulcers' classification used[108]; NPUAP classification used[87] (minimum stage I); cut-off ≤18 used.	Passed quality assessment in two reviews that utilised CASP [88, 110].<br><br>Criteria based on QUADAS-II: Rated as good quality. Blinding of reference standard unclear[87].	**1.00**	**0.18**	**PLR 1.22** [A]<br>**NLR 0.00**	**0.79**	**PPV 0.34**<br>**NPV 1.00**	RR = 10.76 (95% CI: 0.70, 166.27) [88]<br><br>'Efficacy' = 43.6%[110]<br><br>Lower validity than Jackson/Cubbin [104]
**DUPA**[131] (1995)	Garcia-Fernandez[88] (2014)	n = 1; N = 85	NS	Passed CASP quality assessment. No further details.						RR = 2.13 (95% CI: 1.21, 3.75)
**Dutch CBO**[132] (1992)	Chou[87] (2013)	n = 1; N = 220	Retrospective study in a nursing home. Mean age 79. Dutch CBO cut-off of ≤10 used. NPUAP staging system used as reference standard.	Criteria based on QUADAS-II: Rated as fair quality. Sampling method unclear, study did not report that interventions were comparable, the scale cut-offs were not pre-defined, it was unclear whether the reference standard was applied to all patients, whether the same reference standard was applied to all patients and whether it was blinded.	0.55	0.75				
**EMINA**[118] (2001)	Pancorbo-Hidalgo[110] (2006)	n = 1; N = 673	Hospital; classification system not reported but minimum stage I; EMINA cut-	All studies considered to be 'valid' through use of CASP.	**0.77**	**0.72**		**0.82**	**PPV 0.17**<br>**NPV 0.98**	'Efficacy' = 71.9%;

**Tool** (develop-ment year)	**Reviews' authors** (publication year)	**n = no. of studies; N = no. of part-icipants**	**Brief description of included studies**	**Brief description of included study quality**	**Sensitivity (95% CI)**	**Specificity (95% CI)**	**Likelihood ratios (95% CI)**	**AUROC (95% CI)**	**Predictive values (95% CI)**	**Other outcomes**
			off ≥4; follow-up period 7 days; average age NS.							OR = 8.24 (95% CI: 4.10, 16.54)
	Zhang[108] (2021)	n = 1;<br>N = 189	Spain; prospective; ICU setting; 32.8% female; average age 59.4 +/- 16.9y; 53 events; NPUAP-EPUAP PI classification used; cut-off point of >10	Reported overall, not by scale.	**0.94**	**0.33**	**PLR 1.41**<br>**NLR 0.17**	**0.64**		
**Fragm-ment**[133] (2002)	Garcia-Fernandez[88] (2014); Chou[87] (2013); Pancorbo-Hidalgo[110] (2006)	n = 1;<br>N = 1,190	Prospective cohort study; hospital in-patients (medical units, surgical units and ICU); no PIs on admission; mean age 61y; cut-off >3 used; NPUAP staging system used as reference standard (minimum stage I); follow-up period 3 weeks/to discharge.	Passed quality assessment in two reviews that utilised CASP[88, 110].<br><br>Criteria based on QUADAS-II: Rated as fair quality, but scale was evaluated on the same population as it was developed on, authors did not report that groups received comparable interventions, cut-offs not pre-defined and blinding of reference standard unclear[87].	**0.62**	**0.85**	**PLR 0.73**<br>**NLR 0.08**	**0.79 (0.75, 0.82)**	**PPV 0.34**<br>**NPV 0.95**	RR = 5.74 (95% CI: 4.40, 7.50) [88]<br><br>RR = 1.6 (95% CI: 1.4, 1.7) per 1 point increase in score[87].
**Gosnell**[134] (1973)	Zhang[108] (2021); Garcia-Fernandez[88] (2014); Chou[87] (2013)	n=1; 230	Iran; prospective; ICU in-patients; no PIs on admission; 56.5% female; average age 60y; 74 events; AHCPR PI classification used; cut-off point of 16	Passed CASP quality assessment[88].<br><br>Criteria based on QUADAS-II: Rated as fair quality. Sampling method and blinding of reference standard unclear[87].	**0.85**	**0.83**	**PLR 4.92**<br>**NLR 0.18**			
**Hatanaka [135]** (2008)	Chou[87] (2013)	n = 1;<br>N = 149	Prospective cohort study; hospital in-patients; no PIs on admission; mean age 72y; cut-off 0.28 (possible range	Criteria based on QUADAS-II: Rated as fair quality. The scale was evaluated in the same population as it was developed on, sampling	**0.73**	**0.70**	**PLR 0.85**<br>**NLR 0.14**	**0.79**	**PPV 0.46**<br>**NPV 0.88**	

**Tool** (develop-ment year)	**Reviews' authors** (publication year)	**n = no. of studies; N = no. of part-icipants**	**Brief description of included studies**	**Brief description of included study quality**	**Sensitivity (95% CI)**	**Specificity (95% CI)**	**Likelihood ratios (95% CI)**	**AUROC (95% CI)**	**Predictive values (95% CI)**	**Other outcomes**
			0-1) used; own criteria used for reference standard.	method unclear, the scale cut-offs were not pre-defined, unclear whether the reference standard was credible and whether it was blinded.						
**HPUR**[136] (2003)	Garcia-Fernandez[88] (2014)	n = 1; N = 54	NS	Passed CASP quality assessment. No further details.						RR = 3.02 (95% CI: 1.26, 7.20)
**Knoll**[137] (1988)	Garcia-Fernandez [88] (2014); Chou[87] (2013); Pancorbo-Hidalgo[110] (2006)	n = 1; N = 60	Prospective cohort study; LTCF; no PIs on admission; mean age 81y; cut-off 12; reference standard unclear, but minimum stage I; follow-up period 28 days.	Passed quality assessment in two reviews that utilised CASP[88, 110].<br><br>Criteria based on QUADAS-II: Rated as fair quality. Unclear whether the scale was evaluated in the same population as it was developed on, unclear whether the reference standard was credible and whether reference standard was blinded[87].	**0.86**	**0.56**	**PLR 1.71**<br>**NLR 0.22**		**PPV 0.63**<br>**NPV 0.82**	RR = 3.47 (95% CI: 1.39, 8.71) [88]<br><br>'Efficacy' = 70.0% OR = 7.71 (95% CI: 2.17, 27.42)[110]
Norton – modified by **Bale**[138] (1995)	Garcia-Fernandez [88] (2014)	n = 1; N = 240	NS	Passed CASP quality assessment. No further details.						RR = 1.35 (95% CI: 0.68, 2.71)
	Chou[87] (2013)	n = 1; N = 79	Prospective cohort study on hospice patients. The scale was reversed so that a higher score represents higher risk. Mean age was 67y. Modified Norton cut-off of >10 used. Torrance Developmental Classification of Pressure Sores used as reference standard.	Criteria based on QUADAS-II: Rated as fair quality. The study didn't report that the groups received comparable interventions, the cut-offs were not pre-defined and blinding of reference standard unclear.	**1.00**	**0.31**	**PLR 3.20**<br>**NLR 0.00**		**PPV 0.04**<br>**NPV 1.00**	

**Tool** (develop-ment year)	**Reviews' authors** (publication year)	**n = no. of studies; N = no. of part-icipants**	**Brief description of included studies**	**Brief description of included study quality**	**Sensitivity (95% CI)**	**Specificity (95% CI)**	**Likelihood ratios (95% CI)**	**AUROC (95% CI)**	**Predictive values (95% CI)**	**Other outcomes**
Norton – modified by **Bienstein**[122] (1991)	Zhang[108] (2021); Tayyib[104] (2013); Chou[87] (2013)	n = 1; N = 53	Germany; prospective; ICU setting (cardiac surgery); 41.5% female; average age 62 +/- 12.1y; 26 events; EPUAP PI classification used; cut-off points ≤25 (and ≤21, 23[87]).	Criteria based on QUADAS-II: Rated as fair quality. The study didn't report that the group received comparable interventions and the cut-offs were not pre-defined[87].	*Cut-off ≤21:* 0.33<br><br>*Cut-off ≤23* 0.41<br><br>*Cut-off ≤25:* 0.58	*Cut-off ≤21:* 0.94<br><br>*Cut-off ≤23* 0.88<br><br>*Cut-off ≤25:* 0.47	*Cut-off ≤21:* PLR 0.92 NLR 0.68<br><br>*Cut-off ≤23:* PLR 0.88 NLR 0.64<br><br>*Cut-off ≤25:* PLR 1.11 NLR 0.87		*Cut-off ≤21:* PPV 0.92 NPV 0.40<br><br>*Cut-off ≤23:* PPV 0.88 NPV 0.42<br><br>*Cut-off ≤25:* PPV 0.70 NPV 0.35	Lower validity for cardiac SICU than Braden[104].
Norton – modified by **Stotts**[139] (1988)	Chou[87] (2013)	n = 1; N = 387	Prospective cohort study on surgical in-patients. Mean age was 53. Modified Norton cut-off of ≤14. Used own criteria for reference standard.	Criteria based on QUADAS-II: Rated as fair quality. The study didn't report that the groups received comparable interventions, cut-offs were not pre-defined, sampling method unclear and blinding of reference standard interpretation unclear.	**0.16**	**0.95**	**PLR 0.67 NLR 0.18**		**PPV 0.4 NPV 0.85**	
Norton – modified by **Ek**[140] (1997)	Pancorbo-Hidalgo[110] (2006)	n = 1; N = 81	Hospital orthopaedics; classification system not reported but minimum stage I; cut-off ≤21 used; follow-up 14 days after surgery/to discharge; average age 82y.	All studies considered to be 'valid' through use of CASP.	**0.71**	**0.44**			**PPV 0.35 NPV 0.78**	'Efficacy' = 52.0% OR = 1.92 (95% CI: 0.69, 5.36)
**NOVA-4**[141] (1999)	Garcia-Fernandez[88] (2014)	n = 1; N = 187	NS	Passed CASP quality assessment. No further details.						RR = 4.72 (95% CI: 1.90, 11.77)
**PSPS**[123] (1987)	Pancorbo-Hidalgo[110] (2006)	n = 1; N = 1,244	Hospital orthopaedics; classification system and minimum PI stage not reported; PSPS cut-off >6; follow-up period 3 weeks; average age NS.	All studies considered to be 'valid' through use of CASP.	**0.89**	**0.76**			**PPV 0.14 NPV 0.99**	'Efficacy' = 76.6% OR = 25.62 (95% CI: 10.73, 61.20)

Tool (develop-ment year)	Reviews' authors (publication year)	n = no. of studies; N = no. of part-icipants	Brief description of included studies	Brief description of included study quality	Sensitivity (95% CI)	Specificity (95% CI)	Likelihood ratios (95% CI)	AUROC (95% CI)	Predictive values (95% CI)	Other outcomes
**RAPS**[142] (2002)	Zhang[108] (2021); Zimmermann [109] (2018)	n = 1; N = 122	Turkey; prospective; ICU setting; 57.4% female; average age 56.5 +/- 18.6y; 31 events; NPUAP PI classification used; cut-off point of ≥27	Reported overall, not by scale.	**0.74**	**0.32**	**PLR 1.09** **NLR 0.81**	**0.5**	**PPV 0.387** **NPV 0.913**	Studies that compared the predictive capacity of specific and generic scales (according to setting) presented significant variation. Sensitivity variation of all scales was high, ranging from 60% to 100%. However, in specificity values, the ICU specific scales showed higher variations (from 61% to 86%), when compared with generic scales (from 5% to 69%)[109].
	Garcia-Fernandez[88] (2014); Chou[87] (2013); Pancorbo-Hidalgo[110] (2006)	n = 1; N = 488	Prospective cohort; hospital in-patients (hospital medical, surgical, orthopaedic and geriatric units); no PIs on admission; mean age 70y; cut-off ≤36; own criteria used for reference standard; minimum stage I; follow-up period 12 weeks.	Passed quality assessment in two reviews that utilised CASP[88, 110]. Criteria based on QUADAS-II: Rated as poor quality. The scale was evaluated in the same population as it was developed on, the test cut-	**0.57**	**0.58**	**PLR 0.19** **NLR 0.10**		**PPV 0.14** **NPV 0.92**	RR = 1.71 (95% CI: 1.03, 2.85) [88] 'Efficacy' = 57.4% OR = 1.82 (95% CI: 1.06, 3.11)[110]

Tool (develop-ment year)	Reviews' authors (publication year)	n = no. of studies; N = no. of part-icipants	Brief description of included studies	Brief description of included study quality	Sensitivity (95% CI)	Specificity (95% CI)	Likelihood ratios (95% CI)	AUROC (95% CI)	Predictive values (95% CI)	Other outcomes
				offs were not pre-specified, the study did not report that groups received comparable interventions and attrition was high. Sampling method and whether the reference standard was blinded were unclear.						
**SS scale**[143] (2008)	Zhang[108] (2021); Zimmermann [109] (2018); Garcia-Fernandez[88] (2014); Tayyib[104] (2013)	n = 1; N = 253	Indonesia; prospective; ICU setting; 37.5% female; 72 events; NPUAP PI classification used; cut-off point >4	Passed CASP quality assessment. No further details.[88]	**0.81**	**0.83**	**PLR 4.70** **NLR 0.23**	**0.89**	**PPV 0.65** **NPV 0.91**	RR = 7.63 (95% CI: 4.52, 12.89) [88]
**Sunderland** [144] (1995)	Zhang[108] (2021); Zimmermann [109] (2018)	n=1; 90	Portugal; prospective; ICU setting; 36.7% female; average age 70y; 15 events; NPUAP-EPUAP PI classification used; cut-off point of 28	Reported overall, not by scale.	**0.6**	**0.87**	**PLR 4.50** **NLR 0.46**	**0.86**	**PPV 0.47** **NPV 0.92**	
	Garcia-Fernandez[88] (2014)	n = 1; N = 15	NS	Passed CASP quality assessment. No further details.						RR = 1.25 (95% CI: 0.22, 7.08)
**TNH-PUPP**[145] (2011)	Chou[87] (2013)	n = 1; N = 165	Prospective cohort study in hospital general wards, critical care or emergency departments. History of PI unclear; existing PI (%) unclear. Mean age 68y. TNH-PUPP cut-off 3. Reference standard unclear.	Criteria based on QUADAS-II: Rated as fair quality. Sampling method unclear, the study did not report that interventions were comparable, the scale cut-offs were not pre-defined, it was unclear whether the reference standard was	**0.86**	**0.73**	**PLR 0.13** **NLR 0.01**	**0.90 (0.82, 0.99)**	**PPV 0.13** **NPV 0.99**	

**Tool** (develop-ment year)	**Reviews' authors** (publication year)	**n = no. of studies; N = no. of part-icipants**	**Brief description of included studies**	**Brief description of included study quality**	**Sensitivity (95% CI)**	**Specificity (95% CI)**	**Likelihood ratios (95% CI)**	**AUROC (95% CI)**	**Predictive values (95% CI)**	**Other outcomes**
				credible and whether it was blinded.						
**Watkinson** [146] (1997)	Garcia-Fernandez[88] (2014)	n = 1; N = 185	NS	Passed CASP quality assessment. No further details.						RR = 34.88 (95% CI: 2.12, 574.39)
Bayesian Network: **ML Kaewprag [2]**[147] (2017)	Qu[103] (2022)	n = 1; N = 7,717	NS; Sensitivity and specificity calculated by umbrella review team from raw data	QUADAS-II: Low overall risk of bias.	**0.64 (0.60, 0.68)** n = 590	**0.81 (0.80, 0.82)** n = 7127				
Bayesian Network: **ML Ladios Martin**[148] (2020)	Qu[103] (2022)	n = 1; N = 1,769	NS; Sensitivity and specificity calculated by umbrella review team from raw data	QUADAS-II: High overall risk of bias. High risk of bias and applicability concerns in reference standard domain.	**0.04 (0.09, 0.12)** n = 68	**0.93 (0.92, 0.94)** n = 1701				
**LOS** model: **ML Kim [2]**[149] (2006)	Qu[103] (2022)	n = 1; N = 2,347	NS; Sensitivity and specificity calculated by umbrella review team from raw data	QUADAS-II: Low overall risk of bias.	**0.92 (0.84, 0.97)** n = 84	**0.67 (0.65, 0.69)** n = 2263				

CI – confidence interval; AUROC – area under the receiver operating characteristic curve; NS – not stated, CASP – Critical Appraisal Skills Programme; RR – risk ratio; efficacy – percentage of correctly classified patients; OR – odds ratio; PI – pressure injury; (S)ICU – (surgical) intensive care unit; QUADAS – Quality Assessment of Diagnostic Accuracy Studies; EPUAP – European Pressure Ulcer Advisory Panel; NPUAP – National Pressure Ulcer Advisory Panel; AHCPR – Agency for Health Care Policy and Research; AHRQ – Agency for Healthcare Research and Quality; PLR – positive likelihood ratio; NLR – negative likelihood ratio; PPV – positive predictive value; NPV – negative predictive value; LTC(F) – long-term care (facility); ML – machine learning; LOS – abbrev. not defined within review.
[A] re-calculated due to discrepancy between reviews. * Three reviews [87, 88, 98] conducted inappropriate statistical syntheses, therefore for tools included in those syntheses, the results of other individual studies of such tools may also be presented within this table.

Table F. Results from all 11 included systematic reviews evaluating clinical effectiveness

Review author (publication year)	Tools included	Setting of included studies; study design; sample size	Included outcomes	Brief description of study quality	Relevant results from included studies
Lovegrove[94] (2021)	Braden; Maelor score; Norton; Ramstadius; Waterlow	Acute care hospital n=1, inpatient units n=1, ICU n=1, internal medicine and oncology wards n=1;<br><br>Design: cross-sectional survey n=2, RCT n=1, observational inter-rater reliability n=1;<br><br>Sample size 45 to 1231	PI risk scores; PI incidence; PI preventative interventions; interrater reliability	RoB assessed using JBI tools or analytical cross-sectional study appraisal checklist. The RCT was judged as high quality. Of the remaining studies, two were judged as high quality and one as moderate quality; inclusion criteria not clearly stated and no strategies to deal with confounding.	• The Braden scale had the highest ICC across two ICUs (0.72 and 0.84), followed by subjective assessments (0.51 and 0.71) then the Waterlow score (0.36 and 0.51) (Kottner and Dassen 2010[150]).<br>• There were no differences in patient management ('pressure care plan' and use of a special mattress) based on PI risk assessment method (clinical judgement, Ramstadius tool or Waterlow score). PI incidence difference between groups not significant (p=0.44) (Webster 2011[151]).<br>• A hospital that used the Maelor scale reported a higher rate of PI preventative strategies than a site that used nurses' clinical judgement (Moore 2015[152]).<br>• 33% of nursing assessments differed from those of the computer-generated Norton score. Of the patients assessed as being at high risk of PI by the Norton scale, 64% were repositioned 2 or 3 hourly, 7% were repositioned 4 or 5 hourly, 4% were repositioned hourly and 7% were not repositioned at all (Voz 2011[153]).
Mehicic[96] (2024)	Braden	All studies were based in ICU settings;<br><br>Design: all prospective studies n=9;<br><br>Sample size 3 to 4137	Internal consistency (n=2); interrater reliability (n=6); measurement error (n=3); convergent validity (n=3)	RoB assessed using COSMIN RoB checklist.<br><br>All studies assessing internal consistency or measurement error scored 'Very Good' for all relevant criteria.<br><br>Half (n=3) the studies assessing interrater reliability scored 'Very Good' for all relevant criteria; one study scored 'Doubtful' for all relevant	• **Internal consistency.** Two studies (Lima-Serrano 2018[154] and Adibelli 2019[155]) reported Cronbach's alpha ranging from poor (0.43) to good (0.85).<br>• **Interrater reliability**. Only four (4/6) studies reported intraclass correlation, ranging from 0.66 to 0.96 for Braden sum score. One study (Veiga 2022) reported weighted kappa = 0.17 and another reported kappa ranging from 0 to 0.86 (not weighted) (Simao 2013[156]).<br>• **Measurement error.** Standard error of measurement (SEM) was around 2 points of the

**Review author** (publication year)	**Tools included**	**Setting of included studies; study design; sample size**	**Included outcomes**	**Brief description of study quality**	**Relevant results from included studies**
				criteria, one study scored 'Inadequate' for 4/7 criteria, and the last study scored 'Doubtful' for 2/5 criteria and 'Inadequate' for 1/5 criteria.<br><br>Two studies assessing convergent validity scored 'Very Good' for all criteria; the other study scored 'Doubtful' for 3/4 criteria.	sum score (1.83 and 1.67–1.64) in two studies (Fulbrook 2016[157] and Kottner 2010[150], respectively), and a slightly smaller SEM of 1.31 was reported by the third study (Veiga 2022[158]).<br>• **Convergent validity.** The Braden scale was compared with four comparator tools: the Cubbin-Jackson scale (Delawder 2021[159]), the COHMON Index and Norton scale (Fulbrook 2016[157]), and the Waterlow score (Fulbrook 2016[157]; Kottner 2010[150]).<br>• One study found weak correlation between the sum scores of the Braden scale and Waterlow scale (r=0.22) (Fulbrook 2016[157]), whereas another study found strong correlations between the two scales (r=0.72,0.71) (Kottner 2010[150]).<br>• Fulbrook 2016[157] reported strong correlations between the Braden and Norton sum scores (r=0.77), and between the Braden and the COMHON Index (r=0.70).<br>• Delawder 2021[159] reported strong correlation between the Braden and the Cubbin-Jackson scale (r=0.80)
Moore[97] (2019)	Braden; Waterlow; Ramstadius	Military hospital n=1, internal medicine and oncology wards n=1;<br><br>Design: RCT n=1, cluster randomised trial n=1;<br><br>Sample sizes 286 and 1231	PI incidence; severity of PIs	RoB assessed using Cochrane tool (Higgins (2011)).<br>Both studies at high RoB due to blinding issues. One study at RoB also due to baseline imbalance and incorrect analyses.	• No differences in PI incidence when using Braden scale or clinical judgement (Braden vs. clinical judgement+training, RR 0.97, 95% CI 0.53, 1.77; Braden vs clinical judgement RR 1.43, 95% CI 0.77, 2.68) (Saleh 2009[160]).<br>• No difference in PI incidence when using a risk assessment tool compared to clinical judgement (RR 1.10, 95% CI 0.68, 1.81 and RR 0.79, 95% CI 0.46, 1.35, for Waterlow and Ramstadius respectively) (Webster 2011[151]).<br>• No difference in PI severity based on risk assessment tools vs. clinical judgement (Webster 2011[151]).

**Review author** (publication year)	**Tools included**	**Setting of included studies; study design; sample size**	**Included outcomes**	**Brief description of study quality**	**Relevant results from included studies**
Gaspar[89] (2019)	Waterlow; Ramstadius	Internal medical and oncology wards n=1;<br><br>Design: RCT n=1<br><br>Sample size 1231	PI incidence	RoB assessed using Evidence-Based Librarianship Critical Appraisal checklist. Overall validity of the eligible study was 95.83%. The study scored 87.5% for the population domain.	• The incidence of HAPI was similar between the groups (6.8% vs. 7.5% vs. 5.4%, p=0.44 for Waterlow, Ramstadius and clinical judgement respectively) (Webster 2011[151]).
Chou[87] (2013)	Norton modified by Bale; Braden; Waterlow; Ramstadius	Hospital n=2, hospice n=1;<br><br>Design: non-randomised n=1, cluster randomised trial n=1, RCT n=1;<br><br>Sample size 240 to 1231	PI incidence, severity of PIs; PI preventative interventions	RoB assessed with criteria consistent with AHRQ Methods Guide for Effectiveness and Comparative Effectiveness Reviews. One RCT was rated as good quality and the other as poor due to randomisation and blinding issues. The cohort study was rated as poor; there were blinding issues and confounding was not investigated.	• No difference in PI incidence when using a risk assessment tool compared to clinical judgement (RR 1.10, 95% CI 0.68, 1.81 and RR 0.79, 95% CI 0.46, 1.35, for Waterlow and Ramstadius respectively) (Webster 2011[151]).<br>• The modified version of the Norton scale with use of preventive interventions is associated with lower risk of PIs compared with clinical judgment (RR 0.11, 95% CI 0.03, 0.46) (Bale 1995[138]).<br>• No difference in risk of PIs when one of three interventions was used (22% vs. 22% vs. 15%, p=0.38 for nurse training+mandatory Braden scale, nurse training+optional Braden scale and no training respectively) (Saleh 2009[160]).
Pancorbo-Hidalgo[110] (2005)	Norton; Norton modified by Bale; Norton modified by Ek 97	Hip fracture inpatients n=1, palliative care/hospice n=1, neurosurgery, general medicine, orthopaedic, and oncology units n=1;<br><br>Design: prospective controlled (contemporaneous controls) n=1, before-and-after n=1;<br><br>Sample size: 124 to 223	PI incidence; PI preventative interventions	RoB assessed using CASP Guide for clinical trials or<br>the critical assessment guide developed for the clinical practice guide for PI assessment and prevention for cohort studies (Rycroft-Malone & McInness (2000)). Studies excluded if considered not to be valid.	• Compared a strategy that gave high risk patients (based on modified Norton score) a risk alarm sticker to standard care. No significant difference between the groups in the incidence of PIs (OR 1.18, 95% CI 0.52, 2.66) (Gunningberg 1999[161]).<br>• The modified version of the Norton scale with use of pressure-reducing mattresses is associated with lower risk of PIs compared with clinical judgment (decrease in PI incidence in the Norton scale group of 19.8%, 95% CI 12.2, 27.4). More patients in the Norton group were given then mattresses (Bale 1995[138]).<br>• Compared the Norton scale with training to standard care. There was a significant difference in the number of preventative interventions

**Review author** (publication year)	**Tools included**	**Setting of included studies; study design; sample size**	**Included outcomes**	**Brief description of study quality**	**Relevant results from included studies**
					(18.96 vs. 10.75, for Norton and usual care respectively) (Hodge 1990[162]).
Health Quality Ontario[91] (2009)	Norton; Norton modified by Bale; Norton modified by Ek 97	Hip fracture inpatients n=1, palliative care/hospice n=1, neurosurgery, general medicine, orthopaedic, and oncology units n=1;<br><br>Design: prospective controlled (contemporaneous controls) n=1, before-and-after n=1;<br><br>Sample size 124 to 223	PI incidence; PI preventative interventions	RoB assessment criteria name not given. Two studies met 6/8 and one study met all quality assessment requirements. In the studies that didn't meet all requirements, there were blinding and loss to follow-up issues. One study used a version of the Norton scale that was not validated.	• Compared a strategy that gave high risk patients (based on modified Norton score) a risk alarm sticker to standard care. No significant difference between the groups in the incidence of PIs (Gunningberg 1999[161]).<br>• Compared a strategy where patients received a pressure support system allocated according to the modified Norton scale to one where the nurse chose whether to give a special mattress. Using the scale significantly reduced the incidence of PIs (22.4% vs. 2.5%, p<0.001) (Bale 1995[138]).<br>• Compared the Norton scale with training to standard care. There was a significant difference in the number of preventative interventions (18.96 vs. 10.75, for Norton and usual care respectively). Interventions were used earlier for Norton vs. usual care (on day 1, 61% vs. 50%, p<0.002). No significant difference in the incidence of PIs between the groups (Hodge 1990[162]).
Lovegrove[95] (2018)	Braden; Cubbin & Jackson; modified Norton; Ramstadius; Waterlow	Hospital or acute care n=20;<br><br>Design: cross-sectional n=15, observational n=2, RCT n=1, pre-test post-test n=1, prospective cohort=1;<br><br>Sample size not clearly reported	Level of risk in relation to the prescription of PI preventive interventions	RoB assessed using JBI tools. The RCT, eleven cross-sectional studies, the cohort and the observational studies were rated as high quality (n=15). Four cross-sectional and the pre-test post-test studies were rated as moderate quality (n=5). Reporting of the prescription of interventions for PI was unclear.	• Limited mention of specific risk assessment tools.<br>• Seven studies reported PI risk assessment, risk status and interventions implemented for patients identified as being at-risk in some way and 13 linked it to preventative intervention prescription and implementation in some way.<br>• No studies linked PI risk assessment to preventative intervention prescription alone.
Baris[84] (2015)	Braden	ICU n=12, general hospital n=2, surgical n=1,	PI incidence; reliability of Braden scale	No RoB assessment	• PIs developed in 64% of patients with a Braden score ≤12 (Bakanoglu 2010[163]).

**Review author** (publication year)	**Tools included**	**Setting of included studies; study design; sample size**	**Included outcomes**	**Brief description of study quality**	**Relevant results from included studies**
		orthopaedics and traumatology clinic n=1;<br><br>Design: descriptive n=11, experimental n=5;<br><br>Sample size 22 to 422			• PIs developed in 20.8% of patients with a Braden score ≥13 and in 46.6% of patient with a Braden score ≤12. The difference was statistically significant (Oguz 1998[164]).<br>• Braden scale had a reliability coefficient of 0.95 (Oguz 1998[164]).<br>• Seven studies regarded the Braden scale as 'valid and reliable'.
Kottner[93] (2009)	Waterlow; Waterlow 12-item	Hospital n=6, community n=1, unclear n=2;<br><br>Design: interrater reliability n=7 studies, intra-rater reliability n=1, unclear n=1;<br><br>Sample size 1 to 52	Interrater reliability; intra-rater reliability	Used own RoB assessment criteria. Two studies rated as high quality. Issues with the other studies include poor description of raters, subjects, methods, procedures and results, non-independent scoring, non-representative results and inappropriate statistical methods.	• Waterlow inter-rater reliability: p^a within one point=0.6 (Dealey 1989[165])<br>• Waterlow inter-rater reliability: p^a=0.25; p^a within one point=0.5; range of differences 0-11 (Edwards 1995[166])<br>• Waterlow inter-rater reliability: p^a=0; p^a within one point=0.11; p^a within two points=0.33; p^a within 3 points=0.44; p^a within 4 points=0.56 (Watkinson 1996[167])<br>• Waterlow inter-rater reliability: r=0.99 (Pang and Wong 1998[114])<br>• Waterlow 12-item inter-rater reliability: p^a=0.21-0.57; p^a within 1 point=0.29-0.72; p^a within 2 points=0.5-0.86; range of differences 0-15 (Cook 1999[168])<br>• Waterlow inter-rater reliability: mean p^a=78.1; range of differences 0-16 (Hale 1999[169])<br>• Waterlow inter-rater reliability: p^a=0.11; p^a within one point=0.27; p^a within 2 points 0.4; range of differences 0-24 (Kelly 2005[170])<br>• Waterlow intra-rater reliability: p^a=55.1; ICC=0.97 (95% CI 0.94, 0.98) (Hale 1999[169])<br>• Waterlow type of comparison unclear: ICC=0.95 (Jalali and Rezaie 2005[171])
Tayyib[104] (2013)	Braden; Waterlow; SS scale	ICU n=2; ICU cardiac surgery n=1;	Reliability of PI scales; interrater reliability	No RoB assessment	• Braden scale demonstrated high reliability (Pearson's r: 0.83-0.99) (Lewicki 2000[172])

**Review author** (publication year)	**Tools included**	**Setting of included studies; study design; sample size**	**Included outcomes**	**Brief description of study quality**	**Relevant results from included studies**
		Design: prospective cohorts n=2, observational n=1;<br><br>Sample size 105 to 3027			• Braden scale has high inter-rater reliability value compared to Waterlow scale (Kottner and Dassen 2010[150]).<br>• S.S. showed high inter-rater reliability (r = 1) (Sanada 2008[143]).

AHRQ – Agency for Healthcare Research; CASP – Critical Appraisal Skills Checklist; COSMIN – COnsensus-based Standards for the selection of health Measurement INstruments; ICU – Intensive Care Unit; JBI – Joanna Briggs Institute; RCT – Randomised Controlled Trial; RoB – Risk of Bias; S.S. – Suriadi Sanada Scale

[A]Proportion of agreement.

References

1. Shea BJ, Reeves BC, Wells G, Thuku M, Hamel C, Moran J, et al. AMSTAR 2: a critical appraisal tool for systematic reviews that include randomised or non-randomised studies of healthcare interventions, or both. BMJ. 2017;358:j4008. doi: 10.1136/bmj.j4008.
2. Alves AGP, Borges JWP, Brito MdA. Assessment of risk for pressure ulcers in intensive care units: an integrative review. Revista de Pesquisa Cuidado é Fundamental Online. 2014;6(2):793-804. doi: 10.9789/2175-5361.2014.v6i2.793-804.
3. Anthony D, Parboteeah S, Saleh M, Papanikolaou P. Norton, Waterlow and Braden scores: a review of the literature and a comparison between the scores and clinical judgement. J Clin Nurs. 2008;17(5):646-53. doi: 10.1111/j.1365-2702.2007.02029.x.
4. Cavalcante T, Queiroz A, Moura E, Furtado L, Kelly da Silva Monte B. UPDATING PF THE ASSISTANCE PROTOCOL FOR PRESSURE ULCER PREVENTION: EVIDENCE BASED PRACTICE. Journal of Nursing UFPE online. 2016;10:1498. doi: 10.5205/reuol.7057-60979-3-SM-1.1003sup201618.
5. Charalambous C, Koulori A, Vasilopoulos A, Roupa Z. Evaluation of the Validity and Reliability of the Waterlow Pressure Ulcer Risk Assessment Scale. Med Arch. 2018;72(2):141-4. doi: 10.5455/medarh.2018.72.141-144.
6. de Laat EH, Schoonhoven L, Pickkers P, Verbeek AL, van Achterberg T. Epidemiology, risk and prevention of pressure ulcers in critically ill patients: a literature review. Journal of wound care. 2006;15(6):269-75. doi: 10.12968/jowc.2006.15.6.26920.
7. do Egito Cavalcanti de Farias A, Bezerra de Queiroz R. RISK FACTORS FOR THE DEVELOPMENT OF PRESSURE INJURY IN THE ELDERLY: INTEGRATIVE REVIEW. Revista de Pesquisa: Cuidado e Fundamental. 2022;14(1). doi: 10.9789/2175-5361.rpcfo.v14.11423.
8. Feuchtinger J, Halfens RJG, Dassen T. Pressure ulcer risk factors in cardiac surgery: A review of the research literature. Heart and Lung: Journal of Acute and Critical Care. 2005;34:375-85. doi: 10.1016/j.hrtlng.2005.04.004.
9. García-Fernández FP, Agreda JJ, Verdú J, Pancorbo-Hidalgo PL. A new theoretical model for the development of pressure ulcers and other dependence-related lesions. J Nurs Scholarsh. 2014;46(1):28-38. Epub 2013/10/11. doi: 10.1111/jnu.12051.
10. Garrubba M, Melder A. Effectiveness of the Braden risk screening tool for pressure injuries: systematic review. Centre for Clinical Effectiveness, Monash Innovation and Quality, Monash Health, Melbourne, Australia. 2017.
11. Kelechi TJ, Arndt JV, Dove A. Review of pressure ulcer risk assessment scales. J Wound Ostomy Continence Nurs. 2013;40(3):232-6. doi: 10.1097/WON.0b013e31828f2049.
12. Keller BP, Wille J, van Ramshorst B, van der Werken C. Pressure ulcers in intensive care patients: a review of risks and prevention. Intensive Care Med. 2002;28(10):1379-88. Epub 2002/09/07. doi: 10.1007/s00134-002-1487-z.
13. Ladd S, Ekanem U, Caffrey J. 540 A Systematic Review of Pressure Ulcers in Burn Patients: Risk Factors, Demographics, and Treatment Modalities. Journal of Burn Care & Research. 2018;39:S233-S4. doi: 10.1093/jbcr/iry006.443.
14. Lepisto M, Eriksson E, Hietanen H, Lepisto J, Lauri S. Developing a pressure ulcer risk assessment scale for patients in long-term care. Ostomy/wound management. 2006;52(2):34-46.
15. Mendes Coqueiro J, Silva Brito R. MULTIPLE RISK FACTORS AND PREVENTIVE STRATEGIES OF PRESSURE ULCERS: SYSTEMATIC REVIEW. Journal of Nursing UFPE / Revista de Enfermagem UFPE. 2013;7(10):6215-22. doi: 10.5205/reuol.4397-36888-6-ED.0710esp201321.
16. Michel JM, Willebois S, Ribinik P, Barrois B, Colin D, Passadori Y. As of 2012, what are the key predictive risk factors for pressure ulcers? Developing French guidelines for clinical practice. Annals of Physical and Rehabilitation Medicine. 2012;55:454-65. doi: 10.1016/j.rehab.2012.08.003.
17. Ming L, Qin G, Haobin Y, Fong P. Systematic review of pressure ulcer risk assessment scales for using in ICU patients. Chinese Nursing Research. 2012;26(1):1-4.
18. Mordiffi SZ, Tho PC, Kent B, Phillips N. Evaluating the effects of using the mobility assessment sub-scale within the Braden Scale on pressure ulcer incidence and preventive

interventions in adult acute care settings: A systematic review. JBI Libr Syst Rev. 2010;8(16 Suppl):1-13. doi: 10.11124/01938924-201008161-00006.
19. Mortenson WB, Miller WC. A review of scales for assessing the risk of developing a pressure ulcer in individuals with SCI. Spinal Cord. 2008;46:168-75. doi: 10.1038/sj.sc.3102129.
20. Nadeem A, Healee D. UTILITY OF THE WATERLOW SCALE IN ACUTE CARE SETTINGS: A LITERATURE REVIEW. Kai Tiaki Nursing Research. 2021;12(1):44-8.
21. O'Tuathail C, Taqi R. Evaluation of three commonly used pressure ulcer risk assessment scales. Br J Nurs. 2011;20(6):S27-8, S30, S2 Passim. doi: 10.12968/bjon.2011.20.Sup2.S27.
22. Rodriguez Torres MC, Soldevilla Agreda JJ. Clinical judgement or assessment scales to identify patients at risk of developing pressure ulcers? Gerokomos. 2007;18:48-51.
23. Almeida ÍL, Garces T, Oliveira G, Moreira TM. Pressure injury prevention scales in intensive care units: an integrative review. 2020;21:e42053. doi: 10.15253/2175-6783.20202142053.
24. Santos CT, Almeida Mde A, Oliveira MC, Victor MA, Lucena Ade F. Development of the nursing diagnosis risk for pressure ulcer. Revista Gaucha de Enfermagem. 2015;36(2):113-21. doi: 10.1590/1983-1447.2015.02.49102.
25. Šáteková L, Žiaková K. VALIDITY OF PRESSURE ULCER RISK ASSESMENT SCALES: REVIEW. Central European Journal of Nursing & Midwifery. 2014;5(2):85-92.
26. Shahin E, Dassen T, Halfens R. Predictive validity of pressure ulcer risk assessment tools in intensive care patients. Connect: The World of Critical Care Nursing. 2007;5:75-9. doi: 10.1891/1748-6254.5.3.75.
27. Smet S, de Graaf A, Bernaerts K, Casaer MP, Beeckman D. The Belgian pressure ulcer risk assessment project: Is assessing mobility and skin status a more accurate, reliable, and feasible approach to assess pressure ulcer risk in hospitalised patients? Int Wound J. 2019;16(6):1577-8. Epub 2019/10/13. doi: 10.1111/iwj.13240.
28. Solati S, Ahmadinezhad M, Alizadeh S. Predictive values of Braden and waterlow scales to assess the risk of pressure ulcer : Review article. Int Electron J Med. 2016;5(2):12-7.
29. Taylor KJ, Bryant R, Boarini J. Assessment tools for the identification of patients at risk for the development of pressure sores: a review. J Enterostomal Ther. 1988;15(5):201-5. doi: 10.1097/00152192-198809000-00030.
30. Tran JP, McLaughlin JM, Li RT, Phillips LG. Prevention of Pressure Ulcers in the Acute Care Setting: New Innovations and Technologies. Plast Reconstr Surg. 2016;138(3 Suppl):232s-40s. doi: 10.1097/prs.0000000000002644.
31. Tschannen D, Anderson C. The pressure injury predictive model: A framework for hospital-acquired pressure injuries. Journal of clinical nursing. 2020;29:1398-421. doi: 10.1111/jocn.15171.
32. Walsh B, Dempsey L. Investigating the reliability and validity of the waterlow risk assessment scale: a literature review. Clin Nurs Res. 2011;20(2):197-208. Epub 2010/11/15. doi: 10.1177/1054773810389809.
33. Xu FR, Shi ZY, Yang FR. Risk assessment tools for pressure injury in intensive care patients: a review. Connect: The World of Critical Care Nursing. 2018;12(1):16-9.
34. Alderden J, Rondinelli J, Pepper G, Cummins M, Whitney J. Risk factors for pressure injuries among critical care patients: A systematic review. International journal of nursing studies. 2017;71:97-114. doi: 10.1016/j.ijnurstu.2017.03.012.
35. Barbosa da Silva J, Soares Rodrigues MC. Pressure ulcers in individuals with spinal cord injury: risk factors in neurological rehabilitation. Rev Rene. 2020;21(1):1-9. doi: 10.15253/2175-6783.20202144155.
36. Di Prinzio MF, Argento FJ, Barbalaco L, Cazurro ME, Pereyra A, Sanchez Correa C, et al. [Risk factors for the development and recurrence of pressure ulcers in patients with spinal cord injury: A systematic review.]. Revista de la Facultad de Ciencias Medicas de Cordoba. 2019;76(4):242-56. doi: 10.31053/1853.0605.v76.n4.24906.

37. Haisley M, Sorensen JA, Sollie M. Postoperative pressure injuries in adults having surgery under general anaesthesia: systematic review of perioperative risk factors. The British journal of surgery. 2020;21. doi: 10.1002/bjs.11448.
38. Ham W, Schoonhoven L, Schuurmans MJ, Leenen LPH. Pressure ulcers from spinal immobilization in trauma patients: A systematic review. Journal of Trauma and Acute Care Surgery. 2014;76:1131-41. doi: 10.1097/TA.0000000000000153.
39. Lima AR, Ribeiro Palmer C, Nogueira PC. RISK FACTORS AND PREVENTIVE INTERVENTIONS FOR PRESSURE INJURIES IN CANCER PATIENTS. Revista Estima. 2021;19:1-13. doi: 10.30886/estima.v19.1005_IN.
40. Lima Serrano M, González Méndez MI, Carrasco Cebollero FM, Lima Rodríguez JS. Risk factors for pressure ulcer development in Intensive Care Units: Systematic review. Medicina intensiva. 2017;41(6):339-46. doi: 10.1016/j.medin.2016.09.003.
41. Marin J, Nixon J, Gorecki C. A systematic review of risk factors for the development and recurrence of pressure ulcers in people with spinal cord injuries. Spinal Cord. 2013;51:522-7. doi: 10.1038/sc.2013.29.
42. Rao AD, Preston AM, Strauss R, Stamm R, Zalman DC. Risk Factors Associated With Pressure Ulcer Formation in Critically Ill Cardiac Surgery Patients: A Systematic Review. Journal of wound, ostomy, and continence nursing : official publication of The Wound, Ostomy and Continence Nurses Society / WOCN. 2016;43(3):242-7. doi: 10.1097/WON.0000000000000224.
43. Reenalda J, Jannink M, Nederhand M, M IJ. Clinical use of interface pressure to predict pressure ulcer development: a systematic review. Assistive technology : the official journal of RESNA. 2009;21:76-85. doi: 10.1080/10400430903050437.
44. Shi C, Dumville JC, Cullum N. Skin status for predicting pressure ulcer development: A systematic review and meta-analyses. International journal of nursing studies. 2018;87:14-25. doi: 10.1016/j.ijnurstu.2018.07.003.
45. Siping S, Qixia J, Xiaoqing L. Risk factors of intraoperative acquired pressure injury: A systematic review and meta-analysis. Clinical Focus. 2022;37(3):211.
46. Wynn M, Stephens M, Pradeep S, Kennedy R. Risk factors for the development and evolution of deep tissue injuries: A systematic review. Journal of tissue viability. 2022. doi: 10.1016/j.jtv.2022.03.002.
47. Zhang X, Haiju L, Yan Z, Yongli L, JunJin X, Chunxia Y, et al. Prevalence and Risk Factors of Postoperative Pressure Ulcers: A Systematic Review and Meta-analysis of Diagnostic Test. Medicinal Plant. 2022;13(6).
48. Bulfone G, Bressan V, Morandini A, Stevanin S. Perioperative Pressure Injuries: A Systematic Literature Review. Advances in skin & wound care. 2018;31:556-64. doi: 10.1097/01.ASW.0000544613.10878.ed.
49. Chung ML, Widdel M, Kirchhoff J, Sellin J, Jelali M, Geiser F, et al. Risk Factors for Pressure Injuries in Adult Patients: A Narrative Synthesis. International Journal of Environmental Research and Public Health. 2022;19. doi: 10.3390/ijerph19020761.
50. Chung ML, Widdel M, Kirchhoff J, Sellin J, Jelali M, Geiser F, et al. Risk factors for pressure ulcers in adult patients: A meta-analysis on sociodemographic factors and the Braden scale. Journal of clinical nursing. 2022;21. doi: 10.1111/jocn.16260.
51. Coleman S, Gorecki C, Nelson EA, Closs SJ, Defloor T, Halfens R, et al. Patient risk factors for pressure ulcer development: Systematic review. International Journal of Nursing Studies. 2013;50:974-1003. doi: 10.1016/j.ijnurstu.2012.11.019.
52. Dube A, Sidambe V, Verdon A, Phillips E, Jones S, Lintern M, et al. Risk factors associated with heel pressure ulcer development in adult population: A systematic literature review. Journal of Tissue Viability. 2022;31(1):84-103. doi: 10.1016/j.jtv.2021.10.007.
53. Ferris A, Price A, Harding K. Pressure ulcers in patients receiving palliative care: A systematic review. Palliative Medicine. 2019;33:770-82. doi: 10.1177/0269216319846023.

54. Floyd NA. Effectiveness of Pressure Ulcer Protocols with the Braden Scale for Elderly Patients in the Intensive Care Unit: A Systematic Review: Walden University; 2018.
55. Gélis A, Dupeyron A, Legros P, Benaïm C, Pelissier J, Fattal C. Pressure ulcer risk factors in persons with SCI: Part I: Acute and rehabilitation stages. Spinal Cord. 2009;47(2):99-107. Epub 2008/09/02. doi: 10.1038/sc.2008.107.
56. Gelis A, Dupeyron A, Legros P, Benaim C, Pelissier J, Fattal C. Pressure ulcer risk factors in persons with spinal cord injury part 2: the chronic stage. Spinal Cord. 2009;47(9):651-61. doi: 10.1038/sc.2009.32.
57. Liu ZF, Meng J, Jing N, Liu XY. Effects of predictive nursing interventions on pressure ulcer in older bedridden patients: A meta-analysis. International Wound Journal. 2024;21(3):1-8. doi: 10.1111/iwj.14676.
58. Moore Z, Avsar P, O'Connor T, Budri A, Bader DL, Worsley P, et al. A systematic review of movement monitoring devices to aid the prediction of pressure ulcers in at-risk adults. International Wound Journal. 2023;20(2):579-608. doi: 10.1111/iwj.13902.
59. Mordiffi SZ, Kent B, Phillips N, Chi Tho P. Use of mobility subscale for risk assessment of pressure ulcer incidence and preventive interventions: A systematic review. JBI Library of Systematic Reviewis. 2011;9(56):2417-81.
60. Nixon J, Nelson EA, Rutherford C, Coleman S, Muir D, Keen J, et al. Pressure UlceR Programme Of reSEarch (PURPOSE): using mixed methods (systematic reviews, prospective cohort, case study, consensus and psychometrics) to identify patient and organisational risk, develop a risk assessment tool and patient-reported outcome Quality of Life and Health Utility measures. Programme Grants for applied research. 2015;3(6). doi: 10.3310/pgfar03060.
61. Richardson A, Barrow I. Part 1: Pressure ulcer assessment - the development of Critical Care Pressure Ulcer Assessment Tool made Easy (CALCULATE). Nursing in critical care. 2015;20:308-14. doi: 10.1111/nicc.12173.
62. Teixeira RGF, Guedes lB, Lima NS, des de Oliveira DF, Marinho CS, David RAR. Risk factors for pressure injury in critically ill polytraumatized patients: A systematic review. Saude Coletiva. 2022;12(82):11774-81. doi: 10.36489/saudecoletiua.2022v12i82p11766-11781.
63. Ting JJ, Garnett A. E-Health Decision Support Technologies in the Prevention and Management of Pressure Ulcers: A Systematic Review. Computers, informatics, nursing: CIN. 2021;39:955-73. doi: 10.1097/CIN.0000000000000780.
64. Toffaha KM, Simsekler MCE, Omar MA. Leveraging artificial intelligence and decision support systems in hospital-acquired pressure injuries prediction: A comprehensive review. Artificial intelligence in medicine. 2023;141:102560. doi: 10.1016/j.artmed.2023.102560.
65. Fuentelsaz Gallego C, Hernandez Faba E, Bermejo Caja C, Hermoso Villar P, Gallart Vive E, Blasco Garcia C, et al. Review of literature on pressure ulcers in people aged 65 or over. [Spanish]. Gerokomos. 2005;16:166-73.
66. Garcia-Fernandez FP, Pancorbo-Hidalgo PL, Soldevilla Agreda JJ, Rodriguez Torres Ma DC. Risk assessment scales for pressure ulcer in intensive care units: A systematic review with metaanalysis. [Spanish]. Gerokomos. 2013;24:82-9. doi: 10.4321/s1134-928x2013000200007.
67. Kottner J, Tannen A, Dassen T. Interrater reliability of the Braden scale. Pflege. 2008;21(2):85-94. doi: 10.1024/1012-5302.21.2.85.
68. Nunes de Sousa JE, Alves Alencar Pereira LM, Silva Sousa BC, da Silva Paes Landim JK, dos Santos Silva A, Oliveira Bastos A, et al. ESCALAS UTILIZADAS PARA MENSURAR O RISCO DE LESÃO POR PRESSÃO EM PACIENTES HOSPITALIZADOS: UMA REVISÃO. Enfermagem Atual in Derme. 2023;97(2):1-15. doi: 10.31011/reaid-2023-v.97-n.2-art.1573.
69. Pancorbo-Hidalgo PL, García-Fernández FP, Soldevilla-Agreda JJ, Martínez-Cuervo F. Pressure ulcers risk assessment: clinical practice in Spain and a meta-analysis of scales effectiveness. Gerokomos. 2008;19(2):40-54.

70. Park SH, Park YS. Predictive validity of the Braden Scale for pressure ulcer risk: a meta-analysis. [Korean]. Journal of Korean Academy of Nursing. 2014;44:595-607. doi: 10.4040/jkan.2014.44.6.595.
71. 杨昭霞, 吴春梅, 戴靖华, 张端香, 张平, 李亚楠, et al. [Predictive validity of the Munro Scale for pressure injuries in surgical patients: A meta-analysis] Munro量表对手术患者压力性损伤预测效果的Meta分析. Nursing of Integrated Traditional Chinese & Western Medicine. 2019;5(11):14-8. doi: 10.11997/nitcwm.201901104.
72. Barghouthi EaD, Owda AY, Asia M, Owda M. Systematic Review for Risks of Pressure Injury and Prediction Models Using Machine Learning Algorithms. Diagnostics (Basel, Switzerland). 2023;13(17). doi: 10.3390/diagnostics13172739.
73. Dweekat OY, Lam SS, McGrath L. Machine Learning Techniques, Applications, and Potential Future Opportunities in Pressure Injuries (Bedsores) Management: A Systematic Review. Int J Environ Res Public Health. 2023;20(1). doi: 10.3390/ijerph20010796.
74. Jiang M, Ma Y, Guo S, Jin L, Lv L, Han L, et al. Using Machine Learning Technologies in Pressure Injury Management: Systematic Review. JMIR Medical Informatics. 2021;9(3):e25704. doi: 10.2196/25704.
75. Ribeiro F, Fidalgo F, Silva A, Metrôlho J, Santos O, Dionisio R. Literature review of machine-learning algorithms for pressure ulcer prevention: Challenges and opportunities. Informatics. 2021;8(4):76. doi: 10.3390/informatics8040076.
76. Shi C, Dumville JC, Cullum N. Evaluating the development and validation of empirically-derived prognostic models for pressure ulcer risk assessment: A systematic review. Int J Nurs Stud. 2019;89:88-103. doi: 10.1016/j.ijnurstu.2018.08.005.
77. Zhou Y, Yang X, Ma S, Yuan Y, Yan M. A systematic review of predictive models for hospital-acquired pressure injury using machine learning. Nursing open. 2022;30. doi: 10.1002/nop2.1429.
78. Nayar SK, Li D, Ijaiya B, Lloyd D, Bharathan R. Waterlow score for risk assessment in surgical patients: a systematic review. Ann R Coll Surg Engl. 2021;103(5):312-7. Epub 2021/04/14. doi: 10.1308/rcsann.2020.7136.
79. Zahia S, Garcia Zapirain MB, Sevillano X, González A, Kim PJ, Elmaghraby A. Pressure injury image analysis with machine learning techniques: A systematic review on previous and possible future methods. Artif Intell Med. 2020;102:101742. doi: 10.1016/j.artmed.2019.101742.
80. Moore ZE, Cowman S. Risk assessment tools for the prevention of pressure ulcers. Cochrane Database Syst Rev. 2008;(3):Cd006471. Epub 2008/07/16. doi: 10.1002/14651858.CD006471.pub2.
81. Moore ZEH, Cowman S. Risk assessment tools for the prevention of pressure ulcers. Cochrane Database of Systematic Reviews. 2014;(2). doi: 10.1002/14651858.CD006471.pub3. PubMed PMID: CD006471.
82. Liao Y, Gao G, Mo L. Predictive accuracy of the Braden Q Scale in risk assessment for paediatric pressure ulcer: A meta-analysis. Int J Nurs Sci. 2018;5(4):419-26. Epub 2018/10/10. doi: 10.1016/j.ijnss.2018.08.003.
83. Ribeiro AP, Cruz I. How effective is the development of skin care in critically ill patients using the Braden Scale scores aiming to prevent the incidence of pressure ulcers? Sistematic Literature Review. Journal of Specialized Nursing Care. 2013;6(1).
84. Baris N, Karabacak BG, Alpar SE. The Use of the Braden Scale in Assessing Pressure Ulcers in Turkey: A Systematic Review. Adv Skin Wound Care. 2015;28:349-57. doi: 10.1097/01.ASW.0000465299.99194.e6.
85. Chen X, Diao D, Ye L. Predictive validity of the Jackson–Cubbin scale for pressure ulcers in intensive care unit patients: A meta-analysis. Nurs Crit Care. 2023;28(3):370-8. doi: 10.1111/nicc.12818.
86. Chen HL, Shen WQ, Liu P. A Meta-analysis to Evaluate the Predictive Validity of the Braden Scale for Pressure Ulcer Risk Assessment in Long-term Care. Ostomy Wound Manage. 2016;62(9):20-8.

87. Chou R, Dana T, Bougatsos C, Blazina I, Starmer AJ, Reitel K, et al. Pressure ulcer risk assessment and prevention: a systematic comparative effectiveness review. Ann Intern Med. 2013;159(1):28-38.
88. García-Fernández FP, Pancorbo-Hidalgo PL, Agreda JJS. Predictive Capacity of Risk Assessment Scales and Clinical Judgment for Pressure Ulcers: A Meta-analysis. J Wound Ostomy Continence Nurs. 2014;41(1):24-34. doi: 10.1097/01.WON.0000438014.90734.a2.
89. Gaspar S, Peralta M, Marques A, Budri A, Gaspar de Matos M. Effectiveness on hospital-acquired pressure ulcers prevention: a systematic review. Int Wound J. 2019;16(5):1087-102. doi: 10.1111/iwj.13147.
90. He W, Liu P, Chen HL. The Braden Scale cannot be used alone for assessing pressure ulcer risk in surgical patients: a meta-analysis. Ostomy Wound Manage. 2012;58:34-40.
91. Health Quality Ontario, Medical Advisory Secretariat. Pressure ulcer prevention: an evidence-based analysis. Ont Health Technol Assess Ser. 2009;9(2):1-104.
92. Huang C, Ma Y, Wang C, Jiang M, Yuet Foon L, Lv L, et al. Predictive validity of the braden scale for pressure injury risk assessment in adults: A systematic review and meta-analysis. Nurs Open. 2021;8:2194-207. doi: 10.1002/nop2.792.
93. Kottner J, Dassen T, Tannen A. Inter- and intrarater reliability of the Waterlow pressure sore risk scale: A systematic review. Int J Nurs Stud. 2009;46:369-79. doi: 10.1016/j.ijnurstu.2008.09.010.
94. Lovegrove J, Ven S, Miles SJ, Fulbrook P. Comparison of pressure injury risk assessment outcomes using a structured assessment tool versus clinical judgement: A systematic review. J Clin Nurs. 2021. doi: 10.1111/jocn.16154.
95. Lovegrove J, Miles S, Fulbrook P. The relationship between pressure ulcer risk assessment and preventative interventions: a systematic review. J Wound Care. 2018;27(12):862-75.
96. Mehicic A, Burston A, Fulbrook P. Psychometric properties of the Braden scale to assess pressure injury risk in intensive care: A systematic review. Intensive Crit Care Nurs. 2024;83:103686. doi: 10.1016/j.iccn.2024.103686.
97. Moore ZEH, Patton D. Risk assessment tools for the prevention of pressure ulcers. Cochrane Database Syst Rev. 2019. doi: 10.1002/14651858.CD006471.pub4.
98. Pancorbo-Hidalgo PL, Garcia-Fernandez FP, Lopez-Medina IM, Alvarez-Nieto C. Risk assessment scales for pressure ulcer prevention: a systematic review. J Adv Nurs. 2006;54(1):94-110. doi: 10.1111/j.1365-2648.2006.03794.x.
99. Park SH, Lee HS. Assessing Predictive Validity of Pressure Ulcer Risk Scales- A Systematic Review and Meta-Analysis. Iran J Public Health. 2016;45(2):122-33.
100. Park SH, Lee YS, Kwon YM. Predictive Validity of Pressure Ulcer Risk Assessment Tools for Elderly: A Meta-Analysis. West J Nurs Res. 2016;38:459-83. doi: 10.1177/0193945915602259.
101. Park SH, Choi YK, Kang CB. Predictive validity of the Braden Scale for pressure ulcer risk in hospitalized patients. J Tissue Viability. 2015;24:102-13. doi: 10.1016/j.jtv.2015.05.001.
102. Pei J, Guo X, Tao H, Wei Y, Zhang H, Ma Y, et al. Machine learning-based prediction models for pressure injury: A systematic review and meta-analysis. Int Wound J. 2023. doi: 10.1111/iwj.14280.
103. Qu C, Luo W, Zeng Z, Lin X, Gong X, Wang X, et al. The predictive effect of different machine learning algorithms for pressure injuries in hospitalized patients: A network meta-analyses. Heliyon. 2022;8(11):e11361. doi: 10.1016/j.heliyon.2022.e11361.
104. Tayyib NAH, Coyer F, Lewis P. Pressure ulcers in the adult intensive care unit: a literature review of patient risk factors and risk assessment scales. J Nurs Educ Pract. 2013;3(11):28-42.
105. Wang N, Lv L, Yan F, Ma Y, Miao L, Foon Chung LY, et al. Biomarkers for the early detection of pressure injury: A systematic review and meta-analysis. J Tissue Viability. 2022;31:259-67. doi: 10.1016/j.jtv.2022.02.005.
106. Wei M, Wu L, Chen Y, Fu Q, Chen W, Yang D. Predictive Validity of the Braden Scale for Pressure Ulcer Risk in Critical Care: A Meta-Analysis. Nurs Crit Care. 2020;25:165-70. doi: 10.1111/nicc.12500.

107. Wilchesky M, Lungu O. Predictive and concurrent validity of the Braden scale in long-term care: A meta-analysis. Wound Repair Regen. 2015;23:44-56. doi: 10.1111/wrr.12261.
108. Zhang Y, Zhuang Y, Shen J, Chen X, Wen Q, Jiang Q, et al. Value of pressure injury assessment scales for patients in the intensive care unit: Systematic review and diagnostic test accuracy meta-analysis. Intensive Crit Care Nurs. 2021;64:103009. doi: 10.1016/j.iccn.2020.103009.
109. Zimmermann GS, Cremasco MF, Zanei SSV, Takahashi SM, Cohrs CR, Whitaker IY. Pressure injury risk prediction in critical care patients: an integrative review. Texto & Contexto - Enfermagem. 2018;27(3). doi: 10.1590/0104-07072018003250017.
110. Pancorbo-Hidalgo PL, Garcia-Fern, ez FP, Lopez-Medina IM, Alvarez-Nieto C. Risk assessment scales for pressure ulcer prevention: a systematic review. Journal of advanced nursing. 2006;54(1):94-110. doi: 10.1111/j.1365-2648.2006.03794.x. PubMed PMID: rayyan-442041230.
111. Bergstrom N, Braden BJ, Laguzza A, Holman V. The Braden Scale for Predicting Pressure Sore Risk. Nurs Res. 1987;36(4):205-10.
112. Braden B, Bergstrom N. A Conceptual Schema for the Study of the Etiology of Pressure Sores. Rehabil Nurs. 1987;12(1):8-16. doi: 10.1002/j.2048-7940.1987.tb00541.x.
113. Song M, Choi KS. Factors predicting development of decubitus ulcers among patients admitted for neurological problems. Kanho Hakhoe Chi (The Journal of Nurses Academic Society). 1991;21(1):16-26.
114. Pang SM, Wong TK. Predicting pressure sore risk with the Norton, Braden, and Waterlow scales in a Hong Kong rehabilitation hospital. Nurs Res. 1998;47(3):147-53.
115. Kwong E, Pang S, Wong T, Ho J, Shao-ling X, Li-jun T. Predicting pressure ulcer risk with the modified Braden, Braden, and Norton scales in acute care hospitals in Mainland China. Appl Nurs Res. 2005;18(2):122-8. doi: 10.1016/j.apnr.2005.01.001.
116. Cubbin B, Jackson C. Trial of a pressure area risk calculator for intensive therapy patients. Intensive Care Nurs. 1991;7(1):40-4.
117. Jackson C. The revised Jackson/Cubbin Pressure Area Risk Calculator. Intensive Crit Care Nurs. 1999;15(3):169-75. doi: 10.1016/s0964-3397(99)80048-2.
118. Fuentelsaz C. Validation of the EMINA scale: tool for the evaluation of risk of developing pressure ulcers in hospitalized patients. Enferm Clin. 2001;11(3):97-103.
119. González-Ruiz J, Carrero AG, Blázquez MH, Vera RDV, Ortiz BG, Pulido M, et al. Factores de riesgo de las úlceras por presión en pacientes críticos. Enferm Clin. 2001;11(5):184-90.
120. Norton D. Geriatric nursing problems. Int Nurs Rev. 1962;9:39-41.
121. Ek AC. Prediction of pressure sore development. Scand J Caring Sci. 1987;1(2):77-84. doi: 10.1111/j.1471-6712.1987.tb00603.x.
122. Bienstein C. Risikopatienten erkennen mit der erweiterten Nortonskala [Risk patients detected with the extended Norton scale]. Pflege Aktuell (Verlag Krankenpflege). 1991;2(Dekubitus - Prophylaxe und Therapie).
123. Lowthian P. The practical assessment of pressure sore risk. Care : science and practice. 1987;5(4):3-7.
124. Waterlow J. Pressure sores: a risk assessment card. Nurs Times. 1985;81:49-55.
125. Andersen KE, Jensen O, Kvorning SA, Bach E. Prevention of pressure sores by identifying patients at risk. Br Med J (Clin Res Ed). 1982;284(6326):1370-1. doi: 10.1136/bmj.284.6326.1370.
126. Arnell I. Treating decubitus ulcers: two methods that work. Nursing. 1983;13(6):50-5. doi: 10.1097/00152193-198306000-00022.
127. Halfens R, Van Achterberg T, Bal R. Validity and reliability of the Braden scale and the influence of other risk factors: a multi-centre prospective study. Int J Nurs Stud. 2000;37(4):313-9.
128. Cobos Vargas A, Garofano Jerez J, Guardia Mesa M. Design and validation of a new rating scale to estimate the risk of pressure ulcer in patients attended in critical care units. Connect: The World of Critical Care Nursing. 2011;8(2):41.

129. Compton F, Hoffmann F, Hortig T, Strauss M, Frey J, Zidek W, et al. Pressure ulcer predictors in ICU patients: nursing skin assessment versus objective parameters. J Wound Care. 2008;17(10):417-20, 22-4. doi: 10.12968/jowc.2008.17.10.31304.
130. Prichard V. Calculating the risk. Nursing times. 1986;2:59-61.
131. Jiricka MK, Ryan P, Carvalho MA, Bukvich J. Pressure ulcer risk factors in an ICU population. Am J Crit Care. 1995;4(5):361-7.
132. Centraal Begeleidingsorgaan voor de Intercollegiale Toetsing (CBO). Development and validation of a pressure ulcer risk assessment tool for acute hospital patients. Utrecht: CBO, 1992.
133. Perneger TV, Raë AC, Gaspoz JM, Borst F, Vitek O, Héliot C. Screening for pressure ulcer risk in an acute care hospital: development of a brief bedside scale. J Clin Epidemiol. 2002;55(5):498-504. doi: 10.1016/s0895-4356(01)00514-5.
134. Gosnell DJ. An assessment tool to identify pressure sores. Nursing research. 1973;22(1):55-8.
135. Hatanaka N, Yamamoto Y, Ichihara K, Mastuo S, Nakamura Y, Watanabe M, et al. A new predictive indicator for development of pressure ulcers in bedridden patients based on common laboratory tests results. Journal of Clinical Pathology. 2008;61(4):514-8. doi: 10.1136/jcp.2007.050195.
136. Henoch I, Gustafsson M. Pressure ulcers in palliative care: development of a hospice pressure ulcer risk assessment scale. Int J Palliat Nurs. 2003;9(11):474-84. doi: 10.12968/ijpn.2003.9.11.11872.
137. Towey AP, Erland SM. Validity and reliability of an assessment tool for pressure ulcer risk. Decubitus. 1988;1(2):40-8. PubMed PMID: 3254237.
138. Bale S, Finlay I, Harding KG. Pressure sore prevention in a hospice. J Wound Care. 1995;4(10):465-8. doi: 10.12968/jowc.1995.4.10.465.
139. Stotts NA, Paul SM. Pressure ulcer development in surgical patients. Advances in Skin & Wound Care. 1988;1(3):24.
140. Ek A, Nordström G, Berglund B. Quality indicators for patients with or with risk of developing pressure ulcers. Quality indicators in nursing Stockholm: Spri Publications. 1997:21-38.
141. García Fernández FP, Bermejo Cobo J, Pérez Catalán MJ, Ramírez Pérez C, Fernández Romo MA, Cano Luque MC, et al. [Validation of two scales for evaluation of the risk of decubitus ulcer: Gosnell and Nova-4]. Rev Enferm. 1999;22(10):suppl 685-7. PubMed PMID: 10745869.
142. Lindgren M, Unosson M, Krantz AM, Ek AC. A risk assessment scale for the prediction of pressure sore development: reliability and validity. J Adv Nurs. 2002;38(2):190-9.
143. Suriadi Sanada H, Sugama J, Thigpen B, Subuh M. Development of a new risk assessment scale for predicting pressure ulcers in an intensive care unit. Nursing in critical care. 2008;13(1):34-43.
144. Lowery MT. A pressure sore risk calculator for intensive care patients: 'the Sunderland experience'. Intensive Crit Care Nurs. 1995;11(6):344-53. doi: 10.1016/s0964-3397(95)80452-8.
145. Page KN, Barker AL, Kamar J. Development and validation of a pressure ulcer risk assessment tool for acute hospital patients. Wound Repair and Regeneration. 2011;19(1):31-7. doi: 10.1111/j.1524-475X.2010.00647.x.
146. Watkinson C. Developing a pressure sore risk assessment scale. Prof Nurse. 1997;12(5):341-6, 8. PubMed PMID: 9128687.
147. Kaewprag P, Newton C, Vermillion B, Hyun S, Huang K, Machiraju R. Predictive models for pressure ulcers from intensive care unit electronic health records using Bayesian networks. Bmc Medical Informatics and Decision Making. 2017;17. doi: 10.1186/s12911-017-0471-z.
148. Ladios-Martin M, Fernández-de-Maya J, Ballesta-López F-J, Belso-Garzas A, Mas-Asencio M, Cabañero-Martínez MJ. Predictive modeling of pressure injury risk in patients admitted to an intensive care unit. American Journal of Critical Care. 2020;29(4):e70-e80.
149. Kim TY, Lang N, editors. Predictive modeling for the prevention of hospital-acquired pressure ulcers. AMIA Annual Symposium Proceedings; 2006: AMIA Symposium.

150. Kottner J, Dassen T. Pressure ulcer risk assessment in critical care: Interrater reliability and validity studies of the Braden and Waterlow scales and subjective ratings in two intensive care units. International Journal of Nursing Studies. 2010;47(6):671-7. doi: 10.1016/j.ijnurstu.2009.11.005.
151. Webster J, Coleman K, Mudge A, Marquart L, Gardner G, Stankiewicz M, et al. Pressure ulcers: effectiveness of risk-assessment tools. A randomised controlled trial (the ULCER trial). BMJ Qual Saf. 2011;20(4):297. doi: 10.1136/bmjqs.2010.043109.
152. Moore Z, Johansen E, van Etten M, Strapp H, Solbakken T, Eskerud Smith B, et al. Pressure ulcer prevalence and prevention practices: a cross-sectional comparative survey in Norway and Ireland. J Wound Care. 2015;24(8):333-9. doi: 10.12968/jowc.2015.24.8.333.
153. Voz A, Williams C, Wilson M. Who Is Turning the Patients? A Survey Study. Journal of Wound Ostomy & Continence Nursing. 2011;38(4).
154. Lima-Serrano M, González-Méndez MI, Martín-Castaño C, Alonso-Araujo I, Lima-Rodríguez JS. Predictive validity and reliability of the Braden scale for risk assessment of pressure ulcers in an intensive care unit. Medicina Intensiva (English Edition). 2018;42(2):82-91. doi: 10.1016/j.medine.2018.01.007.
155. Adibelli S, Korkmaz F. Pressure injury risk assessment in intensive care units: Comparison of the reliability and predictive validity of the Braden and Jackson/Cubbin scales. Journal of Clinical Nursing. 2019;28(23-24):4595-605. doi: 10.1111/jocn.15054.
156. Simão CMF, Caliri MHL, Santos CBd. Concordância entre enfermeiros quanto ao risco dos pacientes para úlcera por pressão. Acta Paulista de Enfermagem. 2013;26.
157. Fulbrook P, Anderson A. Pressure injury risk assessment in intensive care: comparison of inter-rater reliability of the COMHON (Conscious level, Mobility, Haemodynamics, Oxygenation, Nutrition) Index with three scales. Journal of Advanced Nursing. 2016;72(3):680-92. doi: 10.1111/jan.12825.
158. Veiga TA-O, Rêgo AA-OX, Montenegro WA-O, Ferreira PA-O, Rocha DA-OX, Felipe IA-O, et al. Braden scale has low reliability in different patients under care in intensive care unit. 2022;(1806-9282 (Electronic)).
159. Delawder JM, Leontie SL, Maduro RS, Morgan MK, Zimbro KS. Predictive Validity of the Cubbin-Jackson and Braden Skin Risk Tools in Critical Care Patients: A Multisite Project. American Journal of Critical Care. 2021;30(2):140-4. doi: 10.4037/ajcc2021669.
160. Saleh M, Anthony D, Parboteeah S. The impact of pressure ulcer risk assessment on patient outcomes among hospitalised patients. J Clin Nurs. 2009;18(13):1923-9. doi: 10.1111/j.1365-2702.2008.02717.x.
161. Gunningberg L, Lindholm C, Carlsson M, Sjödén PO. Implementation of risk assessment and classification of pressure ulcers as quality indicators for patients with hip fractures. J Clin Nurs. 1999;8(4):396-406. doi: 10.1046/j.1365-2702.1999.00287.x.
162. Hodge J, Mounter J, Gardner G, Rowley G. Clinical trial of the Norton Scale in acute care settings. Aust J Adv Nurs. 1990;8(1):39-46.
163. Bakanoğlu E. Evaluation Relationship Between Risk Factors and Pressure Ulcers of the Patients Whose Risk to Have Pressure Ulcer Has Been Determined With the Braden Scale for Predicting Pressure Ulcer Risk. Istanbul, Turkey: Istanbul University 2010.
164. Oğuz S, Olgun N. Predicting the pressure sore risk with Braden Scale and determining the effectiveness of predetermined nursing preventing of pressure sore. Hemşirelik Forumu Derg. 1998;3(1):131-5.
165. Dealey C. Risk assessment of pressure sores: a comparative study of Norton and Waterlow scores. Nurs Stand Spec Suppl. 1989;(3):11-2. doi: 10.7748/ns.3.27.11.s69.
166. Edwards M. The levels of reliability and validity of the Waterlow pressure sore risk calculator. Journal of wound care. 1995;4(8):373-8.
167. Watkinson C. Inter-rater reliability of risk-assessment scales. Professional nurse (London, England). 1996;11(11):751-2, 5.

168. Cook M, Hale C, Watson B. Interrater reliability and the assessment of pressure-sore risk using an adapted Waterlow Scale. Clinical Effectiveness in Nursing. 1999;3(2):66-74.
169. Hale C, Cook M, Watson B. Assessing the interrater reliability of the Waterlow pressure sore risk assessment scale. Unpublished report. 1999.
170. Kelly J. Inter-rater reliability and Waterlow's pressure ulcer risk assessment tool. Nurs Stand. 2005;19(32):86-7, 90-2. doi: 10.7748/ns2005.04.19.32.86.c3851.
171. Jalali R, Rezaie M. Predicting pressure ulcer risk: comparing the predictive validity of 4 scales. Adv Skin Wound Care. 2005;18(2):92-7. doi: 10.1097/00129334-200503000-00013.
172. Lewicki LJ, Mion LC, Secic M. Sensitivity and specificity of the Braden Scale in the cardiac surgical population. J Wound Ostomy Continence Nurs. 2000;27(1):36-41. doi: 10.1016/s1071-5754(00)90039-1.
