# Supplementary material for: Accuracy and clinical effectiveness of risk prediction tools for pressure injury occurrence: An umbrella review
Source: PLoS Med. 2025 Feb 6;22(2):e1004518. doi: 10.1371/journal.pmed.1004518 (PMC11844857; doi:10.1371/journal.pmed.1004518)
Supplement: Attachment — Submitted filename: Cuddigan PLOS review.docx [file pmed.1004518.s002.docx]

Thank you for allowing me to review your excellent manuscript. As a member of the International Pressure Ulcer/Injury Guideline Governance Group (GGG), I have followed the literature related to pressure ulcer risk assessment closely and appreciate the amount of work that went into this umbrella review.

**Methodology:**

- I have reviewed Chapter 5 of the **Cochrane Handbook on Umbrella Reviews** and believe you have followed their guidance carefully.
- In my initial review of the manuscript, I was concerned that there was **no exclusionary cut-off based on AMSTAR-2 assessments**. Some investigators exclude systematic reviews of poor quality. It appears that exclusions are primarily based on appropriateness of content and not quality. The Cochrane guidance seems to provide some latitude for including a broader range of systematic reviews in an umbrella review.
  - However, the result in this case (based on the quality of the systematic reviews available to you)… is that “*The quality of included reviews was generally poor***…”** (Line 219). You can’t change the quality of materials you had to work with; however, the **poor quality of source materials should “temper your conclusions”.**
  - Example: Your conclusion in the abstract states that *“There is* ***no*** *reliable evidence to suggest that using existing risk prediction tools effectively reduces the incidence of PIs.”* Such a strong, absolute statement should not be made based on poor quality systematic reviews. It is also not entirely true. Several studies reported lower PI incidence when risk assessment tools were used to guide prevention (Lovegrove, 2021; Chou, 2013; Health Quality Ontario, 2009). There is also evidence of improved intermediate outcomes (e.g., more preventive interventions) when risk assessment tools are used. See pages 19-20 for mixed results. **Can you modify “no” to “limited”? in this example and review the document for other absolute statements that may not be justified by the quality of systematic reviews or the findings of high-quality studies.**
- **Diagnostic vs. Prognostic Tests.** There are subtle, but important differences between diagnostic and prognostic tests.
  - With diagnostic tests, the disease exists. You are determining the accuracy of a diagnostic test to detect an existing disease.
  - With prognostic tests, you are predicting the likelihood of future disease based on modifiable and non-modifiable risk factors. Prediction is not destiny. The true value of a prognostic test (e.g., risk assessment tool) is to identify and mitigate the modifiable risk factors thus preventing the disease. Under that clinical use scenario, lower sensitivity would be a desired outcome (i.e., If fewer subjects with a positive predictive test actually develop the disease…it may well indicate that your preventive measures were successful in preventing disease)
  - The umbrella review methodology draws on several references to diagnostic testing accuracy (DTA). **Please review how these references are used and applied.** **DTA principles may have some similarities with prognostic testing accuracy, but the intended clinical use and context needs to be explained and interpretation of results modified accordingly.**
  - in diagnosing an existing breast cancer, I would prefer both Sensitivity and Specificity to as close to 100% as possible.
  - **What is a desired, preferred or good Se and Sp for prognostic testing? Have we taken into account the use of the prognostic test in guiding prevention (seems like an intervening variable)? Have we truly studied the moderating effects of preventive interventions on the final (not predicted) outcome?**
  - References to DTA in the manuscript are listed below. **I would just ask that you discuss the limitations of DTA principles when applied to prognostic tests.**
    - *Preferred Reporting Items for Systematic Reviews and Meta-Analyses of Diagnostic Test Accuracy Studies’ (PRISMA-DTA)*
    - *Bossuyt PM, Reitsma JB, Bruns DE, et al. The STARD statement for reporting studies of diagnostic 725 accuracy: explanation and elaboration. Ann Intern Med 2003;138(1):W1-12. doi: 726 10.7326/0003-4819-138-1-200301070-00012-w1*
    - *All of your references for diagnostic testing accuracy seem to apply to diagnostic testing only and not diagnostic and prognostic testing.*
- **I would just ask that you review your manuscript to see if you have fairly applied principles of diagnostic testing accuracy to prognostic testing accuracy. Have you stated the limitations?** Your reader is left with the impression that good, reliable, valid, accurate prognostic test should have high Se, Sp, AUROC values. I would argue that is not only not feasible but may not be the best measure of clinical effectiveness.
- **I don’t think we have answered the basic question: Why do we do pressure injury risk assessments? Is our primary goal to PREDICT? OR Is our primary goal to use the assessment findings (especially modifiable risks) to PREVENT pressure injuries? Our criterion for good prognostic test is very different depending on which question you ask. I realize that the science has focused on predictive accuracy. I just wonder if our time is best spent continuing down that pathway. We will never find the perfect tool. There is a lot we can do in studying how risk assessment tools can be utilized in guiding prevention and producing better outcomes.**
- **I had some concerns about the general characterization of pressure injury risk assessment tools starting at line 76. It ignores the body of work that uses epidemiological evidence to develop tools that identify modifiable risk factors that nurses can address. I am not sure you can say “***many are limited by reliance on subjective clinical judgment” when interrater reliability is high in trained staff.* It is true that the tools do not contain all risk factors….but they were never designed to. The impetus for risk assessment tools was to translate known science into a quick and reliable assessment tool that guides risk-based prevention of the modifiable risk factors amenable to nursing interventions.
- I have followed the **clinical effectiveness studies** carefully over the years. The underlying premise of many of these studies is flawed. No clinician would claim that the simple act of assessing cardiovascular risk factors would prevent myocardial infarctions. Or that taking your blood pressure everyday will eliminate your risk of stroke. YOU NEED TO DO SOMETHING WITH THE ASSESSMENT INFORMATION TO MITIGATE THE RISKS. Too few of the clinical effectiveness studies describe how the risk assessments are used to guide prevention.
- The Agency for Healthcare Policy and Research developed a pressure injury prevention guide many years ago that recommends preventive interventions be designed to address both the type of level of risk on Braden Scale subscore (and that additional risk factors be considered). Preventive interventions are different for immobility vs. moisture vs. nutritional deficits. The intensity of the intervention is aligned with the level of risk, with more intense and expensive interventions reserved for higher risk subscale scores. A matrix of interventions has been developed based on type and level of risk and incorporated into computerized care planning systems. In many settings this has been more effective than determining a “risk score” and applying a bundle of interventions. Regrettably, this common practice has been documented in the quality improvement and implementation research, but not in randomized clinical trials. This risk-based prevention strategy may not be the best fit in every setting. However, it does illustrate that risk assessment tools can be used to guide risk-based prevention plans. **To truly understand the clinical effectiveness (or not) of risk assessment tools, you need to describe how the assessment findings were used to guide risk-based prevention.**

**Conclusion:** I have great respect for the work you have completed with this umbrella review. Your work summarizes what is known (from relatively poor-quality systematic reviews) but I am not sure it provides new insights or direction for future research. You suggest that further research is needed on clinical effectiveness but only once promising prediction tools have been developed and validated. How do we accomplish this? What are the criteria for promising prediction tools and methods to determine clinical effectiveness? What is a good prediction tool/risk assessment tool? Are designs and methodologies from implementation science more appropriate to demonstrate clinical effectiveness than RCTs designed to demonstrate efficacy?

**Are we asking the right questions?**

- **Is our primary objective in using risk assessment tools to PREDICT or PREVENT pressure injuries?**
- **Can we truly measure “clinical effectiveness” if we don’t consider how the risk assessment tool is being used in the clinical setting?**

*My fear is that if we continue down the same path of investigation, we will find ourselves with the same inadequate and disappointing answers. I would be willing to write a short editorial commentary that acknowledges your work….. but also asks:* ***“Are we asking the right questions?”*** *Your manuscript is excellent and I am recommending publication with minor revisions. I think my questions and your response would generate excellent ideas for further direction in research and systematic reviews. I am willing to write a commentary if that is of interest to the editors and authors. Thank you for allowing me to review this manuscript.*

*Janet Cuddigan, PhD, RN, FAAN*

*Professor, University of Nebraska Medical Center*
