# Supplementary material for: Accuracy and clinical effectiveness of risk prediction tools for pressure injury occurrence: An umbrella review
Source: PLoS Med. 2025 Feb 6;22(2):e1004518. doi: 10.1371/journal.pmed.1004518 (PMC11844857; doi:10.1371/journal.pmed.1004518)
Supplement: Attachment — Submitted filename: 120924 - PMed Editor+reviewer - author response to comments.docx [file pmed.1004518.s003.docx]

PLOS Medicine editor & reviewer comments for: ***"Accuracy and clinical effectiveness of risk prediction tools for pressure injury occurrence: An umbrella review”***

| **Editor comments** | **Author replies** |
| --- | --- |
| Editor comment #1: We really enjoyed reading your paper and were pleased to see that it was also well received by the reviewers. We agree that it is not without limitations as currently written and require that you address each of the editorial and reviewer points detailed below in full.  Specifically, we agree with the statistical reviewer that a statistical analysis of the evidence would be beneficial. Ideally “researchers should use the study specific data extracted from each SRMA to repeat each meta-analysis separately rather than report the meta-analytical result as presented in the original SRMA” ( see, <https://bmjmedicine.bmj.com/content/1/1/e000071> for reference). | Thank you for your positive feedback. Please see below for our replies to each of the suggestions and comments made.  Regarding the statistical analysis request, we would first like to thank the editor for bringing the BMJ article to our attention, which provides useful guidance for conducting umbrella reviews. However, there are differing views about the value of re-analysis of data presented in systematic reviews when conducting umbrella reviews. As we stated in the methods, we followed Cochrane guidance for conducting overviews/umbrella reviews [**Pollock M, Fernandes RM BL, Pieper D, Hartling L,. Chapter V: Overviews of Reviews. In: Higgins JPT TJ, Chandler J, Cumpston M, Li T, Page MJ, Welch VA ed. Cochrane Handbook for Systematic Reviews of Interventions version 63 (updated February 2022). Available from www.training.cochrane.org/handbook: Cochrane 2022.**]. The Cochrane Handbook does not specify that meta-analysis of each review should be redone solely due to concerns about inappropriate meta-analysis methods, as is suggested in the BMJ article. According to the Cochrane Handbook ([**https://training.cochrane.org/handbook/current/chapter-v#section--4-12-2**](https://training.cochrane.org/handbook/current/chapter-v#section--4-12-2)), the four reasons to re-analyse outcome data in included systematic reviews are to: 1) answer a different clinical question, 2) address specific populations or subgroups, 3) use different summary measures across reviews, or 4) meta-analyse data that was not originally meta-analysed. None of these reasons apply to our umbrella review. Therefore, by not re-doing meta-analyses in the included reviews, we believe we have correctly followed this guidance.  Furthermore, review-level information is often insufficient to assess heterogeneity between primary studies, which is crucial to determine whether meta-analysis is feasible. Although it is possible to return to the primary publications to obtain the necessary data, as suggested by the BMJ article, this approach conflicts with Cochrane’s guidance for conducting umbrella reviews. The Cochrane Handbook explicitly advises against accessing primary publications, specifically stating: *"Note that authors wishing to re-analyse outcome data may only be able to do so if the clinical parameters and statistical aspects of the included systematic reviews are sufficiently reported.".*  There are also several other challenges with re-doing meta-analyses for each of the reviews in this specific instance. Firstly, many of the primary studies included in these systematic reviews have been assessed as being at high risk of bias by the systematic review authors. Therefore, we believe that re-doing meta-analyses using correct methodology on methodologically flawed studies will not add substantial value to our umbrella review. In fact, we believe results from re-analysis of data from the systematic reviews could in fact be misleading by giving too much weighting to summary estimates of sensitivity and specificity.  Given the nature of our umbrella review, assessing heterogeneity using review-level information is particularly difficult. To enable the estimation of summary sensitivities and specificities, the positivity threshold must be the same in all studies included in the meta-analysis. If estimates are pooled across multiple thresholds, the summary estimates do not have a clinical interpretation. The positivity thresholds are poorly reported in the included systematic reviews, which would mean we need to access primary studies. As previously described, this goes against the Cochrane guidance. In addition, the risk prediction tools are not diagnostic tests. Therefore, the time interval between applying the risk prediction tools and the reference standard should be the same in all studies included in a meta-analysis. As a result, we would need to know the time points at which sensitivity and specificity have been calculated. Again, this is poorly reported at review level.  Aside, even if there was sufficient information presented in the systematic reviews to allow for a full assessment of heterogeneity to determine whether meta-analysis is feasible, to enable us to re-analyse the data presented in the systematic reviews without going back to the primary studies, the reviews would need to present sufficient raw data, such as 2x2 tables, for each of the primary studies included in the review. Unfortunately, not all of the systematic reviews include this information.  Whilst we have carefully considered the option of conducting a meta-analysis, given the guidance in the Cochrane Handbook, and the other issues associated with conducting meta-analyses of data from systematic reviews in this specific instance, we have concluded that it is not appropriate for our review. We hope that the editor and reviewer #4 agree. |
| Editor comment #2: Many of the editorial requests detailed below pertain to specific formatting and content requirements. Some may have already been incorporated into the manuscript and some may not apply but please review the complete list of items and ensure that each item is included as necessary. | See below. |
| Editor comment #3: Reporting guidance Thank you for reporting your study in line with PRISMA-DTA guidance and for including the checklist. Please amend the checklist to refer to section and paragraph numbers as opposed to line numbers as these often change at publication.  Please ensure that your search is updated to the current time. PLOS Medicine requires that searches are updated to within roughly 6 months of a potential publication date. | Thank you for these comments. We agree and have implemented both points.  Firstly, we have referred to sections and paragraph numbers for the PRISMA-DTA checklist (see Appendix 1).  Secondly, we have updated our search (in June 2024). We screened another 1351 results (898 after deduplication), leaving us with three additional included systematic reviews (SRs) in the end. Incorporating these into our umbrella review, we now have 26 included SRs in total. |
| Editor comment #4: Statistical reporting Please quantify the main results with 95% CIs and p values. When reporting p values please report as <0.001 and where higher as p=0.002, for example. When reporting 95% CIs please separate upper and lower bounds with commas instead of hyphens as the latter can be confused with reporting of negative values. Please include the actual amounts and/or absolute risk(s) of relevant outcomes (including NNT or NNH where appropriate), not just relative risks or correlation coefficients. (example for absolute risks: PMID: 28399126). Please include any important dependent variables that are adjusted for in the analyses. | Thank you. We have gone through our umbrella review manuscript and appendices document to ensure our results, confidence intervals and p-values are reported appropriately. On some occasions, this is not possible, where there has been poor or inconsistent reporting in/between included reviews, but wherever possible, we have included such measures of uncertainty. |
| Editor comment #5: Abstract layout Please structure your abstract using the PLOS Medicine headings (Background, Methods and Findings, Conclusions). | N/A  This has already implemented. |
| Editor comment #6: Author summary At this stage, we ask that you include a short, non-technical Author Summary of your research to make findings accessible to a wide audience that includes both scientists and non-scientists. The authors summary should consist of 2-3 succinct bullet points under each of the following headings: • Why Was This Study Done? Authors should reflect on what was known about the topic before the research was published and why the research was needed. • What Did the Researchers Do and Find? Authors should briefly describe the study design that was used and the study’s major findings. Do include the headline numbers from the study, such as the sample size and key findings. • What Do These Findings Mean? Authors should reflect on the new knowledge generated by the research and the implications for practice, research, policy, or public health. Authors should also consider how the interpretation of the study’s findings may be affected by the study limitations. In the final bullet point of ‘What Do These Findings Mean?’, please describe the main limitations of the study in non-technical language. The Author Summary should immediately follow the Abstract in your revised manuscript. This text is subject to editorial change and should be distinct from the scientific abstract. Please see our author guidelines for more information: <https://journals.plos.org/plosmedicine/s/revising-your-manuscript#loc-author-summary> | Thank you; we have added this summary to the manuscript. |
| Editor comment #7: Introduction layout Please address past research and explain the need for and potential importance of your study. Indicate whether your study is novel and how you determined that. If there has been a systematic review of the evidence related to your study (or you have conducted one), please refer to and reference that review and indicate whether it supports the need for your study. | Thank you – these points have already been addressed in our introduction. |
| Editor comment #8: Discussion layout Please present and organize the Discussion as follows: a short, clear summary of the article's findings; what the study adds to existing research and where and why the results may differ from previous research; strengths and limitations of the study; implications and next steps for research, clinical practice, and/or public policy; one-paragraph conclusion. | Thank you - we have presented and organised our discussion as recommended. We have included two paragraphs in our conclusion, rather than one, due to the breadth of the topics and information included in our umbrella review. However, we can combine these into one paragraph, if needed. |
| Editor comment #9: Supporting information Please cite your Supporting Information as outlined here: <https://journals.plos.org/plosmedicine/s/supporting-information>  In the published article, supporting information files are accessed only through a hyperlink attached to the captions. For this reason, you must list captions at the end of your manuscript file. You may include a caption within the supporting information file itself, as long as that caption is also provided in the manuscript file. Do not submit a separate caption file. | Thank you for this guidance. We have double-checked the PLOS Medicine guidance regarding the supporting information. We have amended the references to the appendices, e.g. “Appendix 1 in S1 File”, as recommended. Additionally, on the final page of the manuscript, a caption for the hyperlink to S1 File has been added. |

| **Comment number** | **Reviewer comments** | **Author replies** |
| --- | --- | --- |
|  | **Reviewer #1:** |  |
| **1** | Good to go | N/A |
|  | **Reviewer #2:** |  |
| **2** | Thank you for the opportunity to review this manuscript. It highlights the ongoing problems/ lack of clarity with studies relating to predictive tools in PIs. | N/A |
| **3** | Line 111 - It is a bit unclear which author conducted the screening, i.e., which author plays the third reviewer role. | We have clarified the text as follows (Line 159):  “Screening of search results and full texts were conducted independently and in duplicate by any two from a group of four reviewers (BH, JD, YT, KS), with arbitration by a third reviewer where necessary (any one of the four reviewers not involved in the independent screening)”  Screening was conducted independently and in duplicate by two of the following reviewers: BH, JD, YT, KS. Screening was implemented until all articles had been given a decision by two reviewers. For any articles where there were disagreements, a third reviewer (one of BH, JD, YT, KS), who was not one of the two who had already screened the article, made the decision on whether to include or not. Articles were sorted in alphabetical order (by author) and reviewers started at various letters in that order, therefore different articles were screened by different pairs of reviewers. |
| **4** | Line 156 - It is stated a total of 110, but Fig.1 indicates 111? | Thank you for spotting this error.  With the update of the search the correct figure is now “118 records” (Line 212). |
| **5** | Line 189 - Just confirming if the mention of acute care here also includes hospital. If not, would the effectiveness review which stated 4/10, actually be 2? If yes, then the accuracy review would be 7/16, not 5/16. | Thank you for your comment. You are correct, that we did not include ‘hospital’ in the ‘acute care’ group, and the correct figure should have been 2/10 (20%) as was presented in Table 1.  With the new search update, the correct figure is now “3/11 (27%)” (Line 222). |
| **6** | Line 221 - Just checking the reported figure, i.e., should conflict of interest be 18/23 (not 17/23)? | The original figure of 17/23 was correct because there is an overlap of systematic reviews in the ‘accuracy’ subgroup and the ‘effectiveness’ subgroup. One of these reported conflicts of interest (the Chou 2013 review) and was included in this count of 17 reviews.  The updated figure is “20/26, 77%” (Line 280). |
| **7** | Figure 2 has been crafted very nicely, and is very easy to read. Well done. | Thank you for your positive feedback. |
|  | **Reviewer #3:** |  |
| **8** | First of all, I would like to express my appreciation to the authors for conducting this meaningful study. Pressure injuries are a critical aspect of clinical care, and there is a huge number of studies on this topic. Thus, synthesizing existing information is highly valuable. This study provides a wealth of information on pressure injuries and offers valuable insights and essential information to readers. While the topic is very interesting and relevant, there are some areas that need improvement. This paper provides a large amount of information, which seems to diminish the emphasis on the primary objective of the research. Given the nature of an umbrella review, it is appropriate to assess the quality of the papers reviewed. However, this aspect occupies a large amount of the content (e.g., Brief description of included study quality in Table 2). I suggest summarizing these evaluations or highlight the main objective of the study more effectively. | Thank you for your positive comments regarding the value of the work reported. We also agree that assessing the quality of evidence is both appropriate and crucial. Through assessing the included systematic reviews using AMSTAR-2 and presenting the quality assessments of primary studies conducted by the systematic review authors, we can then factor this into our consideration of their results and conclusions. However, as you have suggested, to ensure that this information is conveyed more clearly to the reader, we have summarised the quality assessment descriptions in Table 2. The results are now presented in a more succinct and concise manner. |
| **9** | 1. The terminology for pressure injury is used inconsistently throughout the paper, with terms like "pressure injury", "pressure ulcers,", and "PrI". Please standardize the terminology for clarity. | Thank you for spotting this. We have gone through and ensured “pressure injury” and subsequently the abbreviation, “PI”, have been used consistently throughout. |
| **10** | 2. Table 1 contains several ambiguous expressions that need clarification. - [Presents of pressure injury at baseline]: What is the definition of "baseline"? Does this refer to the presence of pressure injuries at the start of the study? - [Setting]:  Aren't "hospital" and "acute care (including surgical and ICU)" overlapping categories? - In the Eligibility section, please replace the term "any" with a more precise/clear expression. - [Source of data]: There seems to be an overlap between "Prospective only" and "prospective OR retrospective" as both include "prospective." Please verify whether it should be "prospective AND retrospective." | Thank you for your comment. We have tried to clarify the following points below and by revising Table 1 & adding footnotes. Additionally, further details regarding review characteristics can be found in Appendix 5 Table A2 in S1 File.   - [PIs at baseline]   This category is intended to indicate whether or not the reviews restricted study eligibility to those including only participants who did not have PI *at the start of the study.* Although there was considerable variation in the wording used, we considered 6 reviews to have met this criterion. Eligibility criteria included “patients without PI” (n=1), “no PI at baseline” (n=1), “no PI at beginning of study” (n=1), and three reviews referred to PI on hospital admission. A footnote has been added to Table 1 for some clarification.   - [Setting]   Thank you for your insight. It is possible that there is overlap between ‘hospital’ and ‘acute care’, however ‘hospital’ could also include non-acute patients, therefore we did not consider is sensible to lump these two groups together. We have now refined the categorisations used in order to be more explicit and hopefully avoid confusion. Some reviews just specify ‘hospital’ (they fall into the hospital group), some reviews specify ‘hospital or acute’ (they now fall into the ‘hospital or acute’ group), and other reviews specify acute settings (they fall into the acute group).  [Eligibility section]  Thank you, we have now replaced all instances of ‘Any’ in Table 1 with a more specific definition of what ‘Any’ was intended to refer to. For the most part, ‘any’ is used where the original reviews used this descriptor, although in some cases it was inferred by us as follows:   - Regarding age, both reviews state “any age” in their inclusion criteria. - Regarding setting, both reviews now categorised under ‘any healthcare setting’, specifically stated ‘any’ healthcare setting in their inclusion criteria. - Regarding risk assessment tools, the nine reviews in this group either used terms like “a risk assessment instrument” (any implied), or explicitly stated “any risk assessment scale”, for instance. - Regarding PI classification systems, the one review included here required that *“the standard for judging the occurrence of PI were described in the studies*” but did not restrict beyond that description. - [Source of data]   In fact, this should be "prospective OR retrospective”. This is summarising what the included systematic reviews have specified in their eligibility criteria. So, some reviews have specified prospective studies only, whereas other reviews have allowed inclusion of either “prospective or retrospective” studies (but then have not necessarily always ended up including both). We have not allowed any overlap between these categories. We hope this clarifies matters. |
| **11** | 3. According to the Appendix, only Google Scholar limited the search period to 2003-2013. I would recommend to mention this period in the methodology section of the manuscript. | Thank you. The Google Scholar search was limited to 2013-2023 (and updated to include 2024), due to pragmatic reasons as export functionality is poor. We have now stated the date limit in the manuscript, as suggested. Please see Line 158. |
| **12** | 4. On page 18, it is mentioned that there are additional reviews ("The 341 individually randomized trial was included in three additional reviews29 44 47 50, each of which 342 considered the trial to be 'good quality'29, 'valid'44, or 'high quality'50). However, four references (29 44 47 50) are provided. It seems that reference 47 might be inappropriately included in the manuscript, and it should be verified. | Thank you for pointing this out – good spot! We have verified the references for this statement, and it has now been amended (see Line 410). |
| **13** | 5. While I do not recommend adding more content, I suggest considering the inclusion of this information in the author's decision. It may be beneficial to provide definitions or information of the representative pressure ulcer assessment tools (Braden, Norton, and Waterlow). Offering readers an understanding of the purpose and differences of these tools would enhance the practical application of the study, enabling them to identify the most effective and valid tool for their needs. | Thank you for your suggestion. Although we agree that too much additional content would not be helpful, it is important to establish understanding and important context in the introduction. Therefore, we have added the following to our introduction (see from Line 119):  *“The Braden^6 7^, Norton^8^ and Waterlow^9^ scales are recommended by NICE guidelines^10^ in the UK and referenced in international guidelines for PI prevention.^11^ In some hospitals and long-term care settings in the US, healthcare professionals must conduct mandatory risk assessments for PI for all patients for the purposes of risk stratification and clinical triage. The Braden scale, developed in 1987 using a sample of 102 elderly hospital patients in the US includes sensory perception, moisture, activity, mobility, nutrition, friction and shear as predictors.^6 7^ The Norton scale, based on a sample of 250 elderly hospital patients in the UK (published in 1962), includes physical condition, mental status, activity, mobility and continence domains.^8^ The Waterlow scale was published in 1985 for use by Waterlow’s nursing students in the UK^12^, and assesses BMI, assessment of the skin, gender, age, malnutrition, incontinence, mobility, tissue malnutrition, neurological deficits, major surgery or trauma and medication.^9^”* |
| **14** | 6. The paper focuses on the technical aspects of the major findings, while lacking sufficient clinically based explanations. Would there be a reason for this approach? For instance, if the Braden scale does not reduce the incidence of falls, there is a need for clinical interpretation of this result or a discussion on what aspects need improvement. If providing such an analysis is challenging, it could be included as a limitation of the study. | Thank you for your valuable suggestion. As discussed in the paragraph from Line 408, the highest quality systematic review (Cochrane review by Moore et al.) included two RCTs, and both were rated as being at high RoB. One of these RCTs (Webster, 2011) found no evidence of a difference in patient management or PI severity between groups (Waterlow or Ramstadius tools vs. clinical judgement alone), and the other RCT (Saleh, 2009) also found no evidence of a difference in PI incidence between groups (Braden vs. no training).  The lack of evidence for a difference could be influenced by several factors including sample size and study quality. With the Webster study, no difference was found in PI management between the groups, therefore it would be surprising if a difference in PI incidence would be found. Regarding the Saleh study, another reason no difference in PI incidence was demonstrated may be because the preventive measures applied were not effective in preventing PIs (e.g. alternating air-mattresses (see [Qaseem](https://www.acpjournals.org/doi/epdf/10.7326/M14-1567) et al.)), however differences in PI preventive measures between groups were not described.  We have added some comments in our discussion section, to highlight these points, specifically regarding:   - The implementation of potentially ineffective preventive measures (Line 531) - The difficulties in making recommendations based on the poor-quality evidence (Line 562) |
|  | **Reviewer #4:** |  |
| **15** | This paper evaluates the prognostic accuracy and clinical effectiveness of risk prediction tools for pressure injuries (PIs). Through an umbrella review of 16 systematic reviews on prognostic accuracy and 10 on clinical effectiveness, it was found that commonly used tools like the Braden, Norton, and Waterlow scales have variable sensitivities (53%-97%) and specificities (46%-84%). Only two randomized trials examined the clinical effectiveness of the tools, and showed no significant impact on PI incidence. However, both of the trials were assessed as having high risk of bias. The study highlights the need for high-quality research to develop and validate more effective PI risk prediction tools. Below are my specific comments on the paper. | N/A |
| **16** | 1. I am concerned about the potential conflict of interest in this paper. The work was commissioned by Paul Hartmann AG (PHAG), a company specializing in surgical and medical instruments, and three of the eight authors have close ties with PHAG. It is noted that VV is an employee of PHAG, but this is not reflected in the affiliations listed in the paper. I believe the conflict-of-interest issues in this paper require careful scrutiny. | We appreciate your concerns and admire your commitment to ensuring research integrity. The current affiliations list does correctly disclose that VV is affiliated with PHAG; his affiliation is *“Evidence Generation Department, HARTMANN GROUP, Heidenheim, Germany”*. PHAG is part of HARTMANN GROUP, and we will make sure this is clarified in our paper. Regarding THB and ES, we believe we have been transparent with the potential conflicts of interest, as can be seen on our declarations page. It should be noted that these three authors were not involved in the screening, data extraction or analysis of results. These roles were conducted independently by authors at the University of Birmingham (UOB), who have no ties to PHAG. Additionally, the contract with UOB explicitly stated that authors at UOB had the freedom to publish results regardless of the findings. We hope that these clarifications address your concerns, and show that we are also committed to objective, unbiased and transparent research.  We have amended the Funding section (added: *“part of HARTMANN GROUP”)* and Conflicting Interests section (added: *“VV, ES and THB were not involved in data curation, screening, data extraction, analysis of results or writing of the original draft. These roles were conducted independently by authors at the University of Birmingham.”*) of our manuscript to address this issue. |
| **17** | 2. Introduction, line 76-82: Comment: the introduction briefly mentions the limitations of current risk prediction tools but could delve deeper into these issues to underscore the necessity for this study. For instance, the authors could elaborate on specific methodological flaws in existing models and why these flaws justify the need for the current review study. | We have reworded some of the statements from our introduction regarding flaws in the development of existing risk prediction tools for PI:  *“…the methodology underlying their development is not always explicit, with scales in routine clinical usage apparently based on epidemiological evidence and* *clinical judgment about predictors that may not meet accepted principles for the development and reporting of risk prediction models.^5^*” (Line 116-119)  However, we believe that the introduction is not the most appropriate section to expand on these points any further. One of the primary aims of our umbrella review is to obtain and summarise current evidence on PI risk prediction models, so we have attempted to outline the motivation for conducting the umbrella review in this section rather than addressing specific methodological flaws, some of which we were not aware of until we had begun the review. Therefore, instead we have expanded upon these methodological issues in the ‘Other existing evidence’ section of our discussion (see from Line 536), in the context of the evidence provided in our umbrella review. |
| **18** | 3. Method, lines 105-108: The authors searched MEDLINE, Embase, and CINAHL Plus EBSCO. However, I am curious why the Cochrane Library and Web of Science were not included in the literature search. Both are important databases for medical literature and should be considered. | We consider our search of three major electronic sources of medical literature, including one specifically targeted at nursing and allied health literature, to be sufficiently comprehensive. There is little to no empirical evidence of the benefits of more exhaustive searches for primary studies evaluating diagnostic or prognostic tests and less so for identification of systematic reviews. The reviewer is correct in their suggestion to search the Cochrane Library, however this is covered by our search of EPISTEMONIKOS (<https://www.epistemonikos.org/en/about_us/methods>). |
| **19** | 4. Method, lines 105-108: Additionally, I believe that a search conducted in January 2023 may be somewhat outdated. I suggest the authors conduct an updated search to include the most recent literature. | Thank you for this suggestion. We agree and have now updated the search to June 2024. We screened 898 articles and have included an additional 3 systematic reviews. |
| **20** | 5. Method, Lines 134-137: Could the authors elaborate on how they adapted AMSTAR-2 for the systematic review of risk prediction models in the main text? I believe a brief description is needed here. Additionally, is there any reference for the adaptation the authors made? | We adapted AMSTAR-2 using our own judgement and prior experience in conducting systematic reviews of diagnostic test and prognostic models. Full details are provided in Appendix 4. We have also added some more detail within the manuscript itself, as suggested (Line 182 and below).  *“For example, for reviews evaluating the prognostic accuracy of risk prediction tools we assessed eligibility criteria using the PIRT framework (Population, Index test, Reference standard, Target condition)^25^ and POII framework (Population, Outcome to be predicted, Intended use of model, Intended moment in time)^26^ and required methodological quality assessment to be conducted using validated and appropriate tools such as QUADAS^27^, QUADAS-2^28^ or PROBAST^29^. We omitted the AMSTAR-2 item relating to publication bias (Item 15) because of both the lack of empirical evidence for the effect of publication bias on test accuracy estimates, and limitations in statistical methods for identifying publication bias.^14^{Cochrane DTA handbook}.”* |
| **21** | 6. Method, Lines 138-153: The authors used a narrative synthesis approach with priority given to systematic reviews meeting certain criteria. I am curious why the authors did not conduct any statistical synthesis for tools that are examined across multiple studies. I believe that some statistical synthesis of the evidence for commonly used tools could be quite helpful. | The reviewer is correct in that there is contrasting guidance available regarding re-analysis of data in overview reviews (e.g. BMJ paper compared to Cochrane guidelines). For our review, we opted to follow Cochrane guidance for overview reviews and made the decision not to undertake additional analysis. We have outlined our rationale for this decision in our response to Editorial comment #1 above. |
|  | **Reviewer #5:** |  |
| **22** | Thank you for allowing me to review your excellent manuscript. Please see attached for my comments and recommendations.  Thank you for allowing me to review your excellent manuscript. As a member of the International Pressure Ulcer/Injury Guideline Governance Group (GGG), I have followed the literature related to pressure ulcer risk assessment closely and appreciate the amount of work that went into this umbrella review. | Thank you very much for your positive feedback and insightful comments. |
| **23** | **[Methodology]**  I have reviewed Chapter 5 of the **Cochrane Handbook on Umbrella Reviews** and believe you have followed their guidance carefully. | N/A |
| **24** | **[Methodology]**  In my initial review of the manuscript, I was concerned that there was **no exclusionary cut-off based on AMSTAR-2 assessments**. Some investigators exclude systematic reviews of poor quality. It appears that exclusions are primarily based on appropriateness of content and not quality. The Cochrane guidance seems to provide some latitude for including a broader range of systematic reviews in an umbrella review. | Thank you for raising this valid point. The inclusion of lower quality reviews does indeed risk introduction of bias in an overview review, however we believe our eligibility criteria to be justified. Firstly, we aimed to provide a thorough overview and critique of available evidence, both in terms of available reviews and in terms of available risk prediction tools. Secondly, we restricted inclusion to reviews that at least purported to be ‘systematic’ in nature, and finally, assessed the evidence in detail using AMSTAR-2. This serves not only to identify the higher quality and therefore more reliable reviews but also as an indicator to the wider readership of our paper of areas for improvement in the application of systematic review methods.  We have added the following text in our limitations section (Line 560): *“Additionally, we chose not to exclude reviews based on low AMSTAR-2 ratings to provide a comprehensive overview of all available evidence. However, by doing so, we acknowledge that many included reviews are of poor quality (with critically low confidence in 81%, 21/26, reviews), reducing the reliability of the evidence presented and the ability to make conclusions or recommendations based on this evidence.”* |
| **25** | **[Methodology]**  However, the result in this case (based on the quality of the systematic reviews available to you)… is that “*The quality of included reviews was generally poor***…”** (Line 219). You can’t change the quality of materials you had to work with; however, the **poor quality of source materials should “temper your conclusions”.**  Example: Your conclusion in the abstract states that *“There is* ***no*** *reliable evidence to suggest that using existing risk prediction tools effectively reduces the incidence of PIs.”* Such a strong, absolute statement should not be made based on poor quality systematic reviews. It is also not entirely true. Several studies reported lower PI incidence when risk assessment tools were used to guide prevention (Lovegrove, 2021; Chou, 2013; Health Quality Ontario, 2009). There is also evidence of improved intermediate outcomes (e.g., more preventive interventions) when risk assessment tools are used. See pages 19-20 for mixed results. **Can you modify “no” to “limited”? in this example and review the document for other absolute statements that may not be justified by the quality of systematic reviews or the findings of high-quality studies.** | Thank you – we have considered and incorporated your useful comments. Please see Line 63 for some rewording to slightly temper our conclusions, as suggested (“no” to “limited”).  After having reviewed our manuscript, we believe that the rest of our conclusions are appropriately written and have taken into account the poor quality of the evidence. Please see some examples below:   - *“It seems that limited comparative studies comparing the accuracy of different tools are available.”* (Line 486) - *“… restricting the conclusions that can be drawn from the limited evidence available.”* (Line 530) - *“The included systematic reviews used poor methodology and reporting, limiting our ability to reliably describe and evaluate their content.”* (Line 589) - *“Regarding clinical effectiveness, a reduction of PI incidence is unclear due the overall uncertainty and potential biases in available studies.”* (Line 596) |
| **26** | **Diagnostic vs. Prognostic Tests.**  There are subtle, but important differences between diagnostic and prognostic tests.  With diagnostic tests, the disease exists. You are determining the accuracy of a diagnostic test to detect an existing disease.  With prognostic tests, you are predicting the likelihood of future disease based on modifiable and non-modifiable risk factors. Prediction is not destiny. The true value of a prognostic test (e.g., risk assessment tool) is to identify and mitigate the modifiable risk factors thus preventing the disease. Under that clinical use scenario, lower sensitivity would be a desired outcome (i.e., If fewer subjects with a positive predictive test actually develop the disease…it may well indicate that your preventive measures were successful in preventing disease).  The umbrella review methodology draws on several references to diagnostic testing accuracy (DTA). **Please review how these references are used and applied.** **DTA principles may have some similarities with prognostic testing accuracy, but the intended clinical use and context needs to be explained and interpretation of results modified accordingly.**  In diagnosing an existing breast cancer, I would prefer both Sensitivity and Specificity to as close to 100% as possible.  **What is a desired, preferred or good Se and Sp for prognostic testing? Have we taken into account the use of the prognostic test in guiding prevention (seems like an intervening variable)? Have we truly studied the moderating effects of preventive interventions on the final (not predicted) outcome?**    References to DTA in the manuscript are listed below. **I would just ask that you discuss the limitations of DTA principles when applied to prognostic tests.**  *1) Preferred Reporting Items for Systematic Reviews and Meta-Analyses of Diagnostic Test Accuracy Studies’ (PRISMA-DTA)*  *2) Bossuyt PM, Reitsma JB, Bruns DE, et al. The STARD statement for reporting studies of diagnostic 725 accuracy: explanation and elaboration. Ann Intern Med 2003;138(1):W1-12. doi: 726 10.7326/0003-4819-138-1-200301070-00012-w1*  *3) All of your references for diagnostic testing accuracy seem to apply to diagnostic testing only and not diagnostic and prognostic testing.*  **I would just ask that you review your manuscript to see if you have fairly applied principles of diagnostic testing accuracy to prognostic testing accuracy. Have you stated the limitations?** Your reader is left with the impression that good, reliable, valid, accurate prognostic test should have high Se, Sp, AUROC values. I would argue that is not only not feasible but may not be the best measure of clinical effectiveness. | This is a very important comment which we have addressed in several places in the manuscript.  In the Introduction section, we have more directly addressed the clinical value of risk assessment in guiding decisions about preventive measures and their intensity according to risk (Line 109).  We do acknowledge that there is a reliance on DTA principles throughout the review however, we would argue that this largely reflects the body of evidence available and a lack of available guidance for reviews evaluating prognostic accuracy. For the most part, available reviews have considered the risk prediction tools as ‘tests’ applied at a single point in time at a particular threshold for risk of PI, with the resulting 2x2 contingency tables used to calculate measures such as sensitivity and specificity. Although we did not access the primary study publications, it seems likely that the same paradigm has been used in the primary literature. Importantly, although a large proportion of reviews set out to evaluate the ‘predictive’ validity of PI risk assessment tools (18/19), 16 of these relied on DTA principles, without any apparent consideration of the time interval between test application and the occurrence of the outcome. We attempted to address this aspect in our AMSTAR-2 adaption (see response to comment 20), but have also incorporated these valid points into our discussion section (see final paragraph Line 579 onwards). We also made sure to reference RoB tools developed more recently specifically for evaluating prognostic accuracy studies, such as QUAPAS (2022).  We are not aware of any guidance regarding target sensitivity or specificity for prognostic accuracy, and indeed much of the available guidance for conducting systematic reviews in the prognostic space centres around appropriate evaluation of risk prediction model performance and reporting (e.g. TRIPOD-SRMA), hence the reliance on reporting standards such as PRISMA-DTA.  We have also added further discussion of the limitations of DTA principles in the discussion, and pointed to alternative methods that allow the cost of misclassification to be explicitly considered (Line 497).  Sensitivity and specificity can be optimised by methods which directly consider the cost of misclassification, including both the harms associated with applying more intensive prevention in those with a false positive result and the benefits of preventive measures in those with a true positive result. One solution to determine the preventive treatment threshold risk is through net benefit calculations,^95 96^ which can be visualised in decision curves and are common in prognostic research. These calculations can assist in providing a balanced use of resources while maximising positive health outcomes, such as lowering incidence of PI. These discussions have been added to our manuscript (Line 503). |
| **27** | **I don’t think we have answered the basic question: Why do we do pressure injury risk assessments? Is our primary goal to PREDICT? OR Is our primary goal to use the assessment findings (especially modifiable risks) to PREVENT pressure injuries? Our criterion for good prognostic test is very different depending on which question you ask. I realize that the science has focused on predictive accuracy. I just wonder if our time is best spent continuing down that pathway. We will never find the perfect tool. There is a lot we can do in studying how risk assessment tools can be utilized in guiding prevention and producing better outcomes.** | Thank you for your comment. In terms of evaluating existing risk prediction tools, after accuracy has been ascertained using appropriate metrics, we agree that the focus should be placed on whether the tool can help guide prevention to improve patient outcomes (through changes in preventive management based on the results of risk assessment tools), including the reduction of PIs.  In terms of developing new risk prediction tools/models, if a risk prediction tool is unable to change outcomes with existing preventive interventions and is not appropriately “nested” in clinical workflows, then it is worth nothing to develop a new tool, even according to the best development and validation recommendations. Therefore, model developers need to check the expected value of prognosis and how the tool can guide prevention when employed in practice, before planning model development. If it’s determined that there is no value in predicting certain outcomes – that brings into question whether the model should even be developed [[PROGRESS 4](https://doi.org/10.1136/bmj.e5793)].  Moreover, as correctly mentioned, identifying and mitigating modifiable risk factors can prevent the disease. Although many predictive factors, especially those identified through machine learning techniques, can be useful for more accurate predictions, not all have a causal relationship with the prediction target. Therefore, not every predictor will be a clinical risk modifier. To achieve this, additional effort is needed in the development of risk assessment tools to extract predictors that are risk modifiers and provide end-users with this information. This approach assumes personalised risk assessment, making the goal to build a model that not only predicts outcomes accurately but also incorporates causal relationships, making the predictions more interpretable and actionable. Some developments exist in this direction, but future validation of these methods is needed.    Lastly, all previous discussions assume that we are developing the risk assessment model to guide prevention measures. However, risk assessment can also be developed for enriching study design (e.g., as a means of recruiting only high-risk patients to studies evaluating preventive measures). In such cases, a different approach and optimisation of performance metrics are needed. Therefore, risk prediction models need to pre-specify their application before development to allow their clinical utility for a given context to be addressed.  We have addressed these points in our discussion (Line 510). |
| **28** | **I had some concerns about the general characterization of pressure injury risk assessment tools starting at line 76. It ignores the body of work that uses epidemiological evidence to develop tools that identify modifiable risk factors that nurses can address. I am not sure you can say “***many are limited by reliance on subjective clinical judgment” when interrater reliability is high in trained staff.* It is true that the tools do not contain all risk factors….but they were never designed to. The impetus for risk assessment tools was to translate known science into a quick and reliable assessment tool that guides risk-based prevention of the modifiable risk factors amenable to nursing interventions. | Thank you for your comment. Regarding the statement **“***many are limited by reliance on subjective clinical judgment”*, we agree with your points and agree that our original wording may have caused some confusion. In fact, our intention was to highlight that many clinical scales are developed using clinical judgement, rather than suggesting that subjective clinical judgement is a limitation in the application and interpretation of these tools. In order to more clearly portray this meaning, we have reworded this section as follows: *“Numerous clinical assessment scales and statistical risk prediction models for assessing the risk of PI are available however, the methodology underlying their development is not always explicit, with scales in routine clinical usage apparently based on epidemiological evidence and* *clinical judgment about predictors that may not meet accepted principles for the development and reporting of risk prediction models.^5^” (Line 115)* |
| **29** | I have followed the **clinical effectiveness studies** carefully over the years. The underlying premise of many of these studies is flawed. No clinician would claim that the simple act of assessing cardiovascular risk factors would prevent myocardial infarctions. Or that taking your blood pressure everyday will eliminate your risk of stroke. YOU NEED TO DO SOMETHING WITH THE ASSESSMENT INFORMATION TO MITIGATE THE RISKS. Too few of the clinical effectiveness studies describe how the risk assessments are used to guide prevention.  The Agency for Healthcare Policy and Research developed a pressure injury prevention guide many years ago that recommends preventive interventions be designed to address both the type of level of risk on Braden Scale subscore (and that additional risk factors be considered). Preventive interventions are different for immobility vs. moisture vs. nutritional deficits. The intensity of the intervention is aligned with the level of risk, with more intense and expensive interventions reserved for higher risk subscale scores. A matrix of interventions has been developed based on type and level of risk and incorporated into computerized care planning systems. In many settings this has been more effective than determining a “risk score” and applying a bundle of interventions. Regrettably, this common practice has been documented in the quality improvement and implementation research, but not in randomized clinical trials. This risk-based prevention strategy may not be the best fit in every setting. However, it does illustrate that risk assessment tools can be used to guide risk-based prevention plans. **To truly understand the clinical effectiveness (or not) of risk assessment tools, you need to describe how the assessment findings were used to guide risk-based prevention.** | We agree with the points reviewer #5 has raised here, in particular about the need to do something with the risk assessment information to be able to mitigate the risks.  The first step in evaluating the clinical value of risk assessment is to identify the preventive measure decisions that the risk assessment is intended to guide. In this population at risk, we cannot assume a common overall baseline risk due to high variability. Therefore, as is known from prognostic research in general, the value of conducting risk assessment will vary with the pretest probability of developing the condition. Additionally, evidence synthesis on the efficacy of pressure injury preventive measures shows that no single intervention is effective on its own; rather, a combination of several interventions is required. Health economic models have suggested that providing baseline preventive interventions for all with daily risk assessments is more cost-effective than either a less standardised prevention protocol or a targeted risk-stratified prevention strategy. Nevertheless, the stratification of patients by risk could further improve outcomes by triggering additional types of or more intensive preventive interventions (e.g., more frequent repositioning), based on the results of the risk assessment. We have elaborated on the need for effective preventive measures, in particular multicomponent interventions, in our introduction (see Line 106-114).  We have also added the following text to our discussion:  *“We should recognise that effectiveness outcomes from using a risk prediction tool depend on the timely implementation of effective preventative measures, a step that is frequently poorly described in studies evaluating the effectiveness of risk assessment tools, limiting the conclusions that can be drawn from the evidence available.” (Line 527)*  We acknowledge that it is necessary to consider how risk assessment information is applied in practice to guide risk-based preventive interventions, in order to fully understand the clinical effectiveness of the risk assessment/prediction tools. We hope our amended statements above reflect this. |
| **30** | **Conclusion:** I have great respect for the work you have completed with this umbrella review. Your work summarizes what is known (from relatively poor-quality systematic reviews) but I am not sure it provides new insights or direction for future research. You suggest that further research is needed on clinical effectiveness but only once promising prediction tools have been developed and validated. How do we accomplish this? What are the criteria for promising prediction tools and methods to determine clinical effectiveness? What is a good prediction tool/risk assessment tool? Are designs and methodologies from implementation science more appropriate to demonstrate clinical effectiveness than RCTs designed to demonstrate efficacy?  **Are we asking the right questions?**   - **Is our primary objective in using risk assessment tools to PREDICT or PREVENT pressure injuries?**   **Can we truly measure “clinical effectiveness” if we don’t consider how the risk assessment tool is being used in the clinical setting?**  *My fear is that if we continue down the same path of investigation, we will find ourselves with the same inadequate and disappointing answers. I would be willing to write a short editorial commentary that acknowledges your work….. but also asks:* ***“Are we asking the right questions?”*** *Your manuscript is excellent and I am recommending publication with minor revisions. I think my questions and your response would generate excellent ideas for further direction in research and systematic reviews. I am willing to write a commentary if that is of interest to the editors and authors. Thank you for allowing me to review this manuscript.* | We appreciate all of reviewer #5’s comments and constructive feedback.  Following on from our discussion above and our manuscript’s discussion section, we recommend that researchers developing prediction models should follow appropriate guidance (e.g. [PROGRESS 3](https://journals.plos.org/plosmedicine/article?id=10.1371/journal.pmed.1001381)), ensure transparency with their reporting (TRIPOD), ensure tools are validated externally, and think about where in the clinical pipeline their model/tool can be used effectively (see [Reilly et al.](https://www.acpjournals.org/doi/10.7326/0003-4819-144-3-200602070-00009) and [Moons et al.](https://www.bmj.com/content/338/bmj.b606)).  Regarding whether the primary objective is to predict or prevent pressure injuries, we don’t think this is a strict dichotomy. We believe that the right question is how we should develop the risk assessment tool to accurately guide preventive measures.  Reviewer #5 has posed many challenging, but important, questions and, hopefully, the changes we have made based on comments 22-29 have addressed the reviewer’s concerns.  We would like to encourage the reviewer to write a commentary piece stemming from our work and this dialogue, as this may lead to wider research impact. With reviewer #5’s expertise and experience, we believe their points would be a significant addition to the discussion on the evaluation of prognostic models/tools, in the context of PI prevention and management. |
