# Supplementary material for: Accuracy and clinical effectiveness of risk prediction tools for pressure injury occurrence: An umbrella review
Source: PLoS Med. 2025 Feb 6;22(2):e1004518. doi: 10.1371/journal.pmed.1004518 (PMC11844857; doi:10.1371/journal.pmed.1004518)
Supplement: Attachment — Submitted filename: Reviewer 5_2nd review.docx [file pmed.1004518.s004.docx]

Thank you for your thorough review of my previous comments. I appreciate your detailed and thoughtful responses as well as the modifications you made to your manuscript. Ultimately, I think we are still left with a core conundrum when evaluating the *accuracy and clinical effectiveness of risk prediction tools for pressure injury occurrence.*  **Accuracy** is defined in the literature as the prognostic accuracy of the tool, yet **clinical effectiveness** (i.e. preventing pressure injuries) is highly dependent on mitigating the modifiable risk factors identified by the tool. It’s a Catch-22 situation. When risk assessment tools are used as a basis for risk-based prevention, an effective tool should demonstrate less prognostic accuracy than that reported in the original studies of tool validity and reliability.

**Are we asking the right questions?**

You noted that “…scales in routine clinical usage [are] based on epidemiological evidence and clinical judgment about predictors that may not meet accepted principles for the development and reporting of risk prediction models.”

1. **What specific principles for development and reporting are currently missing?**
2. **Should our primary focus be on improving risk prediction models? This is an important research goal; however, is it the best approach to clinical effectiveness?**

The risk assessment tools most commonly used in clinical practice are based on recent epidemiological evidence balanced by the clinical and scientific judgment needed to identify plausible links between the risk factor and current knowledge of pressure injury etiology.^1^  Additionally, these tools focus on modifiable risk factors; provide operational definitions of key risk factors that can be accurately assessed by bedside care providers; identify subscale scores that can be used for risk based prevention planning (in conjunction with additional risk factors); and provide a framework for assessment and a minimum auditable standard.^2^  There is a substantial body of foundational literature examining the psychometric properties of these scales. ^2^

3. **How can we design studies that capture the complex interplay of factors when risk assessment tools are used as a basis for prevention? Can we make tool-directed prevention part of the design?**
4. **Is it even possible to establish prognostic accuracy of tools under current clinical use scenarios?**

One of the most intriguing aspects of your paper was your excellent synthesis of the recent literature on Machine Learning (ML) based predictive models. If our primary goal is accurate prediction of pressure injuries, this strategy is promising. The high risk of bias and relative lack of external validation in other data sets are limitations that can be addressed. Of greater concern is how we integrate ML tools into clinical practice. Drawing on our past experiences with clinical applications of predictive tools….

5. **Can we create a human interface of clinicians and researchers to assess the plausibility of linkages between ML-identified risk factors and pressure injury outcomes?**
6. **How do we use ML risk predictions to support effective prevention?**  A simple score indicating high risk may serve as a discouraging disincentive to preventive efforts in a seemingly hopeless situation. However, prediction is not destiny! Can ML tools identify the modifiable risk factors to guide more effective prevention?

I look forward to our continued dialogue as we address both accuracy and clinical effectiveness in our use of pressure injury risk assessment tools.

Respectfully submitted,

Janet Cuddigan, PhD, RN, FAAN

Professor Emeritus

University of Nebraska Medical Center

Omaha, NE USA

**References**

1. Gefen, A., Brienza, D. M., Cuddigan, J., Haesler, E., & Kottner, J. (2021). Our contemporary understanding of the aetiology of pressure ulcers/pressure injuries. *Int Wound J*. <https://doi.org/10.1111/iwj.13667>
2. European Pressure Ulcer Advisory Panel, National Pressure Injury Advisory Panel, Pan Pacific Pressure Injury Alliance. *Risk Factors and Risk Assessment.* In Prevention and Treatment of Pressure Ulcers/Injuries: Clinical Practice Guideline. The International Guideline 2019. E. Haesler (editor).
