# Supplementary material for: Accuracy and clinical effectiveness of risk prediction tools for pressure injury occurrence: An umbrella review
Source: PLoS Med. 2025 Feb 6;22(2):e1004518. doi: 10.1371/journal.pmed.1004518 (PMC11844857; doi:10.1371/journal.pmed.1004518)
Supplement: Attachment — Submitted filename: PMed Editor+reviewer comments 1124 +JDBH.docx [file pmed.1004518.s005.docx]

PLOS Medicine editor & reviewer comments for: ***"*** ***Accuracy and clinical effectiveness of risk prediction tools for pressure injury occurrence: An umbrella review”*** (PMEDICINE-D-24-00795R2)

|  | **Editor comments** | | **Author replies** |  |
| --- | --- | --- | --- | --- |
|  | Thank you very much for re-submitting your manuscript "Accuracy and clinical effectiveness of risk prediction tools for pressure injury occurrence: An umbrella review" (PMEDICINE-D-24-00795R2) for review by PLOS Medicine.  Thank you for your detailed response to the editors' and reviewers' comments. I have discussed the paper with my colleagues, and it has also been seen again by two of the original reviewers. The changes made to the paper were mostly satisfactory to the reviewers. As such, we intend to accept the paper for publication, pending your attention to the reviewers' and editors' comments below in a further revision. When submitting your revised paper, please once again include a detailed point-by-point response to the editorial comments. | | N/A |  |
| **Section / number** | | **Editorial request** | **Author replies** | |
| **GENERAL** | |  |  | |
| **1** | | The manuscript contains many abbreviations, please check carefully throughout the main text to ensure that abbreviations have been introduced appropriately. | Thank you. As well as the abbreviations mentioned in the comments below, we have also now introduced NICE, PROBAST, QUADAS and AHRQ in the text at first use. | |
| **CODE AVAILABILITY** | |  |  | |
| **1** | | We expect all researchers with submissions to PLOS in which author-generated code underpins the findings in the manuscript to make all author-generated code available without restrictions upon publication of the work. In cases where code is central to the manuscript, we may require the code to be made available as a condition of publication. Authors are responsible for ensuring that the code is reusable and well documented. Please make any custom code available, either as part of your data deposition or as a supplementary file. Please add a sentence to your data availability statement regarding any code used in the study, e.g. "The code used in the analysis is available from Github [URL] and archived in Zenodo [DOI link]" Please review our guidelines at <https://journals.plos.org/plosmedicine/s/materials-software-and-code-sharing> and ensure that your code is shared in a way that follows best practice and facilitates reproducibility and reuse. Because Github depositions can be readily changed or deleted, we encourage you to make a permanent DOI'd copy (e.g. in Zenodo) and provide the URL. | This is not applicable, as no author-generated code was produced or utilised for this project. See Lines 630-631. | |
| **ABSTRACT** | |  |  | |
| **1** | | Please provide the beginning and end dates of your search. | See Lines 47 and 48. | |
| **2** | | Please provide the eligibility criteria and types of study designs included. | See Lines 44-47. | |
| **3** | | We suggest mentioning that 26 systematic reviews met all eligibility criteria (similar to lines 213-215). | See Line 50. | |
| **4** | | In the last sentence of the Abstract Methods and Findings section, please describe the main limitation(s) of the study's methodology. | See Lines 63-68. | |
| **AUTHOR SUMMARY** | |  |  | |
| **1** | | In the final bullet point of 'What Do These Findings Mean?', please include the main limitations of the study in non-technical language. | See Lines 109-112. | |
| **INTRODUCTION** | |  |  | |
| **1** | | Please cite the reference numbers in square brackets. Citations should precede punctuation. Please revise throughout. | We have amended this throughout. | |
| **2** | | l.104: Please define ‘US’ at first use. | See Line 117. | |
| **3** | | l.120: Please define ‘UK’ at first use. | See Lines 133-134. | |
| **4** | | l.123: Please exchange the word ‘elderly’ with ‘older’. | See Lines 137 and 139. | |
| **5** | | l.127: Please define ‘BMI’ at first use. | See Lines 141-142. | |
| **6** | | l.128: The terms gender and sex are not interchangeable (as discussed in  <https://www.who.int/health-topics/gender#tab=tab_1)>; please use the appropriate term. | We have amended this to “sex”, rather than “gender”; see Line 142. | |
| **METHODS & RESULTS** | |  |  | |
| **1** | | l.163: Please specify “adult patients”, e.g., by providing an age range. | We have provided further detail on our inclusion criteria here (see Lines 179-182). We understand the term ‘adult’ to refer to individuals aged 18 and over, but accepted individual study definitions of adult and also included studies in which ‘adult’ was not defined. We also excluded any studies focusing on tools specifically developed for paediatric populations (e.g. Braden-Q scale). | |
| **2** | | l.243: "(6, 55%)" - we suggest to consistently report the data as (6/11, 55%), i.e. numerator/denominator and percentage. Please revise throughout. | We have amended throughout, so that the data are reported as numerator/denominator and percentage. | |
| **3** | | l.249: Please define ‘JBI’ and ‘CASP’ first use. | See Lines 271-272. | |
| **4** | | Table 2: Please consider avoiding the use of red and green in order to make your figure more accessible to those with colour blindness | Thank you for your suggestion. Although still using red and green (due to the ‘traffic light’ nature of the figure), we have adjusted the colour shades so that the red is darker, and the green is now lighter. This should help in terms of differentiating between the two colours. | |
| **5** | | l.302: Please define ‘CI’ at first use. Throughout, we suggest reporting statistical information as follows to improve clarity for the reader "22% (95% CI [13%,28%]; p</=)". When reporting 95% CIs please separate upper and lower bounds with commas instead of hyphens as the latter can be confused with reporting of negative values. | We have now defined CI at first use (see Line 329). We have amended our reporting of CIs, as suggested. Note that p values were not given as they were either not applicable (e.g. for sensitivities) or were not reported (e.g. for risk ratios). | |
| **6** | | Table 3: Please define ‘PROBAST’ below the table. | See Table 3 caption. | |
| **7** | | Table 4: Please define ‘PI’ below the table. | See Table 4 caption. | |
| **8** | | Table 5: Please check whether the abbreviations ‘CASP’ and ‘S.S.’ appear in the table and remove accordingly. | Thank you for bringing this to our attention. We have removed these descriptions. | |
| **DISCUSSION** | |  |  | |
| **1** | | Please remove any subheadings from the discussion section, including the ‘conclusion’ subheading. | We have removed the subheadings, as requested. | |
| **2** | | l.571: Please define ‘DTA’ at first use. | See Line 510. | |
| **REFERENCES** | |  |  | |
| **1** | | PLOS uses the numbered citation (citation-sequence) method and first six authors, et al. | The references have been amended to present the first six authors, et al. | |
| **2** | | Please ensure that journal name abbreviations match those found in the National Center for Biotechnology Information (NCBI) databases (<http://www.ncbi.nlm.nih.gov/nlmcatalog/journals>), and are appropriately formatted and capitalised. | This has been done for all references. | |
| **3** | | Where website addresses are cited, please include the complete URL and specify the date of access (e.g. [accessed: 12/06/2024]). | This has been done for all references, where applicable. | |
| **4** | | Please also see <https://journals.plos.org/plosmedicine/s/submission-guidelines#loc-references> for further details on reference formatting. | N/A | |
| **SUPPLEMENTARY MATERIAL** | |  |  | |
| **1** | | Please note that supplementary material will be posted as supplied by the authors. | N/A | |
| **2** | | In the published article, supporting information files are accessed only through a hyperlink attached to the captions. For this reason, you must list captions at the end of your manuscript file. You may include a caption within the supporting information file itself, as long as that caption is also provided in the manuscript file. Do not submit a separate caption file.  When SI files are contained with a single file: Please label the file as ‘S1 Supporting Information’. Please apply alphabetical labelling to each table and figure contained within the S1 file. For example, ‘Fig A’ to ‘Fig Z’ and ‘Table A’ to ‘Table Z’. Plain text does not need to be labelled and can just be given a title as necessary. For example, ‘Statistical Analysis Plan’. Please cite tables/figures as ‘Fig A in S1 Supporting Information’ and/or ‘Table A in S1 Supporting Information’, for example. Please cite plain text as, ‘Statistical Analysis Plan in S1 Supporting Information’, for example.  When SI files are uploaded as separate files: Please label tables as ‘S1 Table’ (so on) and figures as ‘S1 Fig’ (and so on). Any additional documents (protocols/analysis plans etc.) can be labelled as ‘S1 Protocol’, for example. Please cite items as exactly as labelled. | The caption for the supplementary file, on page 29, Line 629, is now labelled as “S1 Supporting Information”.  The titles of figures have been renamed, as to use letters rather than numbers. | |
| **SOCIAL MEDIA** | |  |  | |
| **1** | | To help us extend the reach of your research, please provide any X (formerly known as Twitter) handle(s) that would be appropriate to tag, including your own, your co-authors’, your institution, funder, or lab. Please enter in the submission form any handles you wish to be included when we post about this paper. | SOCIAL MEDIA  Please use the X handle @UoB_BESTEAM. | |
| **GENERAL EDITORIAL REQUESTS** | |  |  | |
| **1** | | We ask every co-author listed on the manuscript to fill in a contributing author statement. If any of the co-authors have not filled in the statement, we will remind them to do so when the paper is revised. If all statements are not completed in a timely fashion this could hold up the re-review process. Should there be a problem getting one of your co-authors to fill in a statement we will be in contact. YOU MUST NOT ADD OR REMOVE AUTHORS UNLESS YOU HAVE ALERTED THE EDITOR HANDLING THE MANUSCRIPT TO THE CHANGE AND THEY SPECIFICALLY HAVE AGREED TO IT. | Thank you for the reminder – we have also contacted co-authors to ensure this will be done in a timely manner. | |
| **2** | | Please ensure that the paper adheres to the PLOS Data Availability Policy (see <http://journals.plos.org/plosmedicine/s/data-availability)>, which requires that all data underlying the study's findings be provided in a repository or as Supporting Information. For data residing with a third party, authors are required to provide instructions with contact information for obtaining the data. PLOS journals do not allow statements supported by "data not shown" or "unpublished results." For such statements, authors must provide supporting data or cite public sources that include it. | All data is publicly available due to the nature of the review work, as we have relied on data available in published work. All data produced in the present work are contained in the manuscript and supplementary file. | |
| **3** | | Please review your reference list to ensure that it is complete and correct. If you have cited papers that have been retracted, please include the rationale for doing so in the manuscript text, or remove these references and replace them with relevant current references. Any changes to the reference list should be mentioned in the rebuttal letter that accompanies your revised manuscript. | N/A | |

| **Comment number** | **Reviewer comments** | **Author replies** |
| --- | --- | --- |
|  | **Reviewer #4:** |  |
| **1** | I believe the authors have sufficiently addressed and responded to both my and the other reviewers' comments and questions. I am satisfied with the revisions made to the manuscript and find the responses to be thorough. As a result, I consider the paper to be acceptable for publication. | We thank Reviewer #4 for their valuable feedback – we are glad that they consider our paper acceptable for publication. |
|  | **Reviewer #5:** |  |
| **2** | This is an excellent paper and should generate discussion within the international pressure injury/ulcer community.   To that end, I wrote a commentary both applauding your work but also identifying unanswered questions. Are we asking the right questions?  I don't expect a response with solutions.  You have beautifully summarized "what we know".  I am asking "what do we still need to know?"  Please see attached for Reviewer Commentary.  I recommended acceptance for publication with minor revisions.  My source of continued concern can be found in lines 115-133 of the manuscript.  Lines 115-129 provide a historic perspective of the initial development of the major risk assessment tools.  This is fine....but it is limited in scope and sample size and fails to mention that there is a substantial body of literature evaluating the psychometric properties of these tools.  Line 130 draws an immediate negative inference about the tools in stating "Despite the apparent lack of reporting of now standard methods for development....."  I had difficulty finding Reference 7 in PubMed or Medline but eventually found it in EMBASE.  I noted that preprint I located through EMBASE ....was not certified for peer review.  I have 3 concerns: 1.  The negative inference mentioned above... 2. The reference to "now standard methods"  which are not explained and were not available when psychometric testing of these tools was conducted. 3. The emphasis on lack of external validity within the context of these tools.  This may apply to ML-generated risk prediction tools, but it doesn't really apply to tools that have been tested in thousands of clinical settings world-wide.  My recommendations is to reorder/revise the text starting at line 130 as follows....  There is a considerable body of evidence evaluating the psychometric properties and clinical utility of available risk prediction tools, much of which has been synthesised in systematic reviews and meta-analyses (7). However, there is an apparent lack of reporting of now standard methods for development and validation of risk prediction tools. | We thank Reviewer #5 for their feedback and suggestions. First of all, we have taken aboard the reviewer’s suggestion and revised the sentence from the introduction. See Lines 144-147 for the revised text.  To respond to the reviewer’s concerns, Reference 7 is an umbrella review, conducted alongside this current umbrella review, with more of a focus on the development and validation methods of risk prediction tools for PI. It is currently under consideration for publication by another journal. We hope it will be published soon, so it can add further context to these points.  We would also like to highlight the important distinction between external validation of prognostic models (assessing model discrimination and calibration in an external data set) and calculating prognostic accuracy of a risk prediction tool at a given threshold for high/low risk (undertaken using an external data set). We were referring to the former description here, which as the reviewer mentions, is applicable to machine learning models, as well as statistically derived prediction models.  Reviewer #5 makes a good point, that standards for developing prediction tools have evolved over time and were not available for some of the earlier developed tools identified by these reviews. However, using the current standards and recommendations (e.g. [PROGRESS 3](https://journals.plos.org/plosmedicine/article?id=10.1371/journal.pmed.1001381)), we can still identify and evaluate potential issues and biases, even for tools that were developed prior to the establishment of the current standards. Of course, it is important to acknowledge this fact, but the findings can still help us to identify areas for future research or development of newer tools (when many of them are based on or incorporate traditional scales, e.g. the Braden score, 1987). We do also comment on the recency of the reporting standards for diagnostic tests and prediction models (e.g. [STARD](https://www.bmj.com/content/351/bmj.h5527) and [TRIPOD](https://www.acpjournals.org/doi/10.7326/M14-0698)) within the companion umbrella review, and how this would have impacted to quality of reporting. |
| **3** | Thank you for your thorough review of my previous comments. I appreciate your detailed and thoughtful responses as well as the modifications you made to your manuscript. Ultimately, I think we are still left with a core conundrum when evaluating the accuracy and clinical effectiveness of risk prediction tools for pressure injury occurrence. Accuracy is defined in the literature as the prognostic accuracy of the tool, yet clinical effectiveness (i.e. preventing pressure injuries) is highly dependent on mitigating the modifiable risk factors identified by the tool. It’s a Catch-22 situation. When risk assessment tools are used as a basis for risk-based prevention, an effective tool should demonstrate less prognostic accuracy than that reported in the original studies of tool validity and reliability.  **Are we asking the right questions?**  You noted that “…scales in routine clinical usage [are] based on epidemiological evidence and clinical judgment about predictors that may not meet accepted principles for the development and reporting of risk prediction models.”  **1. What specific principles for development and reporting are currently missing?**  **2. Should our primary focus be on improving risk prediction models? This is an important research goal; however, is it the best approach to clinical effectiveness?**  The risk assessment tools most commonly used in clinical practice are based on recent epidemiological evidence balanced by the clinical and scientific judgment needed to identify plausible links between the risk factor and current knowledge of pressure injury etiology.1 Additionally, these tools focus on modifiable risk factors; provide operational definitions of key risk factors that can be accurately assessed by bedside care providers; identify subscale scores that can be used for risk based prevention planning (in conjunction with additional risk factors); and provide a framework for assessment and a minimum auditable standard.2 There is a substantial body of foundational literature examining the psychometric properties of these scales.2  **3. How can we design studies that capture the complex interplay of factors when risk assessment tools are used as a basis for prevention? Can we make tool-directed prevention part of the design?**  **4. Is it even possible to establish prognostic accuracy of tools under current clinical use scenarios?**  One of the most intriguing aspects of your paper was your excellent synthesis of the recent literature on Machine Learning (ML) based predictive models. If our primary goal is accurate prediction of pressure injuries, this strategy is promising. The high risk of bias and relative lack of external validation in other data sets are limitations that can be addressed. Of greater concern is how we integrate ML tools into clinical practice. Drawing on our past experiences with clinical applications of predictive tools….  **5. Can we create a human interface of clinicians and researchers to assess the plausibility of linkages between ML-identified risk factors and pressure injury outcomes?**  **6. How do we use ML risk predictions to support effective prevention?**  A simple score indicating high risk may serve as a discouraging disincentive to preventive efforts in a seemingly hopeless situation. However, prediction is not destiny! Can ML tools identify the modifiable risk factors to guide more effective prevention?  I look forward to our continued dialogue as we address both accuracy and clinical effectiveness in our use of pressure injury risk assessment tools. | We thank the reviewer for their very thoughtful inputs and discussions, which have led to significant improvements in our paper. We look forward to seeing where this discourse leads. We do not think any specific changes to the paper are required in response. |
